# Quantification of the effects of n-π* interactions on the H-bonding properties of amide groups†

**DOI:** 10.1039/d4sc08331h

**Published:** 2025-04-03

**Authors:** Fergal E. Hanna, Andrew D. Bond, Christopher A. Hunter

**Affiliations:** a Yusuf Hamied Department of Chemistry, University of Cambridge Lensfield Road Cambridge CB2 1EW UK herchelsmith.orgchem@ch.cam.ac.uk

## Abstract

The n-π* interaction is postulated to play a key role in the folding of proteins, especially in proline-rich structures such as collagen, and cooperativity between H-bonding and the n-π* interaction has been proposed. In order to obtain experimental evidence for these cooperative effects, the H-bond acceptor properties of secondary amides with and without the capacity to form an intramolecular n-π* interaction were measured. UV-vis absorption and ^13^C NMR titrations were used to investigate the intermolecular H-bonded complexes formed with 2-methyl-4-nitro-phenol and perfluoro-*tert*-butanol, and hence quantify the H-bond acceptor properties of the amide carbonyl oxygens. For an *N*-acylproline derivative, the presence of an intramolecular n-π* interaction between two amide groups was confirmed by X-ray crystallography, but the solution titrations show that associated changes in the H-bond acceptor strength of the amide carbonyl oxygen group are negligible. The free energy contribution due to cooperativity between the intramolecular n-π* interaction and the intermolecular H-bond was found to be within the error of the experiment (<1 kJ mol^−1^). The results suggest that any contributions to the thermodynamic stability of folded proteins due to such cooperativity are small.

## Abstract 2

The free energy contribution due to cooperativity between an n–π* interaction and a H-bond was found to be within the experimental error, suggesting that associated contributions to the stability of folded proteins are small.

## Graphical abstract

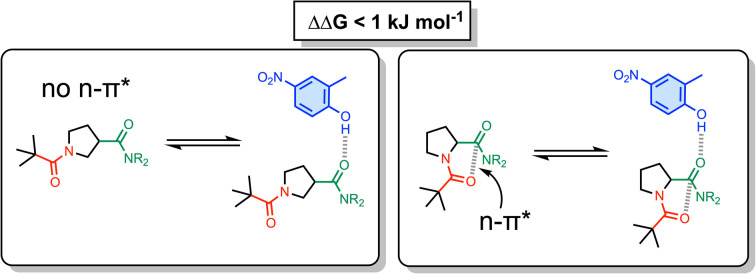

## Introduction

**Fig. 1:**
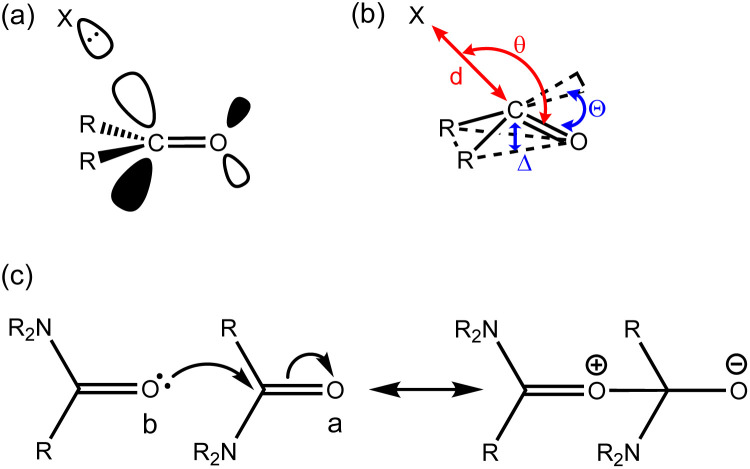
(a) An n-π* interaction between the lone pair on X and the π* orbital of the carbonyl group. (b) Parameters used to describe the relationship between the geometry of interaction (red) and pyramidalisation of the carbonyl group (blue). (c) Valence bond representation of the n-π* molecular orbital interaction between two amide groups: the electron acceptor carbonyl group is labelled a, and the electron donor carbonyl group is labelled b.

The n-π* interaction between a lone pair and a carbonyl π-system has been proposed to play an important role in protein folding, most notably in proline-rich helices like collagen (Fig. 1a),^1–3^ as well as in the structures of oligonucleotides,^4,5^ poly(lactic acid)^6^ and peptoids.^7^ Close contacts observed in X-ray crystal structures of small molecules such as aspirin^8,9^ and *N*-acyl homoserine lactones have been assigned as n-π* interactions,^10,11^ and the role of these interactions has been investigated in synthetic supramolecular materials.^12–18^ The geometrical parameters shown in Fig. 1b have been proposed for identifying the presence of an n-π* interaction: the distance between the interacting atoms is less than the sum of the van der Waals radii (*d* < 3.2 Å), the angle of approach of the lone pair aligns with the π* orbital (90 < θ < 110°), and the carbonyl group is non-planar (*Δ* > 0, *Θ* > 0).^2^

The magnitude of an n-π* interaction between two amide groups has been estimated at 1 kJ mol^−1^, based on a combination of *ab initio* calculations and experimental measurements of *cis*–*trans* equilibrium constants for *N*-acyl proline derivatives.^19,20^ Both covalent molecular orbital interactions and non-covalent electrostatic interactions are implicated.^2,21–25^Fig. 1c shows an extreme valence bond representation of the molecular orbital interaction between two amides with complete transfer of two electrons between the molecules. In reality, the extent of electron transfer in the n-π* interaction is very small, but the representation in Fig. 1c makes the point that any molecular orbital interaction should change the polarity of the interacting groups.

*Ab initio* calculations predict that there is cooperativity between n-π* interactions and the H-bonding properties of amide groups: the carbonyl oxygen that donates the lone pair (labelled b in Fig. 1c) becomes a worse H-bond acceptor,^3,26^ and the carbonyl oxygen of the π* acceptor (labelled a in Fig. 1c) becomes a better H-bond acceptor.^27,28^ The magnitudes of these effects have important implications for understanding cooperative phenomena in processes like protein folding. Here we use a molecular system with two amide groups conformationally locked in a geometry consistent with an intramolecular n-π* interaction to investigate these cooperative effects. We find that differences in the H-bond acceptor properties of the oxygen of the π* carbonyl group are too small to be detected, when compared with unperturbed amide groups.

**Fig. 2:**
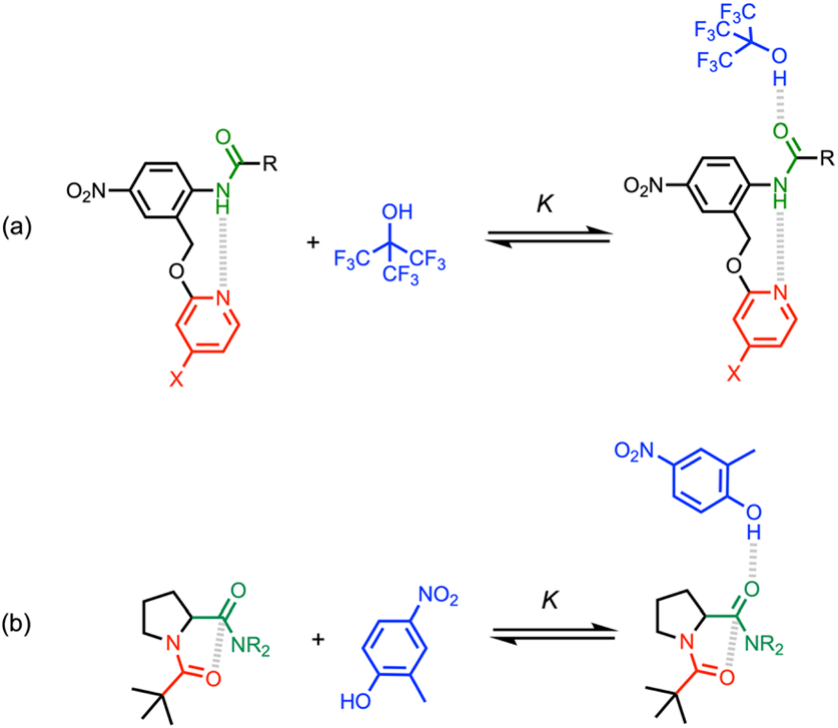
(a) Complex used previously to investigate cooperativity between two H-bonding interactions.^29^ (b) Complex used here to investigate cooperativity between an n-π* interaction and a H-bonding interaction.

We have previously measured cooperativity between H-bonding interactions with secondary amides using the system shown in Fig. 2a.^29^ Measurement of the strength of the intermolecular H-bond with perfluoro-*t*-butanol (blue) allowed quantification of the effect of the intramolecular H-bond between the pyridine moiety (red) and the amide NH on the polarity of the amide carbonyl oxygen (green). Positive cooperativity was observed: electron-donating X substituents on the pyridine ring that increase the strength of the intramolecular H-bond were found to increase the strength of the intermolecular H-bond by a factor of up to 4.

Here we apply a similar approach to investigate cooperativity between an intramolecular n-π* interaction and an intermolecular H-bond (Fig. 2b). The steric bulk of the pivaloyl group on the red amide favours the folded conformation, locking in a geometry where an intramolecular n-π* interaction is expected.^30–33^ Comparison of the H-bond acceptor properties of this compound with a series of closely-related analogues that lack the n-π* interaction allows quantification of cooperativity between the two interactions indicated in Fig. 2b. The H-bond donor used to measure the intermolecular H-bonding interactions was 2-methyl-4-nitrophenol, because it is one of the best H-bond donors available, it has sufficient solubility in non-polar solvents to allow titrations to be carried out in *n*-octane, and it has a strong UV-vis absorption band that can be used to monitor H-bond formation.

## Results and discussion

**Fig. 3:**
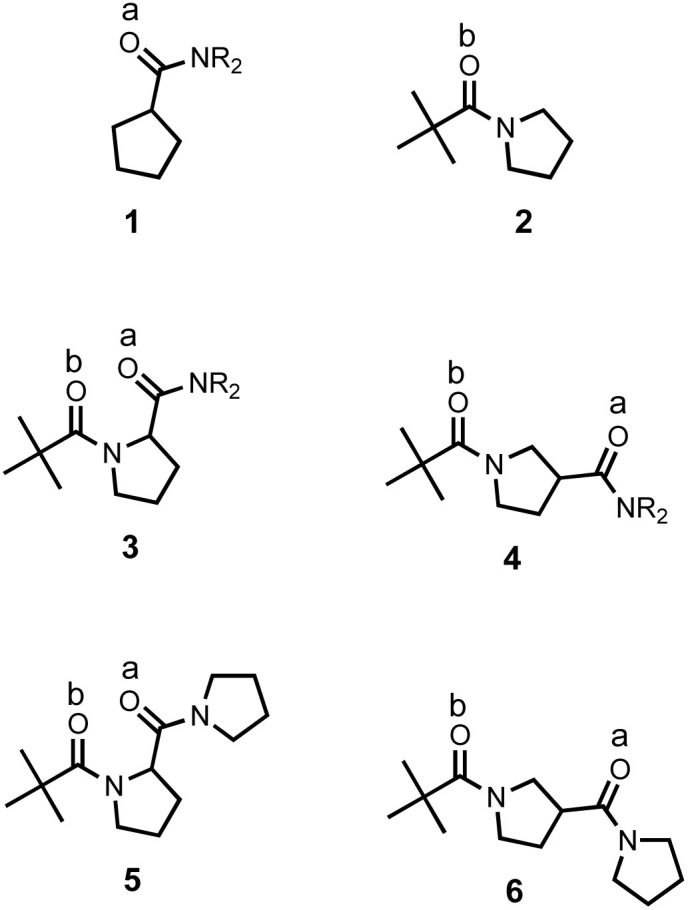
Compounds used to measure the H-bond acceptor properties of amides. The two chemically distinct amide groups are identified: the exocyclic amide groups (electron acceptors in the n-π* interactions in compounds 3 and 5) are labelled a; the pivaloyl amide groups (electron donors in the n-π* interactions in compounds 3 and 5) are labelled b. R = *n*-hexyl.

The compounds in Fig. 3 were synthesised using conventional amide coupling chemistry (see Experimental for details). The *N*-acylprolines, 3 and 5, are expected to adopt a conformation with an intramolecular n-π* interaction between the carbonyl oxygen of pivaloyl group and the carbon of the exocyclic amide.^30^ The other four compounds in Fig. 3 were used to investigate the H-bonding properties of the corresponding amide groups in molecules where there is no possibility to form an intramolecular an n-π* interaction.

### X-ray crystal structures

**Fig. 4:**
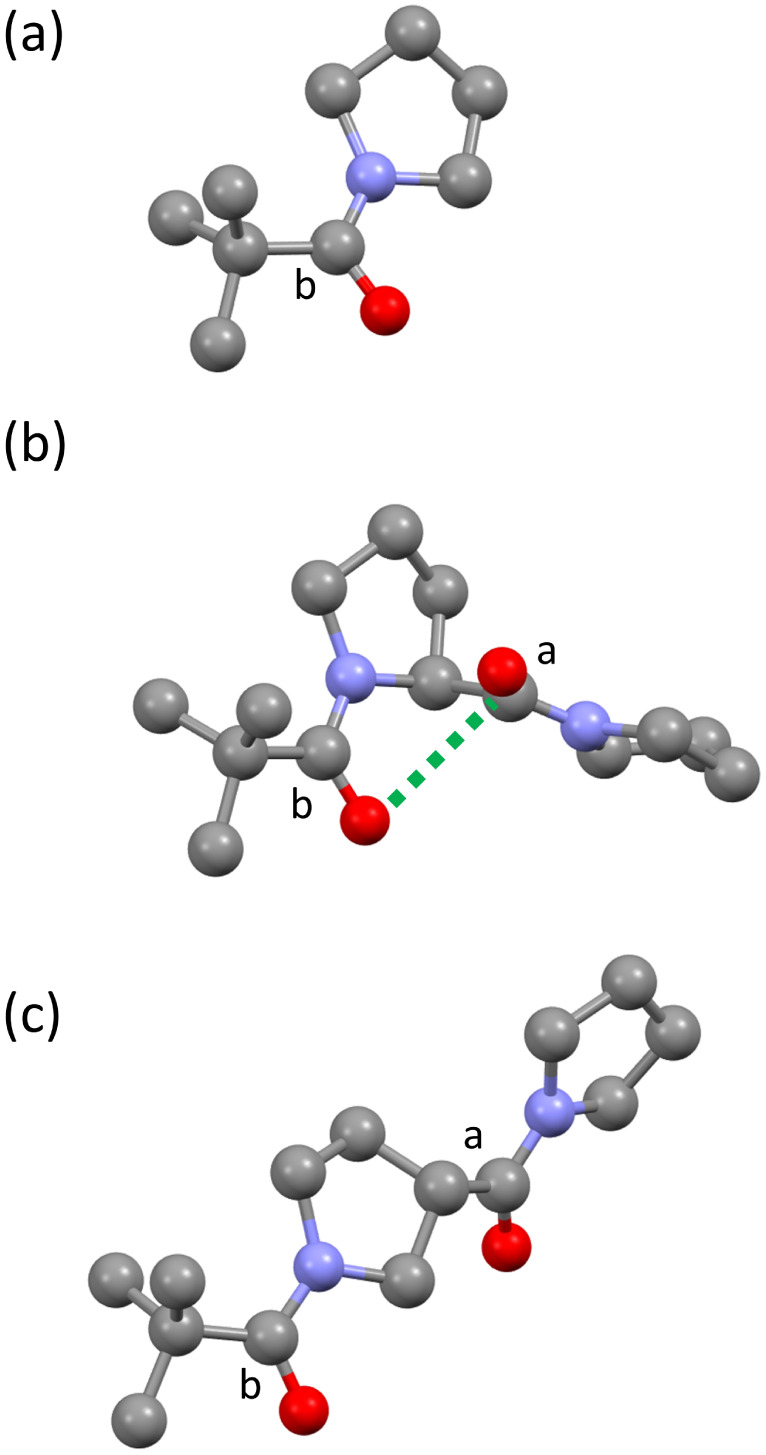
Molecular structures from X-ray crystal structures of compounds 2 (a), 5 (b) and 6 (c). The close contact between the carbon of carbonyl a and the oxygen of carbonyl b, indicated with a green dashed line in compound 5, is classified as an n-π* interaction.

Single crystals of 2, 5 and 6 suitable for X-ray diffraction were obtained by recrystallisation from *n*-hexane. In the solid state, compound 5 shows the anticipated intramolecular contact between the two amide groups (Fig. 4b). Measurement of the geometric parameters in Fig. 1 indicates that this contact is consistent with the established criteria used to assign n-π* interactions (Table 1): the distance *d* is less than the sum of the van der Waals radii, the angle *θ* falls within the reported range, and the carbonyl group labelled a shows a significant deviation away from planar sp^2^ geometry.^2^Table 1 compares the values of *Δ* and *Θ* for compound 5 with compounds 2 and 6, where there are no close contacts between any of the amide groups.

**Table 1:** Geometric parameters from Fig. 1 measured in X-ray crystal structures

Compound	Carbonyl	*d*/Å	*Θ*/deg.	*Δ*/Å	*Θ*/deg.
2	b	—	—	0.0023(15)	0.29(16)
5	a	2.935(2)	93.33(12)	0.021(2)	2.6(2)
5	b	3.117(2)	84.84(11)	0.006(2)	0.8(2)
6	a	—	—	0.006(2)	0.74(16)
6	b	—	—	0.006(2)	0.78(16)

In 2 and 6, any difference of *Δ* and *Θ* from zero is either insignificant or barely significant relative to the uncertainties. Carbonyl group a in compound 5 is clearly more pyramidal, and the degree of pyramidalization is comparable to that reported by Raines *et al.* for compounds containing n-π* interactions.^1^ There are no notable intermolecular contacts in any of the crystal structures that could influence the molecular geometry.

### UV-vis absorption titrations

**Fig. 5:**
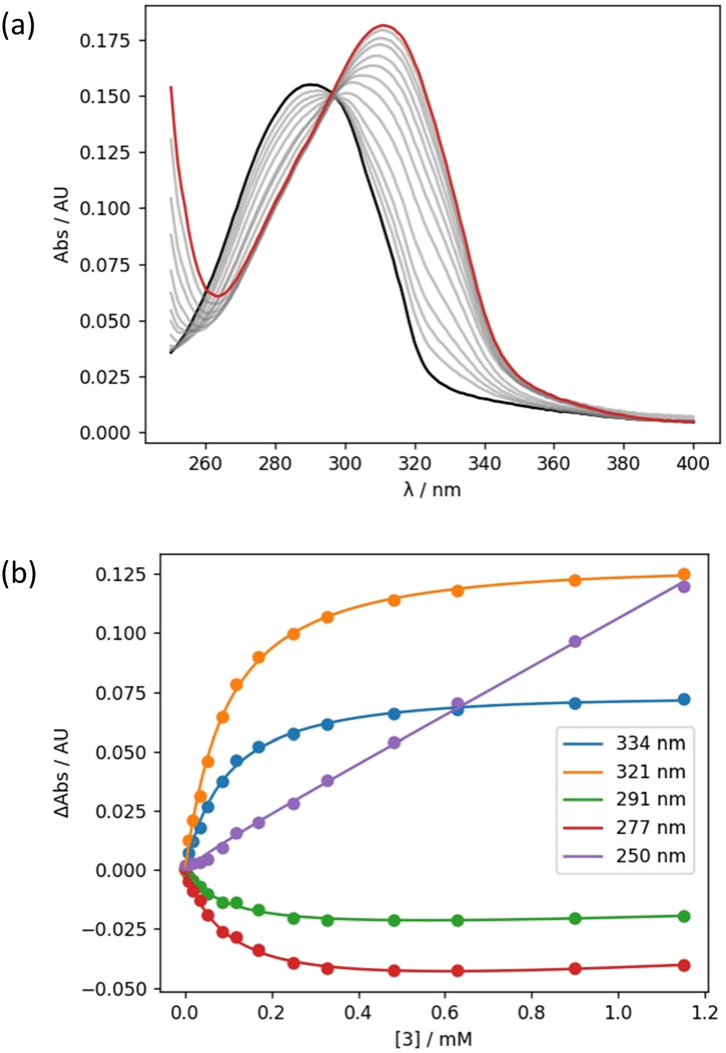
(a) UV-vis absorption spectra for the titration of 3 into 2-methyl-4-nitrophenol (0.015 mM in *n*-octane at 298 K). The spectrum of 2-methyl-4-nitrophenol and the final point of the titration are reported in black and in red, respectively. (b) Best fit of the absorbance at five different wavelengths to a 1 : 1 binding isotherm that accounts for the guest absorption (the best fit was obtained by fitting all wavelengths between 250 and 400 nm simultaneously).^34^

The interaction of compounds 1–4 with 2-methyl-4-nitrophenol was investigated using UV-vis absorption titrations. Fig. 5 shows an example of titration of compound 3 into 2-methyl-4-nitrophenol in *n*-octane solution. On addition of 3, the absorption band at 290 nm decreased and was replaced by a new band at 320 nm, which is characteristic of the formation of a H-bonded complex (Fig. 5a). In all cases, the data fit well to a 1 : 1 binding isotherm (Fig. 5b), and the resulting association constants are reported in Table 2 (see ESI† for details).^34^ Compounds 3 and 4 both have two amide groups and might therefore be expected to bind to two molecules of 2-methyl-4-nitrophenol to give 2 : 1 complexes. However, the amides were always present in a very large excess in all of the UV-vis titrations, so only 1 : 1 complexes were populated.

**Table 2:** Association constants for formation of 1 : 1 complexes with 2-methyl-4-nitrophenol measured by UV-vis absorption titrations in *n*-octane at 298 K^a^

Compound	*K*/M^−1^
1	5200 ± 300
2	5900 ± 200
3	12 000 ± 2000
4	8200 ± 800

a Errors are quoted as twice the standard error of the mean of at least three measurements (95% confidence interval).^35^

**Fig. 6:**
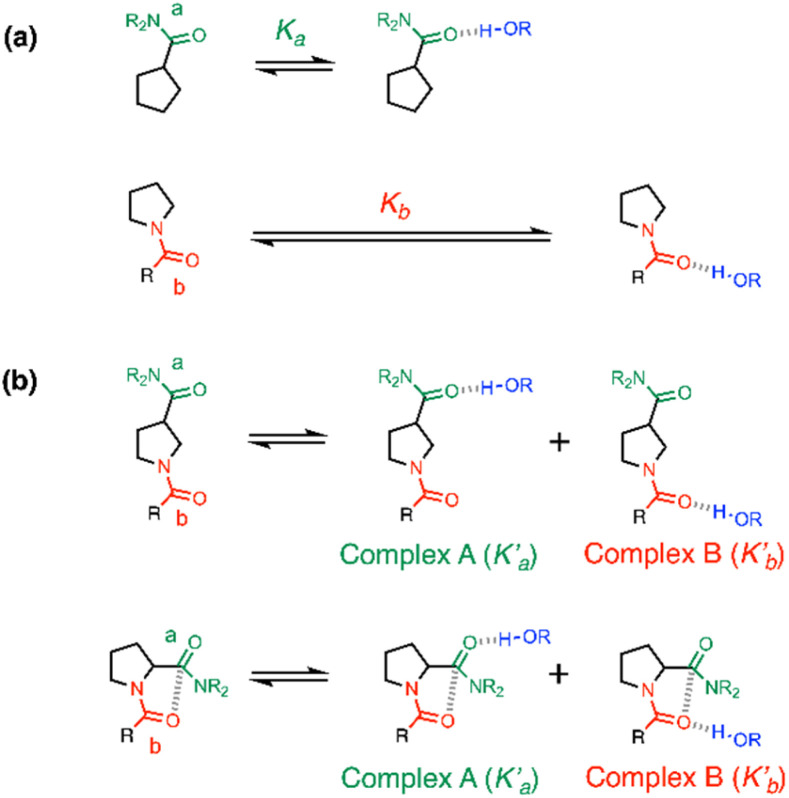
Formation of 1 : 1 complexes with a H-bond donor (blue). (a) Compounds with one amide group form a single 1 : 1 complex. (b) Compounds with two amide groups form two different 1 : 1 complexes. The macroscopic association constant measured for the 1 : 1 complex, *K*_1_, is the sum of the microscopic association constants for the two different 1 : 1 complexes. In the absence of cooperativity, the association constants, *K*_a_ and *K*_b_, (shown in part a) can be used to estimate the microscopic association constants, 
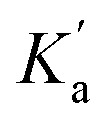
 and 
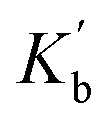
, (in part b).

The association constants measured for formation of 1 : 1 complexes with compounds 1 and 2, which have one amide group, are very similar, indicating that the two different types of carbonyl group are equally good H-bond acceptors. The association constants measured for formation of 1 : 1 complexes with compounds 3 and 4, which have two amide groups, are significantly higher. Fig. 6 illustrates the relationship between association constants for compounds that form one H-bond (Fig. 6a) or two H-bonds (Fig. 6b). Compounds with two amide groups (*i.e.*3 and 4) form two different 1 : 1 complexes with a H-bond donor, complex A and complex B.

In the UV-vis titrations, 2-methyl-4-nitrophenol was used as the host to monitor the extent of binding, so the total amount of bound host, [Host]_b_, is given by the sum of the concentrations of complex A and complex B (eqn (1)).

1 [Host]_b_ = [complex A] + [complex B]

If the microscopic association constants for formation of the two different 1 : 1 complexes are 
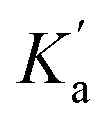
 and 
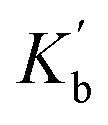
, then the concentrations of complex A and complex B can be expressed in terms of the concentrations of free host, [Host]_f_, and free diamide guest, [Guest]_f_ (eqn (2) and (3)).2
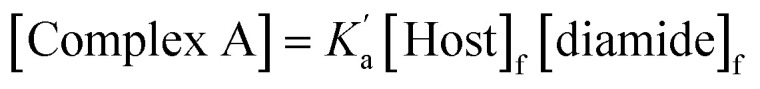
3
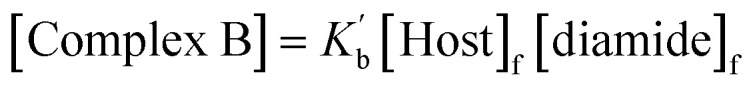


Combining eqn (1)–(3) gives eqn (4).4
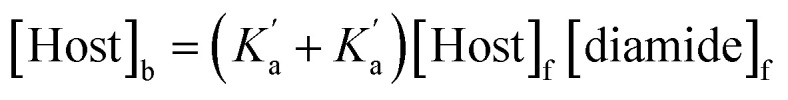


Eqn (4) shows that the macroscopic association constant measured in the UV-vis titration, *K*, is the sum of the microscopic association constants for formation of the two different 1 : 1 complexes (eqn (5)).5
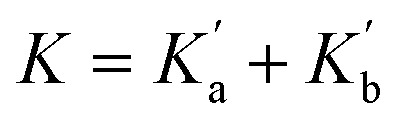


The relative populations of the two different 1 : 1 complexes can similarly be obtained by combining eqn (2), (3) and (6).6
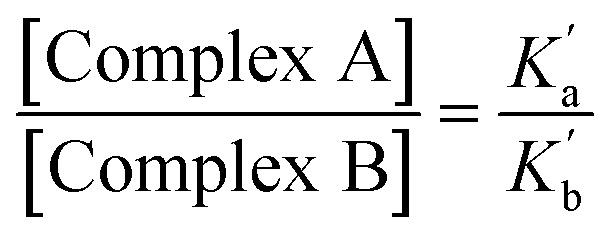


The amide groups in 3 and 4 are similar to those in 1 and 2, so in the absence of any cooperativity, the microscopic association constants for formation of Complexes A and B, 
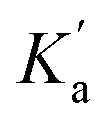
 and 
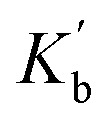
, should be similar to the corresponding association constants for the compounds that have one amide group, *K*_a_ and *K*_b_. Using the values measured for 1 and 2 as 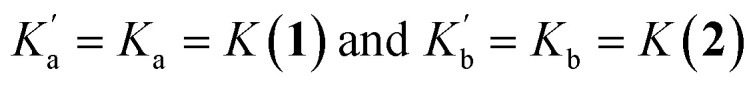 in eqn (6) indicates that in the absence of any cooperativity the H-bond donor would be bound to carbonyl a in 47% of the 1 : 1 complexes and bound to carbonyl b in the other 53%. In other words, the populations of the two different states would be similar, because the microscopic association constants are similar. Using 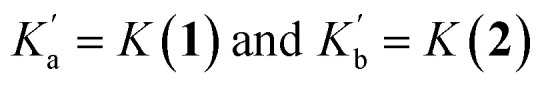 in eqn (6) gives the macroscopic association constant for formation of this mixture of 1 : 1 complexes, *K* = 11 100 ± 400 M^−1^ in the absence of any cooperativity.

Any difference between this value and the value of the association constant measured for formation of the 1 : 1 complex with compound 3 provides a measurement of cooperativity between the H-bonding interactions and the n-π* interaction. The free energy contribution associated with cooperative effects due to the presence of the n-π* interaction in compound 3 can therefore be estimated as −0.2 ± 0.4 kJ mol^−1^ using eqn (7).

7 ΔΔ*G*° = −*RT* ln{*K*(**3**)/(*K*(**1**) + *K*(**2**))}

In compound 4, the two amide groups are remote and there is no n-π* interaction, so the measurements carried out on this compound can be used to quantify the uncertainty associated with the assumption that the microscopic association constants for the amide groups are transferrable between different compounds. The difference between the association constant measured for compound 4 and the value estimated using eqn (5) corresponds to a free energy difference of +0.8 ± 0.3 kJ mol^−1^ (eqn (8)). In effect, this value corresponds to the resolution of the experiment, and any free energy differences that are smaller the 0.8 kJ mol^−1^ fall within the experimental error of the approach.

8 ΔΔ*G*° = −*RT* ln{*K*(**4**)/(*K*(**1**) + *K*(**2**))}

An alternative approach to assessing the cooperativity associated with the n-π* interaction in compound 3 is to directly compare the association constants measured for compounds 3 and 4, which both contain two amide groups. This analysis suggests that the presence of the n-π* interaction in compound 3 increases the strength of the interaction with 2-methyl-4-nitrophenol by −0.9 ± 0.5 kJ mol^−1^ (eqn (9)).

9 ΔΔ*G*° = −*RT* ln{*K*(**3**)/*K*(**4**)}

Since this experiment measures the overall effect of the n-π* interaction on the two different 1 : 1 complexes shown in Fig. 6b, it is not possible to distinguish whether any cooperativity measured is due to changes in 
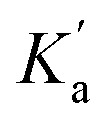
 or 
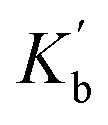
. It is instructive to consider the extreme case of 
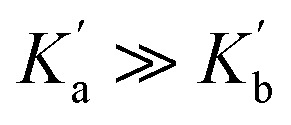
. If the n-π* interaction were to completely inhibit formation of a H-bond with carbonyl b, the association constant measured for formation of the 1 : 1 complex with compound 3 would correspond to 
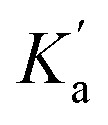
 (see eqn (5)), and the increase in the stability of the H-bond formed with carbonyl a would be given by eqn (10).

10 ΔΔ*G*° = −*RT* ln{*K*(**3**)/*K*(**1**)}

Eqn (10) places a theoretical upper bound on the magnitude of the cooperativity between the H-bond formed with carbonyl a and the n-π* interaction in the complex formed with compound 3 (−1.8 ± 0.4 kJ mol^−1^). However, eqn (10) only applies if Complex B were not populated at all (see eqn (6)), and the NMR experiments below show that this is not the case. The two different 1 : 1 complexes formed by compound 3 are populated to similar extents, which indicates that 
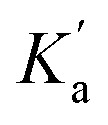
 and 
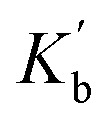
 are relatively close in value. We conclude therefore that the presence of the n-π* interaction in compound 3 increases the stability of the 1 : 1 complex formed with 2-methyl-4-nitrophenol by between −0.2 and −0.9 kJ mol^−1^, but note that this free energy difference is comparable to the uncertainty of the experiment (0.8 kJ mol^−1^).

### ^13^C NMR titrations

In order to obtain more information about the preferred site of interaction of compounds 1–4 with H-bond donors, ^13^C NMR spectroscopy was used to monitor changes in the chemical shifts of the signals due to the carbonyl groups on interaction with a H-bond donor. For these experiments, a more soluble H-bond donor, perfluoro-*tert*-butanol (PFTB), was used in place of 2-methyl-4-nitrophenol, as much higher concentrations were required. The amides were used as hosts at concentrations of 50–100 mM, but ^1^H NMR dilution experiments showed no evidence of self-association for any of compounds 1–4 in *n*-octane (see ESI†).

**Fig. 7:**
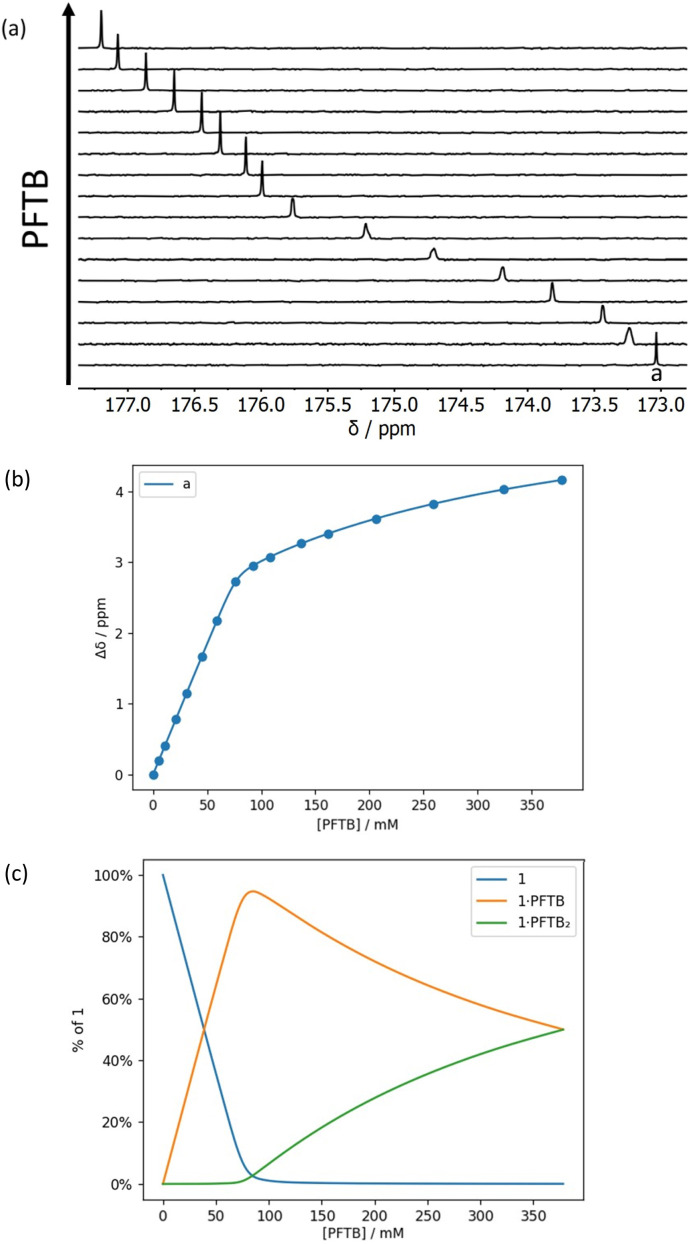
(a) Partial 176 MHz ^13^C NMR spectra for titration of perfluoro-*tert*-butanol into 1 (69 mM) in *n*-octane at 298 K. (b) Best fit of the NMR data to a 1 : 2 binding isotherm (*K*_1_ > 10^3^ M^−1^ and *K*_2_ = 4 M^−1^). (c) Speciation as a function of PFTB concentration.^34^

**Fig. 8:**
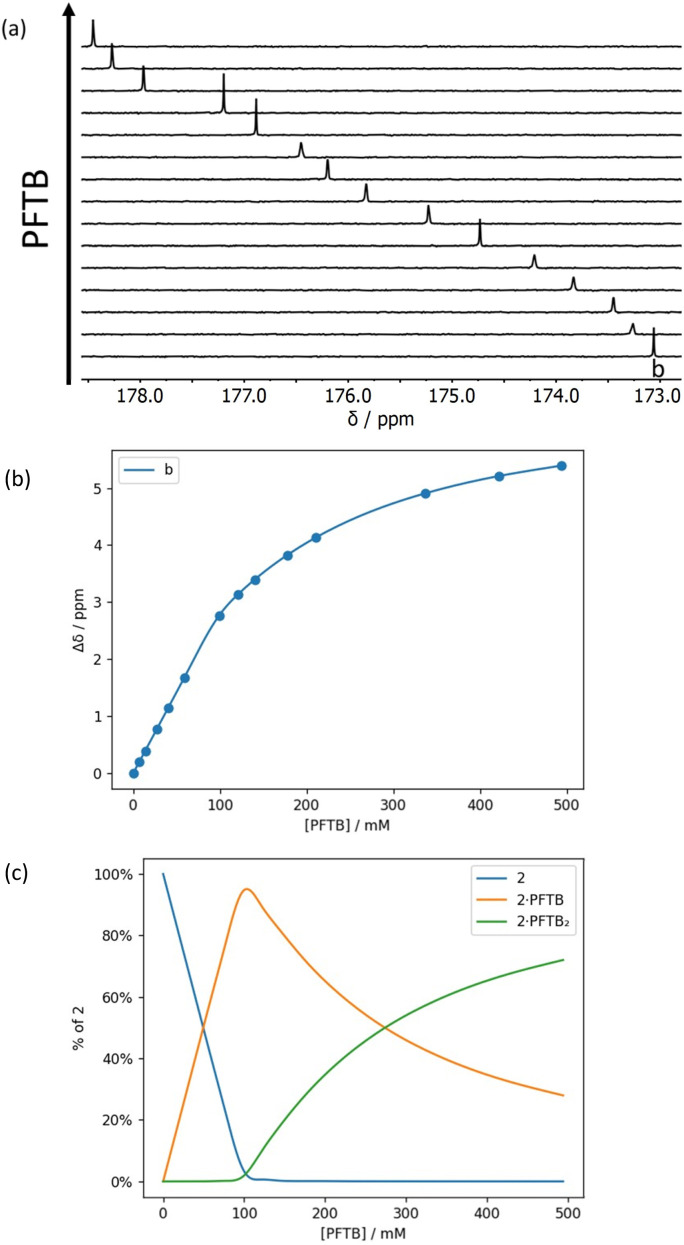
(a) Partial 176 MHz ^13^C NMR spectra for titration of perfluoro-*tert*-butanol into 2 (92 mM) in *n*-octane at 298 K. (b) Best fit of the NMR data to a 1 : 2 binding isotherm (*K*_1_ > 10^3^ M^−1^ and *K*_2_ = 7 M^−1^). (c) Speciation as a function of PFTB concentration.^34^

Fig. 7 and 8 show the results for titration of PFTB into compounds 1 and 2. The ^13^C NMR chemical shift of the signal due to the carbonyl group increased linearly with guest concentration, until one equivalent of PFTB had been added, at which point there was a sharp kink followed by a more gradual increase in chemical shift. This behaviour is characteristic of a titration carried out in the tight binding limit, with formation of a high affinity interaction followed by a second low affinity interaction. PFTB (*α* = 4.9) is a better H-bond donor than 2-methyl-4-nitrophenol (*α* = 4.7),^36,37^ so tight binding for formation of the 1 : 1 complex is consistent with the association constants measured in the UV-vis absorption titrations. Although the concentrations used in the NMR titrations were too high for accurate determination of the association constants for formation of the 1 : 1 complexes, these experiments do provide a reliable method for determining the stoichiometry and the limiting complexation-induced changes in chemical shift. The titration data fit well to a 1 : 2 binding isotherm (Fig. 7 and 8), and the association constants for binding a second PFTB to the same carbonyl group are very low (4 M^−1^ for 1 and 7 M^−1^ for 2). The limiting complexation-induced changes in ^13^C NMR chemical shift for formation of the 1 : 1 complexes are +2.9 and +2.8 ppm (see ESI† for details).

**Fig. 9:**
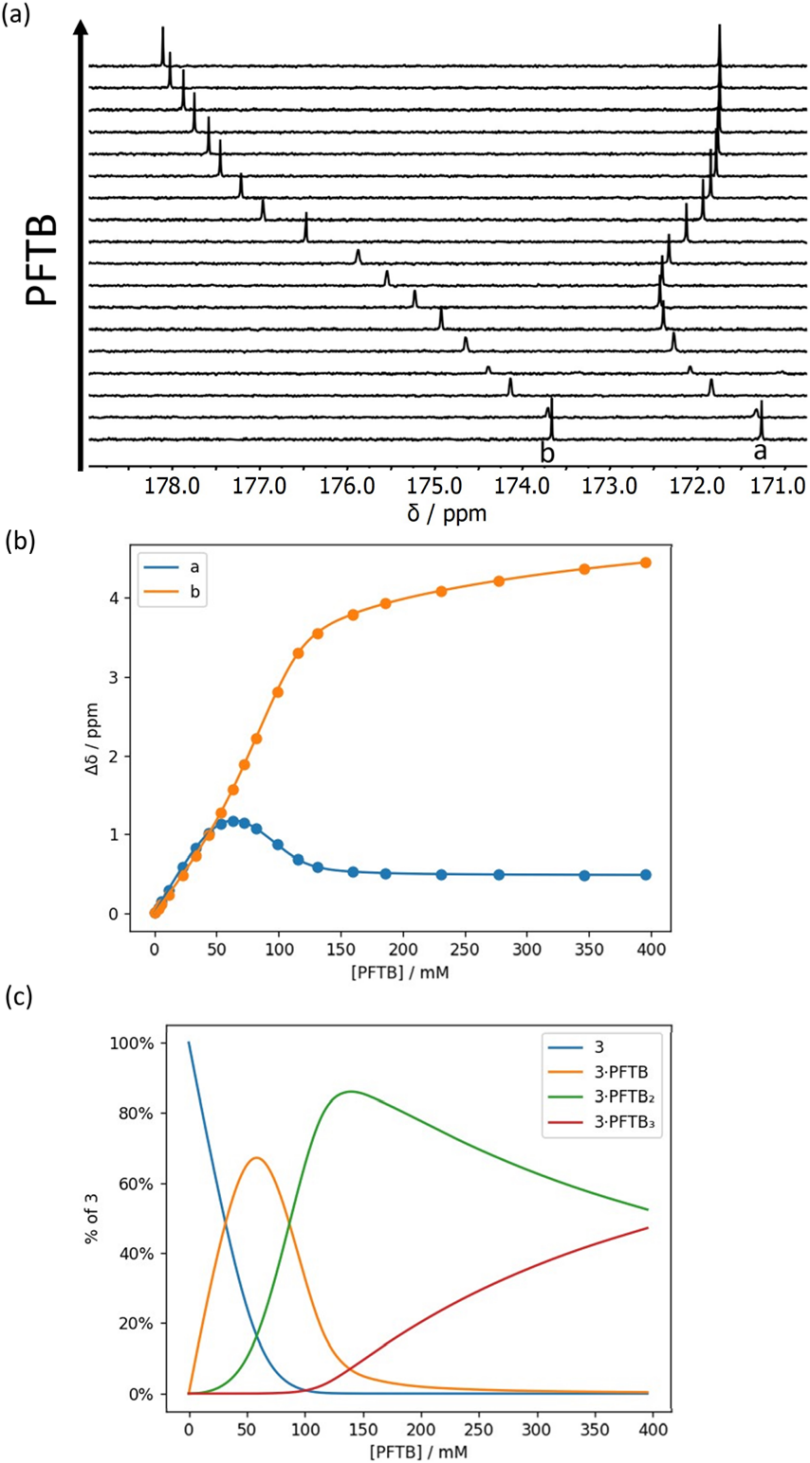
(a) Partial 176 MHz ^13^C NMR spectra for titration of PFTB into 3 (52 mM) in *n*-octane at 298 K. (b) Best fit of the NMR data to a 1 : 3 binding isotherm (*K*_1_ and *K*_2_ > 10^3^ M^−1^ and *K*_3_ = 4 M^−1^). (c) Speciation as a function of PFTB concentration.^34^

For compound 3, three distinct phases were observed in the titration with PFTB, with relatively sharp changes after addition of approximately one and then two equivalents of PFTB (Fig. 9). In this case, the data fit well to a 1 : 3 binding isotherm (Fig. 9b) with two high affinity binding interactions and a third low affinity interaction (*K*_3_ = 4 M^−1^). This result suggests that the first two binding events correspond to binding of one PFTB to each of the two carbonyl groups, and the third binding event corresponds to the interaction of a second PFTB with a bound carbonyl (*cf. K*_2_ for compounds 1 and 2). One might expect a 1 : 4 complex in which both carbonyl groups bind a second equivalent of PFTB, but the 1 : 3 complex only reaches a population of 40%, so the fourth binding event would be difficult to distinguish (Fig. 9c).

**Fig. 10:**
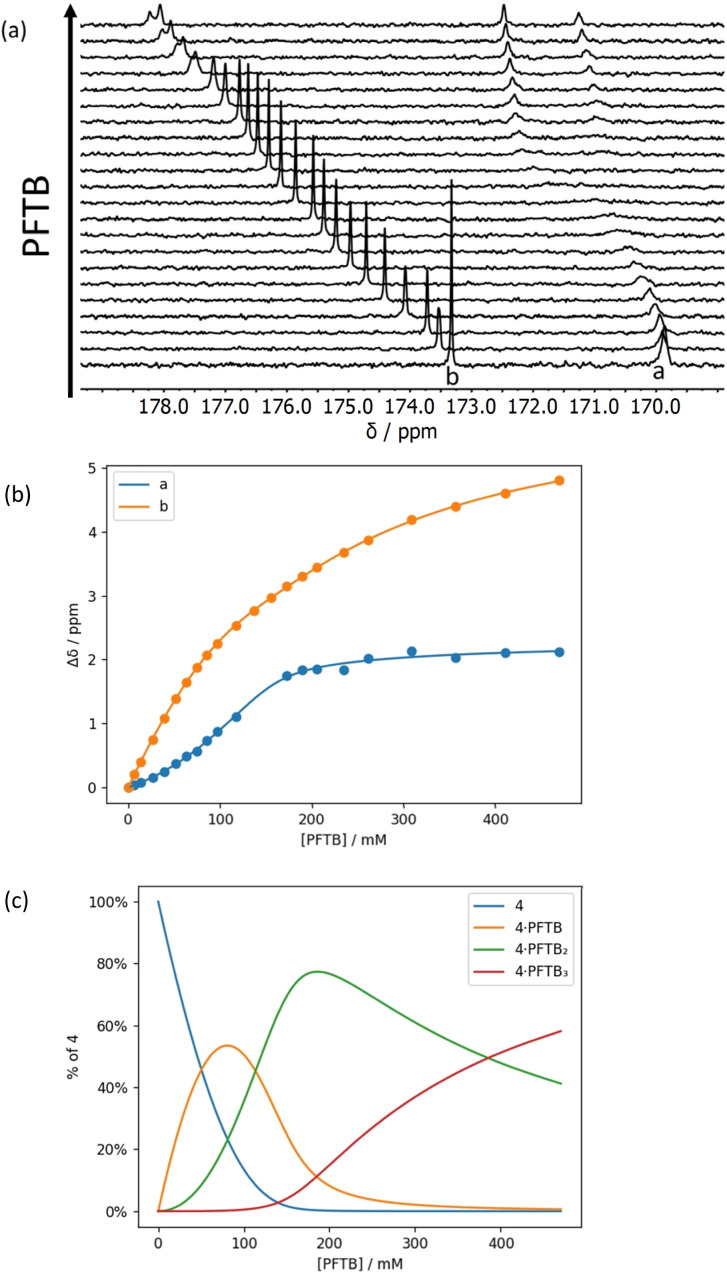
(a) Partial 176 MHz ^13^C NMR spectra for titration of perfluoro-*tert*-butanol into 4 (90 mM) in *n*-octane at 298 K. (b) Best fit of the NMR data to a 1 : 3 binding isotherm (*K*_1_ and *K*_2_ > 10^3^ M^−1^ and *K*_3_ = 5 M^−1^). (c) Speciation as a function of PFTB concentration.^34^

For compound 4 in the presence of excess PFTB, both ^13^C NMR signals split into two signals of equal intensity in slow exchange (Fig. 10a). The splitting may be related to the presence of two almost degenerate conformers due to slow rotation around the pivolyl amide C–N bond. Similar behaviour is not observed for compound 3, because the increased steric hindrance in this compound precludes population of one of the isomers. The titration data for 4 were analysed by converting the chemical shifts of the two slow exchange signals to the corresponding fast exchange chemical shift, *i.e.* the average of the two values. The resulting data fit well to a 1 : 3 binding isotherm (Fig. 10b) with two high affinity binding interactions and a third low affinity interaction (*K*_3_ = 5 M^−1^). As for compound 3, the first two binding events correspond to binding of one PFTB to each of the two carbonyl groups, and the third binding event corresponds the interaction of a second PFTB with a bound carbonyl.

**Fig. 11:**
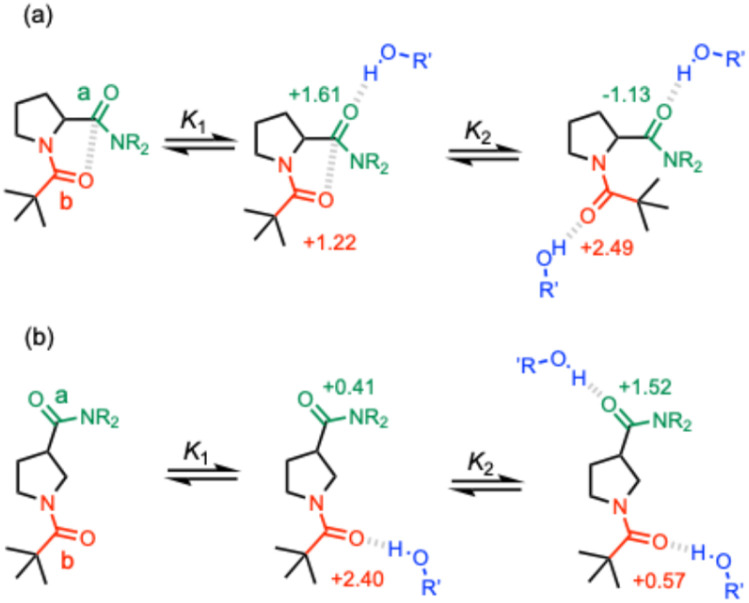
Limiting stepwise complexation-induced changes in ^13^C NMR chemical shift for the carbonyl groups a (green) and b (red) of compounds 3 (a) 4 (b) in the 1 : 1 and 1 : 2 complexes formed with PFTB, and corresponding structures of the major species present in solution (R = *n*-hexyl, R' = perfluoro-*t*-butyl).

The limiting complexation-induced changes in ^13^C NMR chemical shift observed for the two carbonyl groups in compounds 3 and 4 were used to infer the preferred sites of interaction with PFTB. The stepwise changes in chemical shift for formation of the 1 : 1 complex and then conversion of the 1 : 1 complex into the 1 : 2 complex are shown in Fig. 11. For compound 4, binding of the first PFTB is associated with a large increase in chemical shift for carbonyl b (+2.4 ppm) and a small increase for carbonyl a (+0.4 ppm), which suggests that PFTB binds to both sites in the 1 : 1 complex, but there is a marked preference for carbonyl b (Fig. 11b). As a consequence, binding of the second PFTB occurs mainly at carbonyl a, and the pattern of chemical changes is reversed.

For compound 3, the pattern is quite different (Fig. 11a). Binding of the first PFTB is associated with large increases in chemical shift for both carbonyl carbons (+1.6 and +1.2 ppm) with a preference for carbonyl a. Binding of the second PFTB leads to a large increase in the chemical shift of carbonyl b (+2.5 ppm) suggesting that this is the site of interaction with the second PFTB. Binding of the second PFTB also leads to a large decrease in chemical shift for carbonyl a (−1.1 ppm), which suggests a significant change in environment, although density functional theory (DFT) calculations show no significant conformational changes in the 1 : 2 complex (see ESI†).

The NMR experiments therefore indicate that the association constants measured for the 1 : 1 complexes in the UV/vis absorption titrations involve interactions with both carbonyl groups in both compounds 3 and 4, but the major site of interaction in compound 3 is carbonyl a, the electron acceptor in the n-π* interaction, and the major site of interaction in compound 4 is carbonyl b. Although these observations might be considered evidence for an increase in the polarity of carbonyl a in compound 3 compared with compound 4, where there is no possibility to form an n-π* interaction, there are additional steric factors that also favour the binding of the first equivalent of PFTB to carbonyl a in compound 3.

## Conclusion

The cooperativity between H-bonding and n-π* interactions in amide groups was experimentally quantified by comparing the H-bonding properties of amide groups that are locked in a conformation consistent with formation of an intramolecular n-π* interaction with analogues that lack this intramolecular contact. The presence of the intramolecular n-π* interaction was confirmed by X-ray crystallography, and the effects on the formation of intermolecular H-bonds was investigated using UV-vis absorption and ^13^C NMR titrations. The magnitudes of the complexation-induced changes in ^13^C NMR chemical shift associated with formation of the 1 : 1 complex with perfluoro-*t*-butanol were used to infer the preferred site of interaction with a H-bond donor.

The association constants measured for formation of 1 : 1 complexes with 2-methyl-4-nitrophenol suggest that the intramolecular n-π* interaction increases the strength of the intermolecular H-bond by between 0.2 and 0.9 kJ mol^−1^, but these values fall within the uncertainty of the experiment. We conclude that any cooperative effects that operate in amide groups involved in n-π* interactions are too small to significantly affect the thermodynamic stability of simple complexes involving single points of interaction. The behaviour is quite different from the cooperative effects that have been measured for amide groups involved in two H-bonding interactions, where increases in thermodynamic stability of up to 6 kJ mol^−1^ have been observed.^29,38^ The results suggest that major contributions to the thermodynamic stability of folded proteins due to cooperativity associated with n-π* interactions are unlikely.

## Data availability

All supporting data is provided in the ESI.†

## Author contributions

The manuscript was written through contributions of all authors.

## Conflicts of interest

There are no conflicts to declare.

## Supplementary Material

SC-016-D4SC08331H-s001

SC-016-D4SC08331H-s002
