# Supplementary material for: Quantification of the effects of n-π* interactions on the H-bonding properties of amide groups
Source: Chem Sci. 2025 Apr 3;16(18):7894–901. doi: 10.1039/d4sc08331h (PMC11966642; doi:10.1039/d4sc08331h)
Supplement: SC-016-D4SC08331H-s001 [file SC-016-D4SC08331H-s001.pdf]

Supplementary Information (SI) for Chemical Science.
This journal is © The Royal Society of Chemistry 2025

Quantification of the effects of n-π* interactions on the H-bonding properties of amide groups.

Fergal E. Hanna, Andrew D. Bond and Christopher A. Hunter*

Yusuf Hamied Department of Chemistry, University of Cambridge, Lensfield Road, Cambridge CB2 1EW, UK.

Email: herchelsmith.orgchem@ch.cam.ac.uk

Supplementary Information

Table of Contents

Materials and methods S2

Synthesis and characterisation S3

UV-vis Absorption and NMR Titrations S22

^{13}C NMR Titration Shift Changes S39

Single-Crystal X-Ray Diffraction S40

***Ab initio* calculations** S43

References S45

Materials and methods

All reagents were purchased from commercial sources (Sigma Aldrich UK, Acros, Tokyo Chemical Industry, Alfa Aesar, Manchester Organics and FluoroChem) and were used as received without any further purification unless stated. Dry solvents were obtained by means of a Grubbs solvent purification system.

Flash chromatography was done with an automated system (Combiflash Companion) using pre-packed cartridges of silica (50 µm PuriFlash® column) or basic alumina (45 µm PuriFlash® column)

The LC-MS analysis of samples was performed using Waters Acquity H-class UPLC coupled with a single quadrupole Waters SQD2. ACQUITY UPLC CSH C18 Column, 130 Å, 1.7 µm, 2.1 mm X 50 mm was used as the UPLC column for all samples. The conditions of the UPLC method are as follows: Solvent A: Water +0.1% Formic acid; Solvent B: Acetonitrile +0.1% Formic acid; Gradient of 0-2 minutes 5% - 100%B + 1 minute 100% B with re-equilibration time of 2 minutes. Flow rate: 0.6 ml/min; column temperature of 40°C; injection volume of 2 µL. The signal was monitored with MS-ES+, MS-ES-, and UV-vis absorption at 254 nm or at 290 nm.

^{1}H-NMR and ^{13}C-NMR were recorded on either a 400 MHz or 500 MHz Bruker spectrometer. The reference values used for the chemical shifts of the various spectra are reported in the literature.[1] The splitting pattern is indicated with the following abbreviations: s for singlet, d for doublet, t for triplet, q for quartet, p for pentet and m for multiplet.

FT-IR spectra were collected with a Bruker ALPHA FT-IR Spectrometer.

UV-vis spectra were recorded with an Agilent UV-vis Cary 60 spectrophotometer.

Synthesis and characterisation

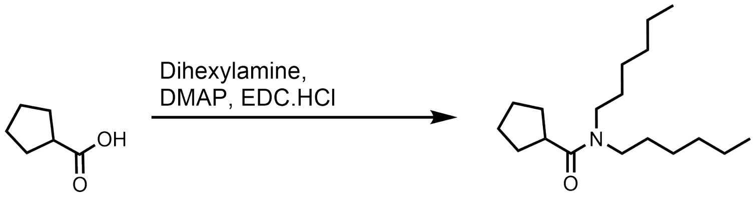

1
45%

Scheme S.1 – Synthesis of compound **1**

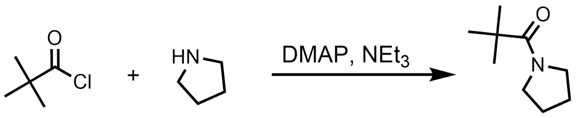

2
70%

Scheme S.2 - Synthesis of compound **2**

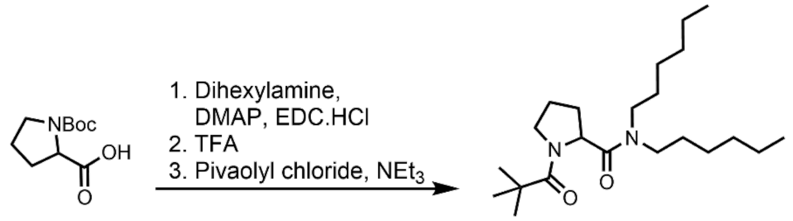

3
39%

Scheme S.3 – Synthesis of compound **3**

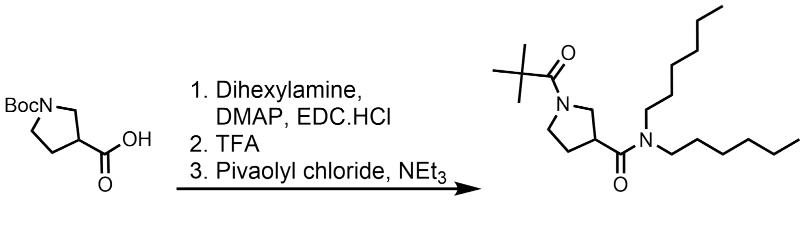

4
46%

Scheme S.4 – Synthesis of compound **4**

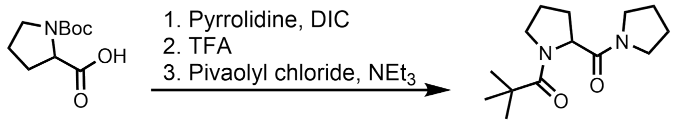

5
44%

Scheme S.5 – Synthesis of compound **5**

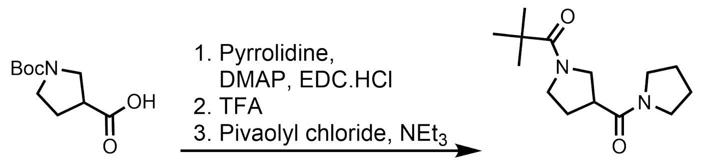

6
20%

Scheme S.6 – Synthesis of compound **6**

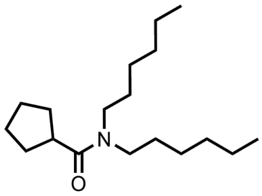

1

N,N-dihexylcyclopentanecarboxamide, **1**. Cyclopentyl carboxylic acid (0.20 mL, 1.85 mmol), EDC.HCl (0.496 g, 2.59 mmol) and DMAP (0.541 g, 4.82 mmol) were dissolved in dry DCM (10 mL) under N_2. Dihexylamine (0.50 mL, 2.14 mmol) was added and the reaction stirred for 3 days. The reaction mixture was diluted in Et_2O (50 mL), then washed with water (2 x 25 mL), saturated $NaHCO_3$ (25 mL) and brine (25 mL). The organic layer was dried with $MgSO_4$ and the solvent removed under reduced pressure. The crude mixture was purified by flash chromatography (petroleum ether/EtOAc on SiO_2) to yield the desired product as a clear oil (0.235 g, 0.835 mmol, 45%).

ν_{max} (film) cm^{-1} : 2954 (C-H), 2926 (C-H), 2858 (C-H), 1638 (C=O), 1454, 1424, 137, 1295, 1247, 1191, 1134, 727

^1H NMR (400 MHz, $CDCl_3$) δ_H ppm: 3.34 – 3.18 (m, 4H), 2.89 – 2.75 (m, 1H), 1.83 – 1.70 (m, 6H), 1.59 – 1.44 (m, 6H), 1.35 – 1.22 (m, 12H), 0.93 – 0.83 (m, 6H).

$^{13}C\{^1H\}$ NMR (101 MHz, $CDCl_3$) δ_C ppm: 176.1, 47.9, 46.2, 41.2, 31.8, 31.7, 30.9, 29.7, 27.9, 26.9, 26.8, 26.4, 22.8, 22.8, 14.2, 14.2.

HRMS: calc for $C_{18}H_{36}NO^+$ $[M+H]^+$: 282.2797, found: 282.2787

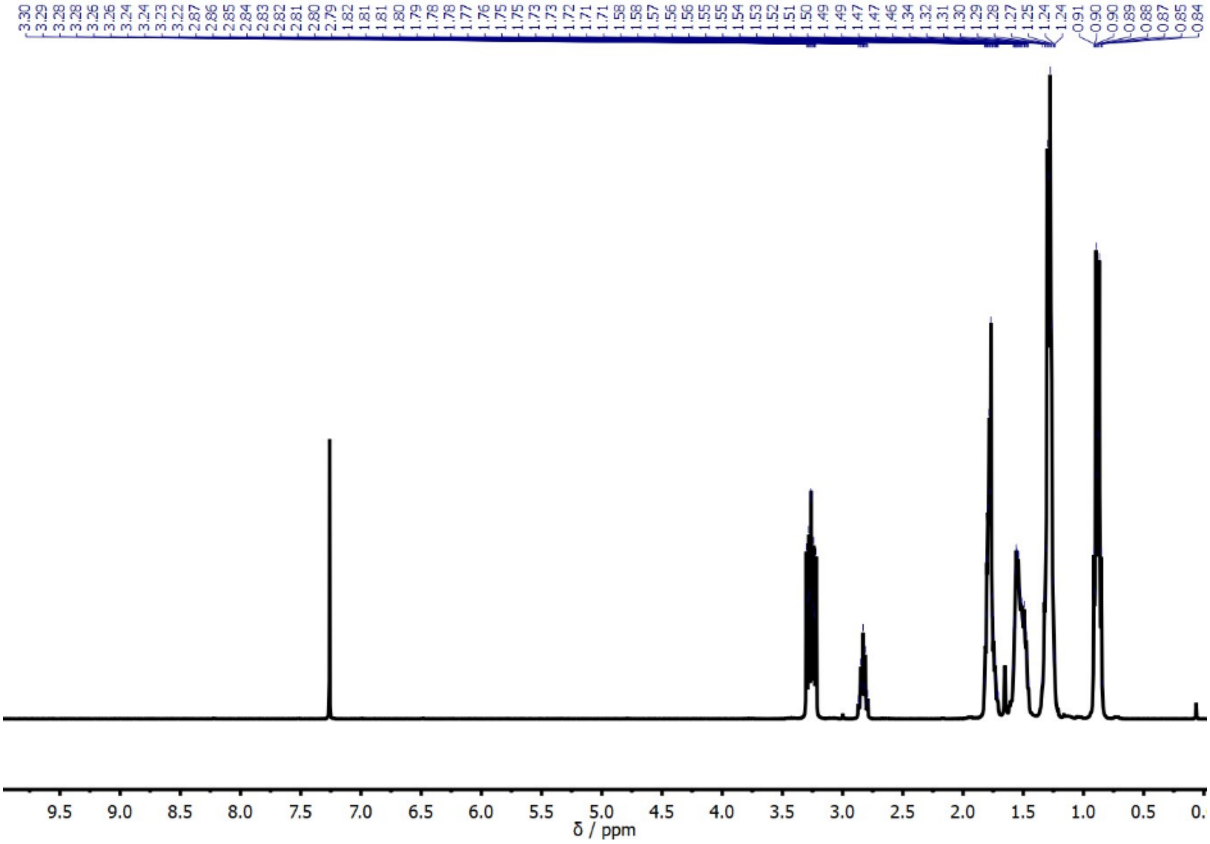

Figure S.1 – 400 MHz ^1H NMR spectrum of **1** in $CDCl_3$ (δ 0 – 10 ppm).

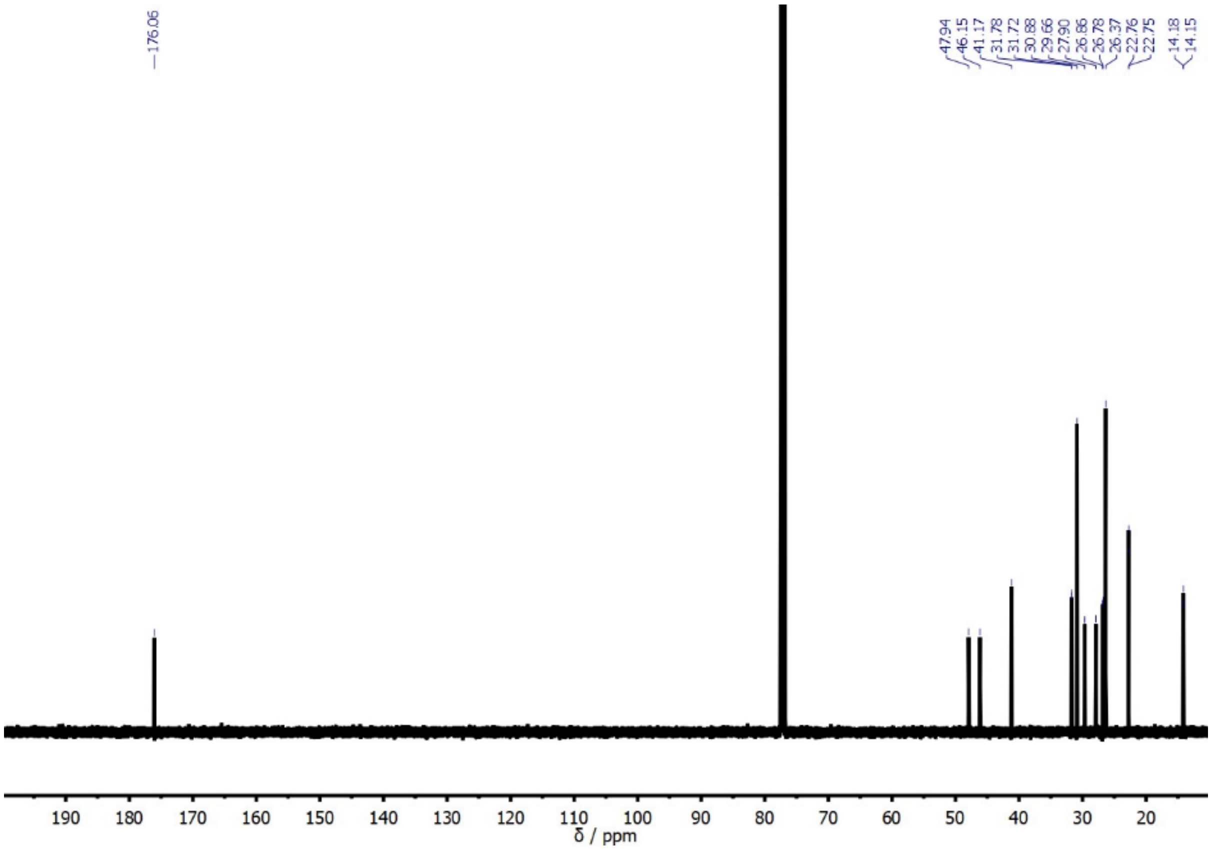

Figure S.2 – 101 MHz ^{13}C NMR spectrum of **1** in $CDCl_3$ (δ 0 – 200 ppm).

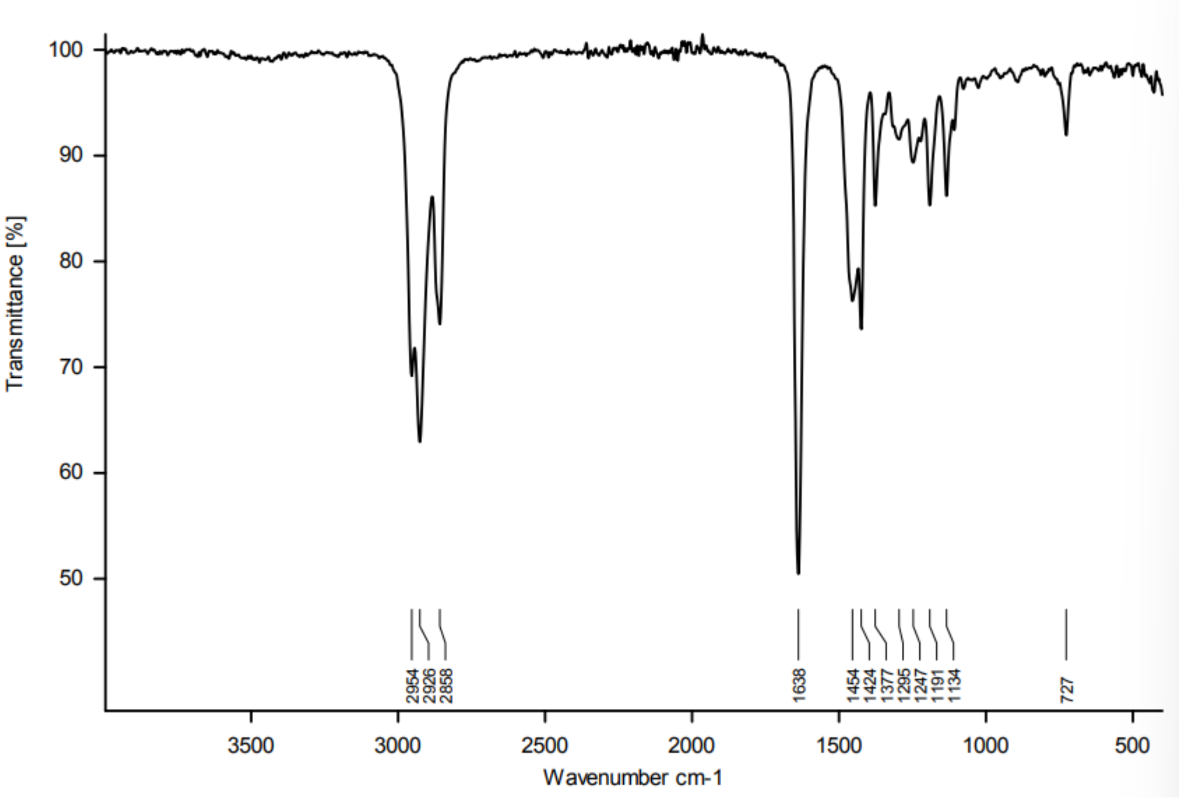

Figure S.3 – FT-IR spectrum of **1**

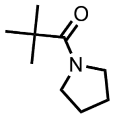

2

2,2-dimethyl-1-(pyrrolidin-1-yl)propan-1-one, **2**. Pyrrolidine (1.0 mml, 12 mmol) and DMAP (75.4 mg, 0.617 mmol) were dissolved in dry DCM under N_2. Triethylamine (4.0 mL, 29 mmol) and pivaloyl chloride (1.5 mL, 12 mmol) were added. The reaction mixture was stirred for 3 hours and diluted with EtOAc (50 mL) then washed with water (25 mL), 1M HCl (25 mL), saturated $NaHCO_3$ (25 mL) and brine (25 mL). The organic layer was dried with $MgSO_4$ and the solvent removed under reduced pressure. The crude residue was purified by flash column chromatography (PE/EtOAc on SiO_2) to yield the desired product as a white solid (1.30 g, 8.38 mmol, 70%).

ν_{max} (film) cm^{-1} : 2968 (C-H), 2875 (C-H), 1612 (C=O), 1479, 1407, 1382, 1363, 1339, 1214, 1184, 1167

^{1}H NMR (400 MHz, $CDCl_3$) δ_H ppm: 3.52 (s, 4H), 1.84 (s, 4H), 1.24 (s, 9H).

$^{13}C\{^{1}H\}$ NMR (101 MHz, $CDCl_3$) δ_C ppm: 176.5, 48.0, 39.1, 27.6.

HRMS: calc for $C_9H_{18}NO$ $[M+H]^+$: 156.1388, found: 156.1387

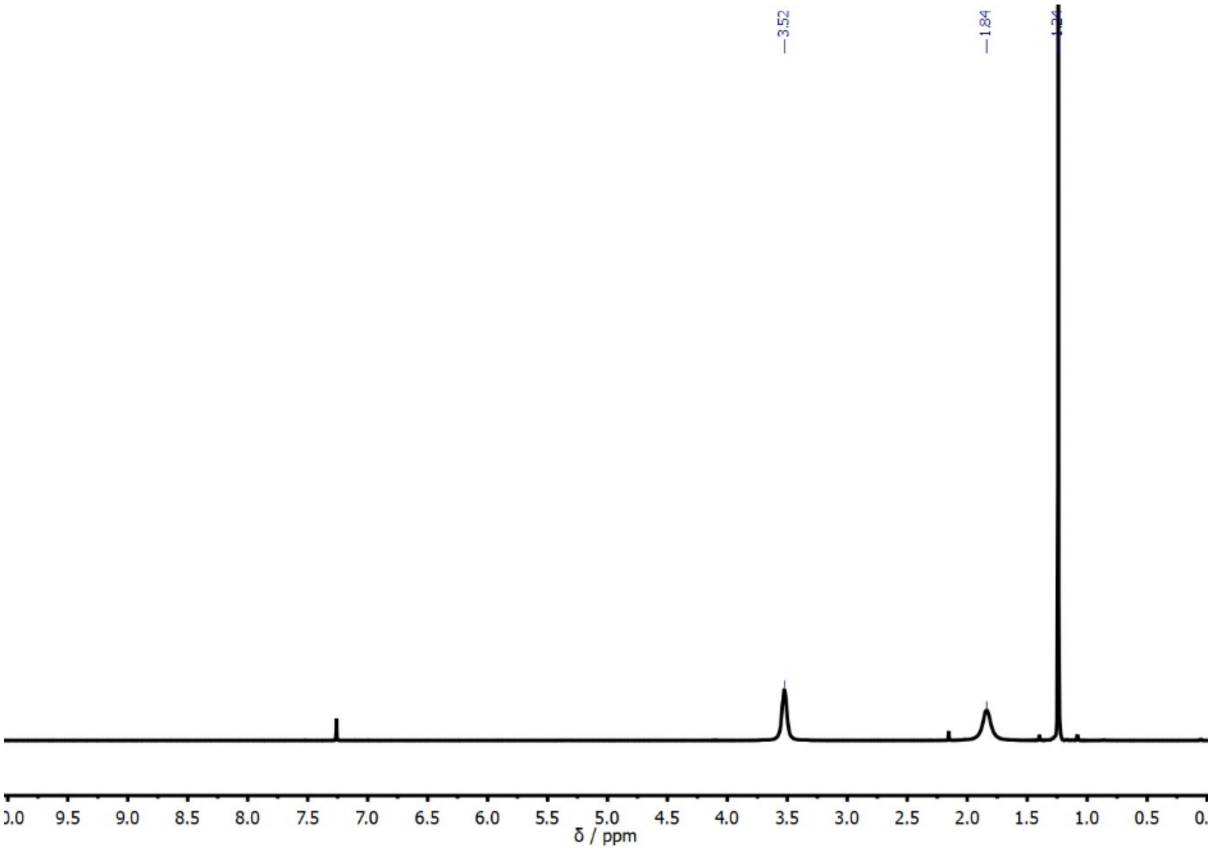

Figure S.4 – 400 MHz ^{1}H NMR spectrum of **2** in $CDCl_3$ (δ 0 – 10 ppm).

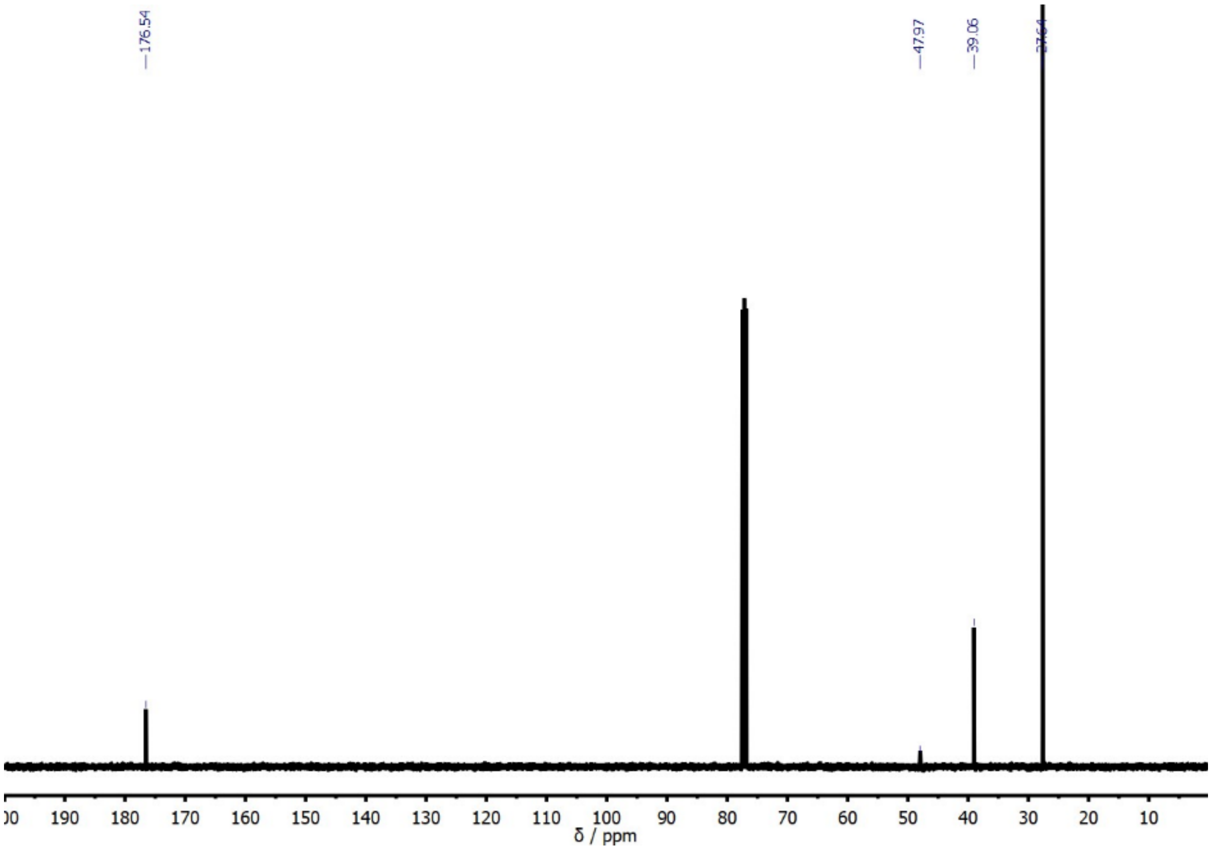

Figure S.5 – 101 MHz ^{13}C NMR spectrum of **2** in $CDCl_3$ (δ 0 – 200 ppm).

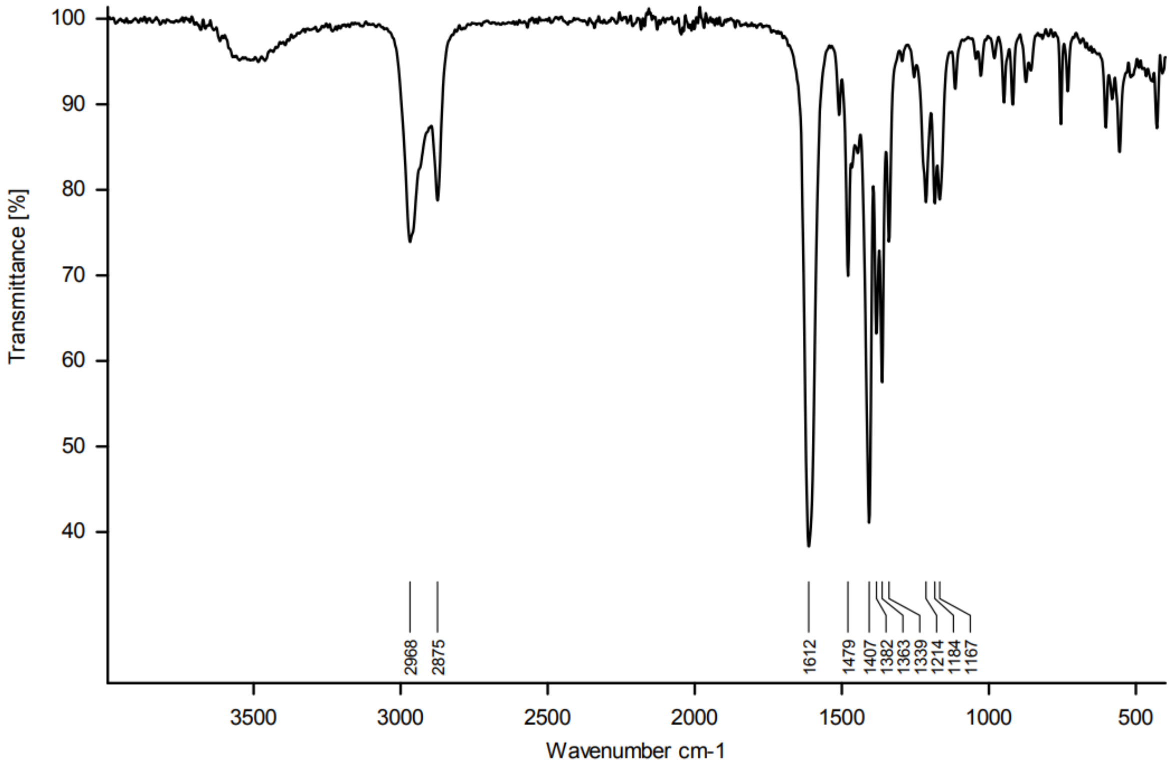

Figure S.6 – FT-IR spectrum of **2**

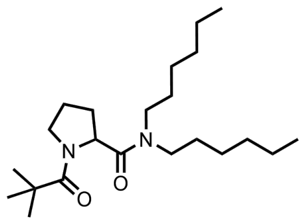

3

N,N-bis(2-ethylhexyl)-1-pivaloylpyrrolidine-2-carboxamide, **3**. *N*-boc-L-proline (0.328 g, 1.53 mmol), EDC.HCl (0.598 g, 3.12 mmol) and DMAP (0.593 g, 5.29 mmol) were dissolved in dry DCM (10 mL) under N_2. Dihexylamine (0.40 mL, 1.72 mmol) was added and the reaction stirred for 3 days. The reaction mixture was diluted in Et_2O (50 mL), then washed with water (2 x 25 mL), saturated $NaHCO_3$ (25 mL) and brine (25 mL). The organic layer was dried with $MgSO_4$ and the solvent removed under reduced pressure. The residue was dissolved in dry DCM (5 mL) under N_2 and trifluoroacetic acid (5 mL) was added. The reaction was stirred for 45 minutes and the volatiles then removed under reduced pressure. The resulting oil was dissolved in THF (25 mL) and then evaporated to dryness, this was repeated 2 more times. The resulting residue was dissolved in dry DCM (10 mL) under N_2 and triethyl amine (2.0 mL, 14 mmol) was added. Then pivaloyl chloride (0.50 mL, 4.1 mmol) was added and the reaction stirred overnight. The reaction mixture was diluted with EtOAc (50 mL) and washed with 1 M HCl (25 mL), saturated $NaHCO_3$ solution (25 mL), water (25 mL) and brine (25 mL). The organic layer was dried with $MgSO_4$ and the solvent removed under reduced pressure. The crude mixture was purified by flash chromatography (petroleum ether/EtOAc on SiO_2) to yield the desired product as a clear oil (0.217 g, 0.592 mmol, 39%)

ν_{max} (film) cm^{-1} : 2926 (C-H), 2858 (C-H), 1651 (C=O), 1622 (C=O), 1405, 1363, 1180, 1140

^{1}H NMR (400 MHz, $CDCl_3$) δ_H ppm: 4.79 (dd, J = 8.5, 4.5 Hz, 1H), 3.90 – 3.69 (m, 2H), 3.55 – 3.36 (m, 2H), 3.25 – 3.15 (m, 1H), 3.11 – 3.02 (m, 1H), 2.21 – 2.11 (m, 1H), 2.10 – 1.98 (m, 1H), 1.97 – 1.83 (m, 1H), 1.81 – 1.65 (m, 2H), 1.64 – 1.54 (m, 1H), 1.52 – 1.44 (m, 2H), 1.32 – 1.27 (m, 6H), 1.26 (s, 9H), 0.93 – 0.81 (m, 6H).

$^{13}C\{^{1}H\}$ NMR (101 MHz, $CDCl_3$) δ_C ppm: 176.5, 172.4, 58.5, 48.6, 48.1, 46.8, 38.8, 31.8, 31.6, 31.1, 29.5, 28.5, 27.9, 27.5, 26.9, 26.8, 26.0, 22.8, 22.8, 14.2, 14.1.

HRMS: calc for $C_{24}H_{43}N_2O_2$ $[M+H]^+$: 367.3325, found: 367.3315

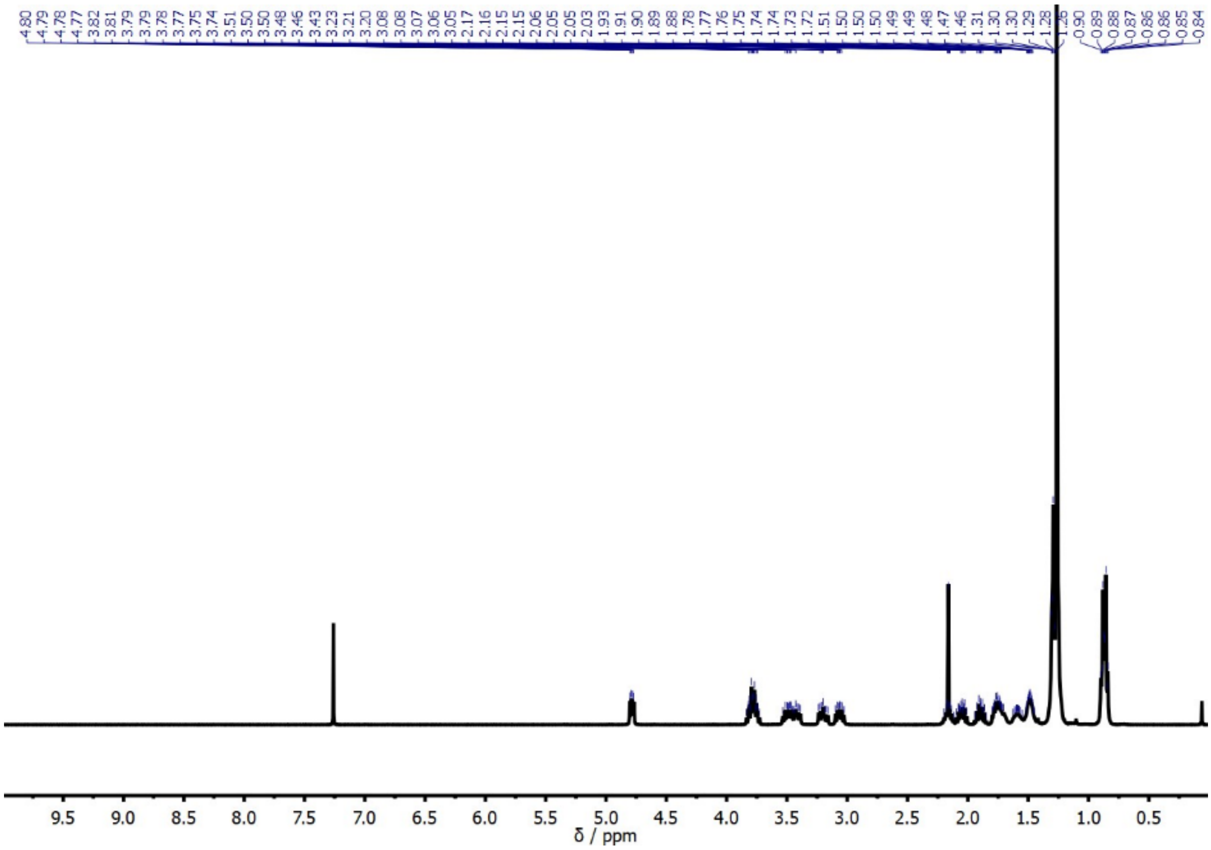

Figure S.7 – 400 MHz ^{1}H NMR spectrum of **2** in $CDCl_3$ (δ 0 – 10 ppm).

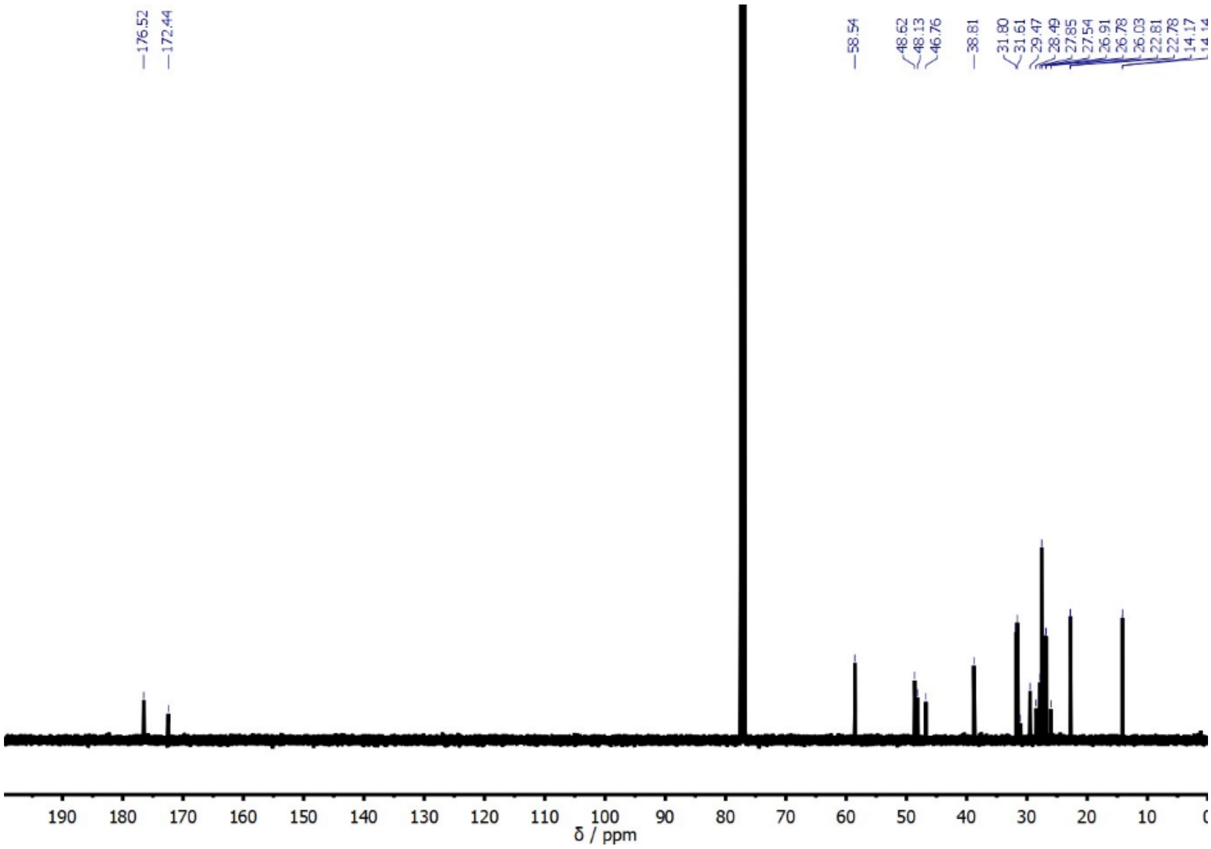

Figure S.8 – 101 MHz ^{13}C NMR spectrum of **2** in $CDCl_3$ (δ 0 – 200 ppm).

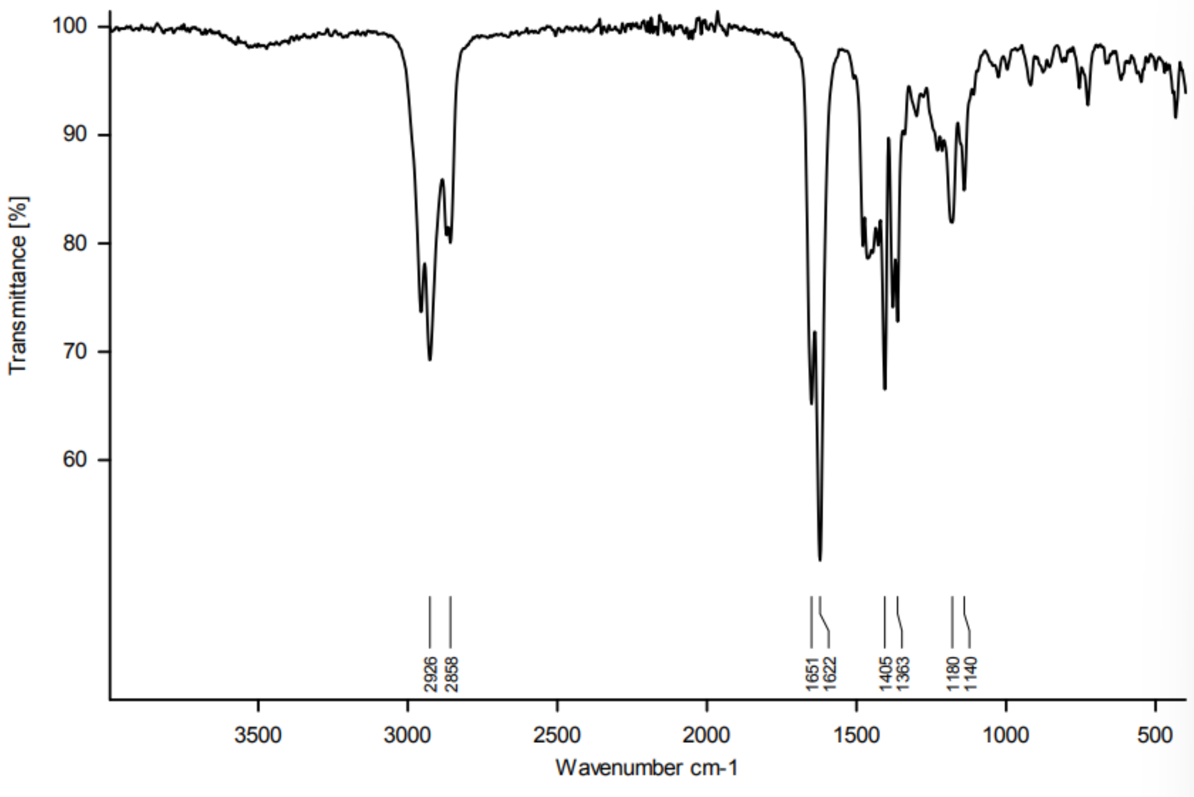

Figure S.9 – FT-IR spectrum of **2**

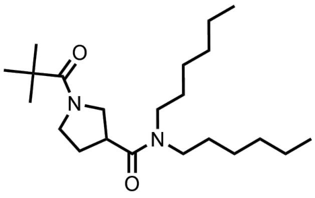

4

N,N-dihexyl-1-pivaloylpyrrolidine-3-carboxamide, **4**. *N*-boc-pyrrolidine-3-carboxylic acid (0.579 g, 2.69 mmol), EDC.HCl (0.676 g, 3.53 mmol) and DMAP (0.576 g, 5.13 mmol) were dissolved in dry DCM (15 mL) under N_2. Dihexylamine (0.55 mL, 2.95 mmol) was added and the reaction stirred for 4 days. More EDC.HCl (0.752 g, 3.92 mmol) and DMAP (0.632 g, 5.63 mmol) were added and the reaction stirred for 5 hours. The reaction mixture was diluted in Et_2O (50 mL), then washed with water (2 x 25 mL), saturated $NaHCO_3$ (25 mL) and brine (25 mL). The organic layer was dried with $MgSO_4$ and the solvent removed under reduced pressure. The residue was dissolved in dry DCM (5 mL) under N_2 and trifluoroacetic acid (5 mL) was added. The reaction was stirred for 45 minutes and the volatiles then removed under reduced pressure. The resulting oil was dissolved in THF (25 mL) and then evaporated to dryness, this was repeated 2 more times. The resulting residue was dissolved in dry DCM (10 mL) under N_2 and triethyl amine (10.0 mL, 71.5 mmol) was added. Then pivaloyl chloride (1.00 mL, 8.17 mmol) was added and the reaction stirred overnight. The reaction mixture was diluted with EtOAc (50 mL) and washed with 1 M HCl (25 mL), saturated $NaHCO_3$ solution (25 mL), water (25 mL) and brine (25 mL). The organic layer was dried with $MgSO_4$ and the solvent removed under reduced pressure. The crude mixture was purified by flash chromatography (petroleum ether/EtOAc on SiO_2 then DCM/MeOH on SiO_2) to yield the desired product as a clear oil (0.457 g, 1.25 mmol, 46%)

ν_{max} (film) cm^{-1} : 2955 (C-H), 2927 (C-H), 2858 (C-H), 1617 (C=O), 1477, 1461, 1407, 1378, 1362, 1189, 1142, 439

^1H NMR (400 MHz, $CDCl_3$) δ_H ppm: 3.84 (dd, J = 11.0, 7.9 Hz, 1H), 3.80 (s, 2H), 3.56 (s, 2H), 3.36 – 3.20 (m, 4H), 3.12 (s, 2H), 2.11 – 1.96 (m, 2H), 1.61 – 1.44 (m, 4H), 1.32 – 1.26 (m, 12H), 1.25 (s, 9H), 0.88 (dt, J = 9.2, 6.7 Hz, 6H).

$^{13}C\{^1H\}$ NMR (101 MHz, $CDCl_3$) δ_C ppm: 176.5, 172.5, , 48.0, 46.3, 39.1, 31.7, 31.7, 29.7, 27.8, 27.6, 26.8, 26.7, 22.7, 14.2, 14.1.

HRMS: calc for $C_{24}H_{42}N_2O_2Na$ $[M+Na]^+$: 389.3144, found: 289.3138.

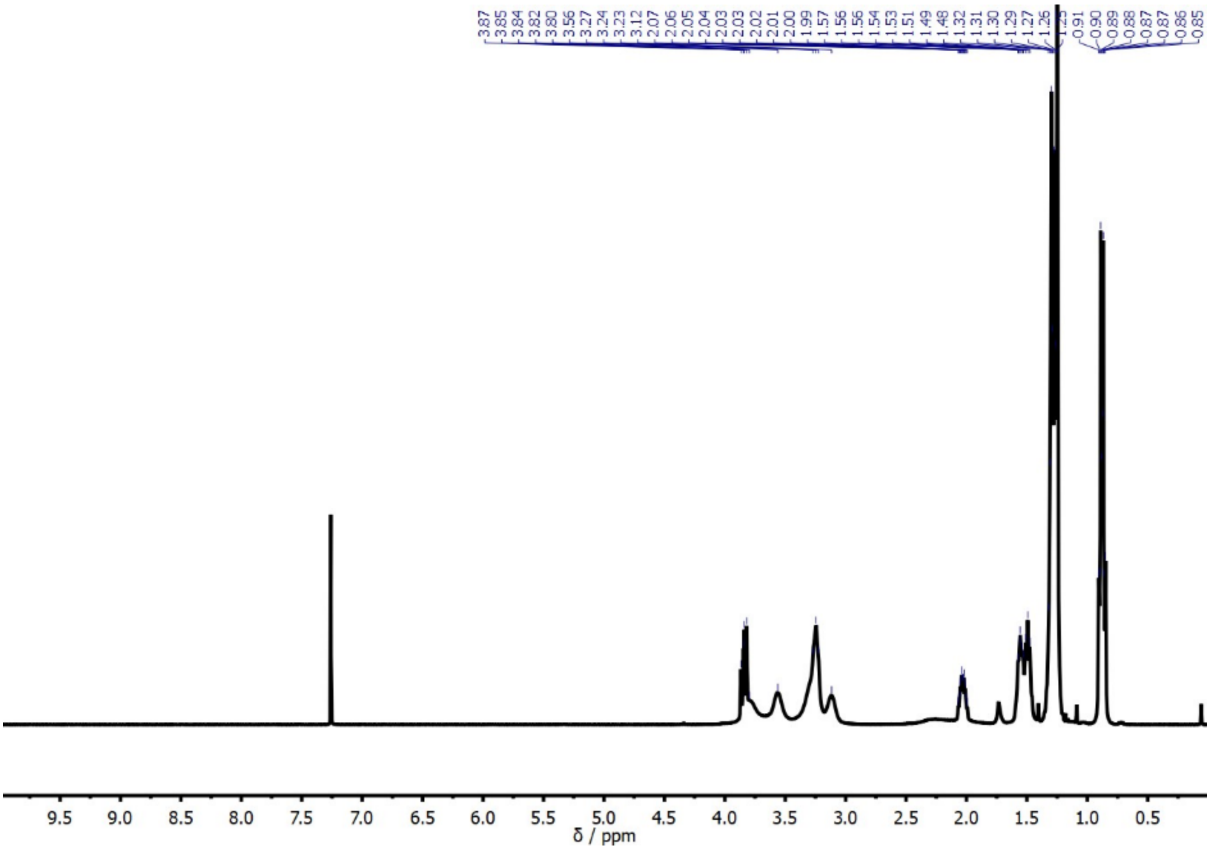

Figure S.10 – 400 MHz ^{1}H NMR spectrum of **4** in $CDCl_3$ (δ 0 – 10 ppm).

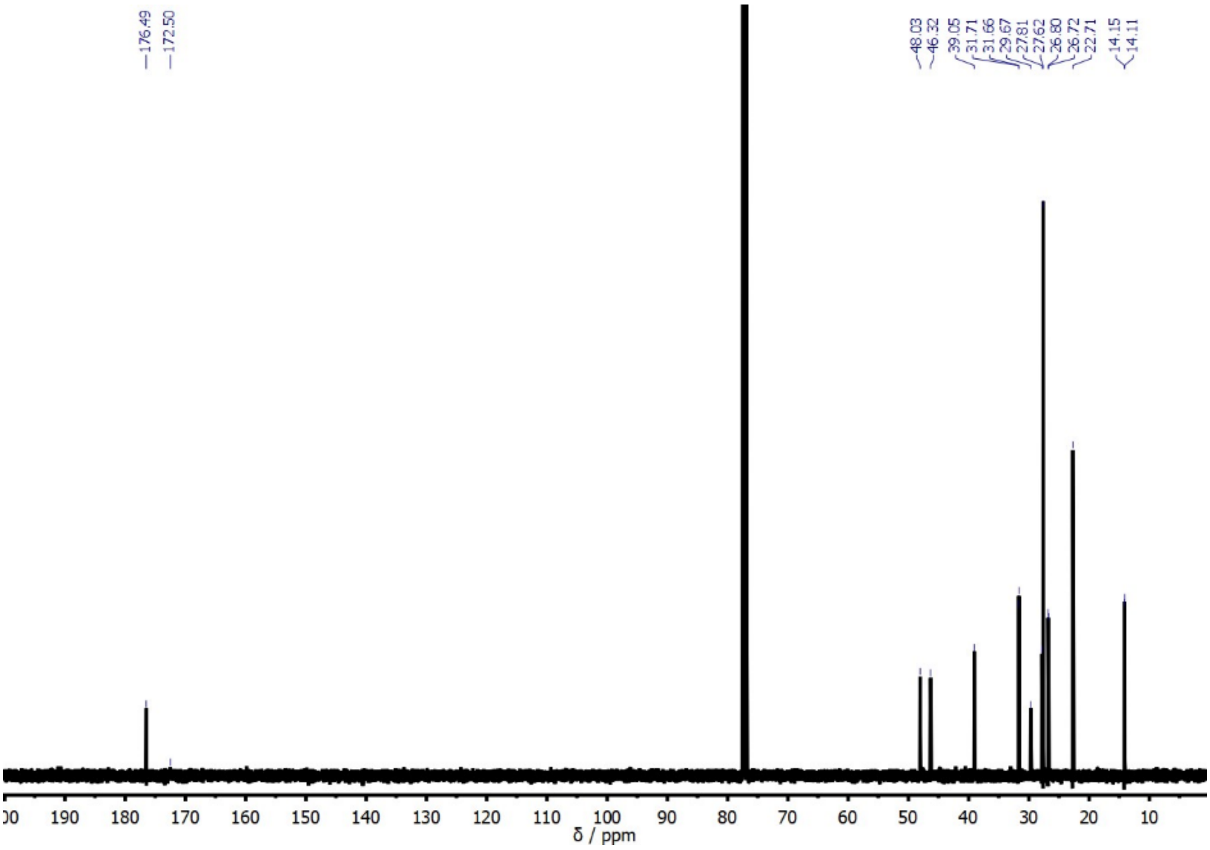

Figure S.11 – 101 MHz ^{13}C NMR spectrum of **4** in $CDCl_3$ (δ 0 – 200 ppm).

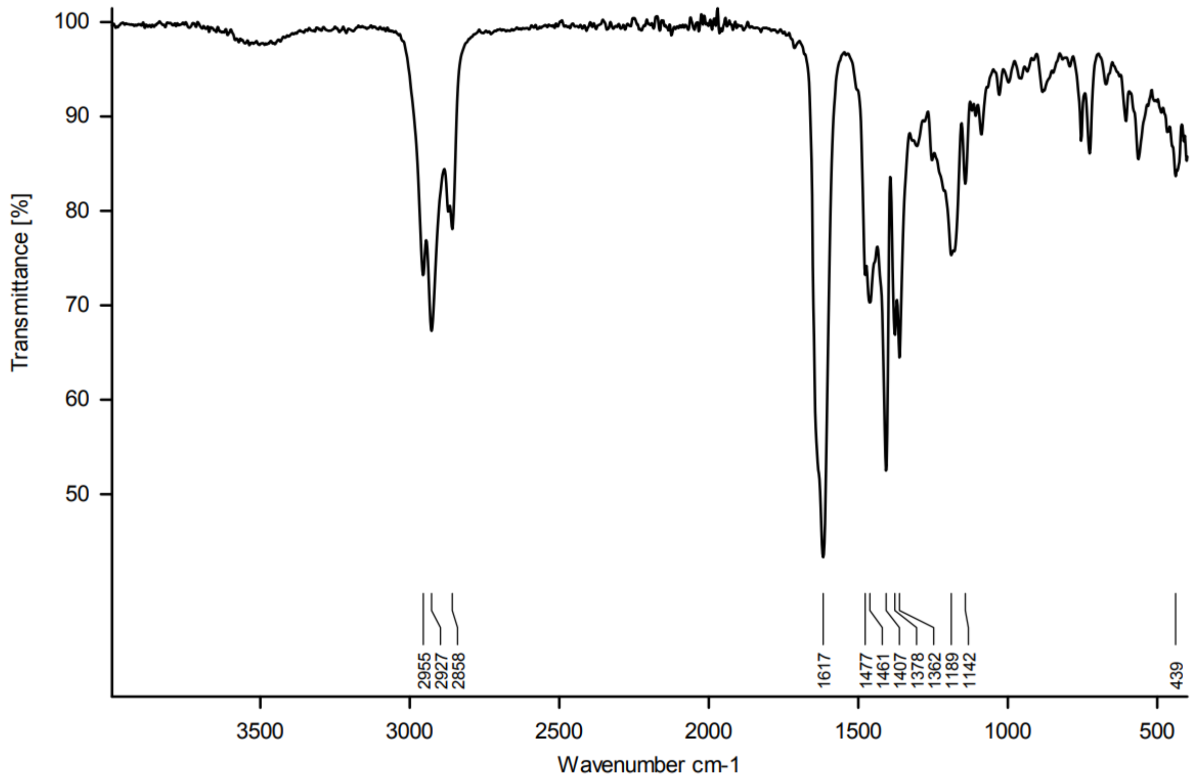

Figure S.12 – FT-IR spectrum of **4**

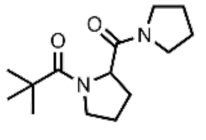

5

2,2-dimethyl-1-(2-(pyrrolidine-1-carbonyl)pyrrolidin-1-yl)propan-1-one, **5**. *N*-boc-L-proline (1.52 g, 7.07 mmol) and DIC (1.52 g, 12.0 mmol) were dissolved in dry DCM (6 mL) under N_2 at 0 °C and stirred for 30 minutes. Pyrrolidine (0.59 g, 7.1 mmol) dissolved in dry DCM (2.5 mL) was added, the reaction warmed to room temperature and the reaction stirred for 1 day. The solvent was removed in vacuo and the residue dissolved in EtOAc (25 mL) and filtered. The filtrate was then washed with 1 M HCl (10 mL), saturated $NaHCO_3$ (10 mL) and brine (10 mL). The organic layer was dried with $MgSO_4$ and the solvent removed under reduced pressure. The residue was dissolved in dry DCM (5 mL) under N_2 and trifluoroacetic acid (5 mL) was added. The reaction was stirred for 45 minutes and the volatiles then removed under reduced pressure. The resulting oil was dissolved in THF (25 mL) and then evaporated to dryness, this was repeated 2 more times. The resulting residue was dissolved in dry DCM (10 mL) under N_2 and triethyl amine (5 mL, 35.8 mmol) was added. Then pivaloyl chloride (1.00 mL, 8.17 mmol) was added and the reaction stirred overnight. The reaction mixture was diluted with EtOAc (50 mL) and washed with saturated $NaHCO_3$ solution (2 x 25 mL), 1 M HCl (25 mL), water (25 mL) and brine (25 mL). The organic layer was dried with $MgSO_4$ and the solvent removed under reduced pressure. The crude mixture was purified by recrystallisation from *n*-hexane (petroleum ether/EtOAc on SiO_2 then DCM/MeOH on SiO_2) to yield the desired product as a white crystalline solid (0.792 g, 3.14 mmol, 44%)

ν_{max} (film) cm^{-1} : 2968 (C-H), 2873 (C-H), 1650 (C=O), 1616 (C=O), 1478, 1434, 1407, 1380, 1363, 1339, 1191

^1H NMR (400 MHz, $CDCl_3$) δ_H ppm: 4.65 (dd, J = 8.5, 4.8 Hz, 1H), 3.85 – 3.70 (m, 3H), 3.55 (dt, J = 12.0, 6.9 Hz, 1H), 3.44 – 3.32 (m, 2H), 2.18 (dq, J = 13.0, 6.6 Hz, 1H), 2.10 – 1.73 (m, 7H), 1.26 (s, 9H).

$^{13}C\{^1H\}$ NMR (101 MHz, $CDCl_3$) δ_C ppm: 176.7, 171.3, 60.0, 48.6, 46.3, 45.9, 38.8, 27.5, 27.5, 26.3, 26.1, 24.3.

HRMS: calc for $C_{14}H_{25}N_2O_2$ $[M+H]^+$: 253.1916, found: 253.1931.

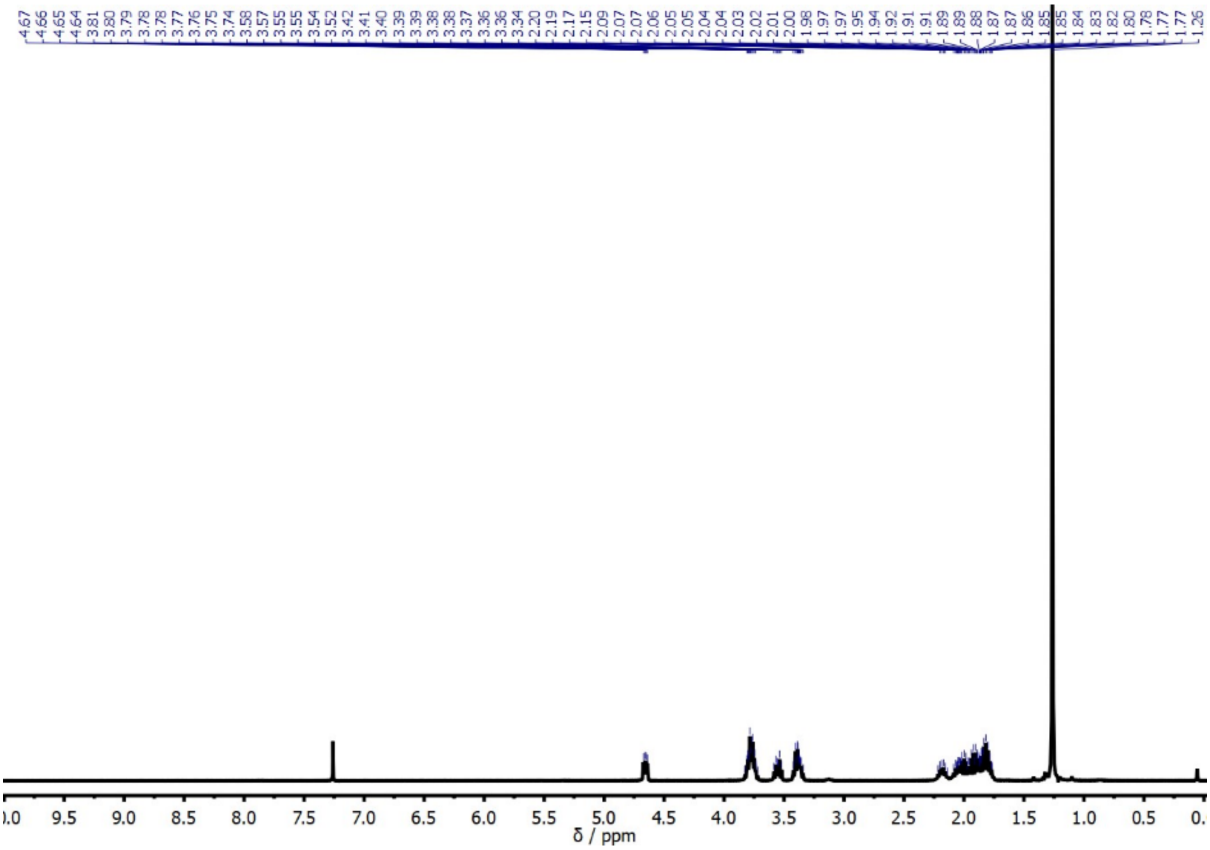

Figure S.13 – 400 MHz ^{1}H NMR spectrum of **5** in $CDCl_3$ (δ 0 – 10 ppm).

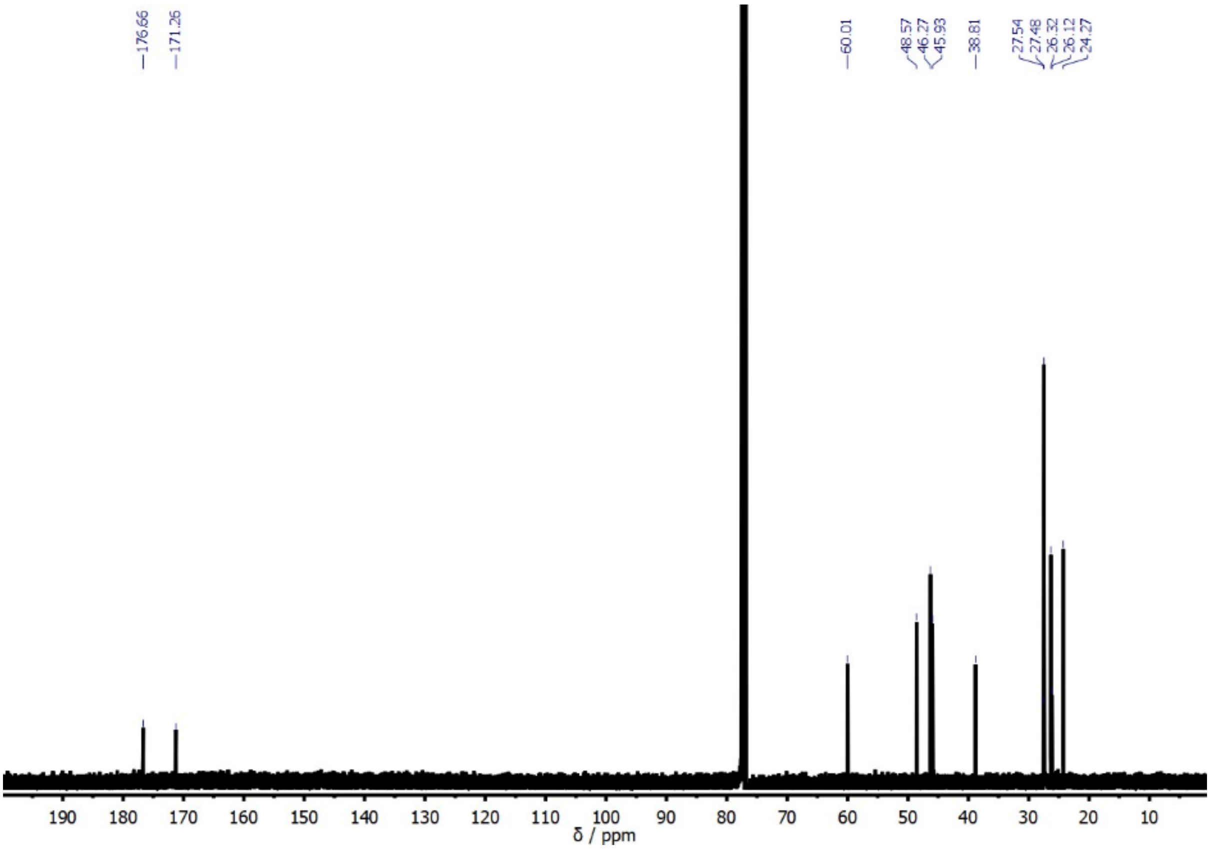

Figure S.14 – 101 MHz ^{13}C NMR spectrum of **5** in $CDCl_3$ (δ 0 – 200 ppm).

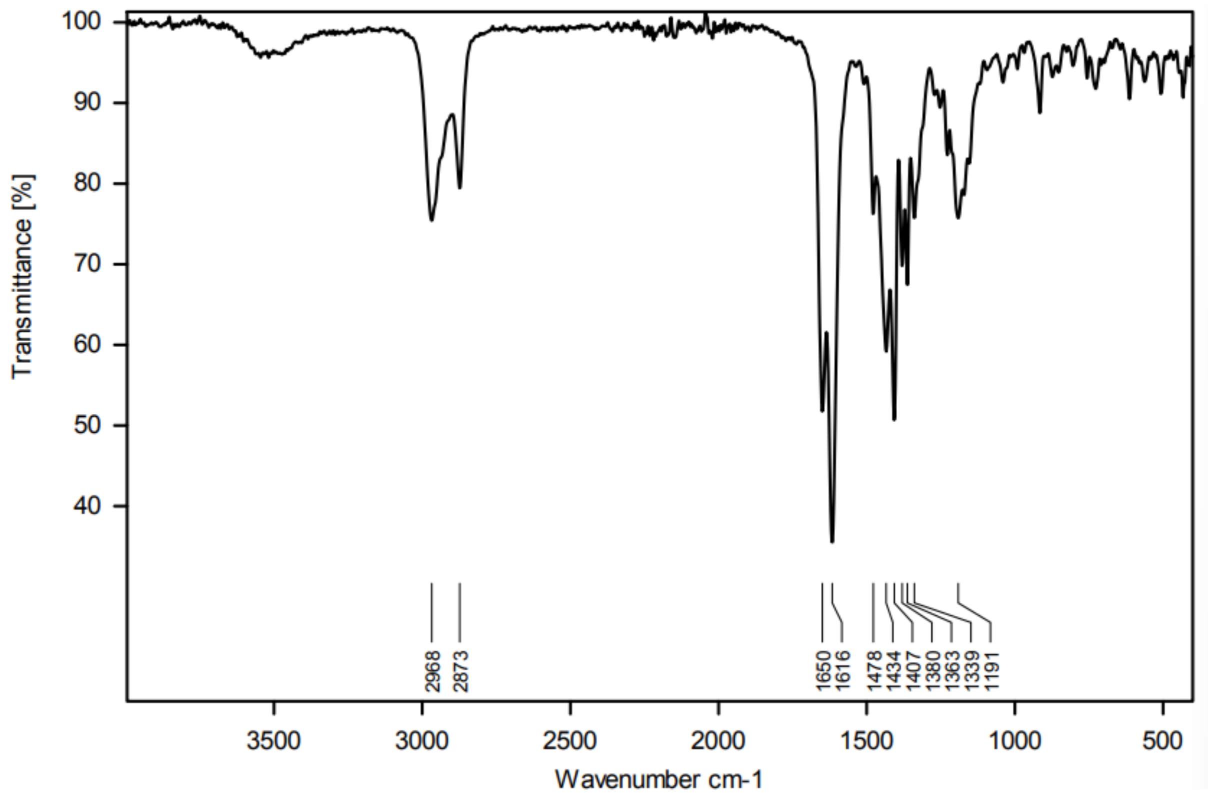

Figure S.15 – FT-IR spectrum of **5**

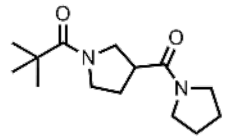

6

2,2-dimethyl-1-(3-(pyrrolidine-1-carbonyl)pyrrolidin-1-yl)propan-1-one, **6**. *N*-boc-pyrrolidine-3-carboxylic acid (0.494 g, 2.30 mmol), EDC.HCl (0.598 g, 3.19 mmol) and DMAP (0.453 g, 4.03 mmol) were dissolved in dry DCM (10 mL) under N_2. Pyrrolidine (0.20 mL, 2.4 mmol) was added and the reaction stirred overnight. The reaction mixture was diluted in Et_2O (50 mL), then washed with water (2 x 25 mL), saturated $NaHCO_3$ (25 mL) and brine (25 mL). The organic layer was dried with $MgSO_4$ and the solvent removed under reduced pressure. The residue was dissolved in dry DCM (5 mL) under N_2 and trifluoroacetic acid (5 mL) was added. The reaction was stirred for 60 minutes and the volatiles then removed under reduced pressure. The resulting oil was dissolved in THF (25 mL) and then evaporated to dryness, this was repeated 2 more times. The resulting residue was dissolved in dry DCM (10 mL) under N_2 and triethyl amine (5 mL, 35.8 mmol) was added. Then pivaloyl chloride (1.00 mL, 8.17 mmol) was added and the reaction stirred for 3 days. The reaction mixture was diluted with EtOAc (50 mL) and washed with saturated $NaHCO_3$ solution (25 mL), 1 M HCl (25 mL), water (25 mL) and brine (25 mL). The organic layer was dried with $MgSO_4$ and the solvent removed under reduced pressure. The crude mixture was purified by flash chromatography (petroleum ether/EtOAc on SiO_2 then DCM/MeOH on SiO_2) to yield the desired product as a clear oil (0.162 g, 0.642 mmol, 20%)

ν_{max} (film) cm^{-1}: 2968 (C-H), 2875 (C-H), 1689 (C=O), 1615 (C=O), 1442, 1409, 1382, 1364, 1228, 1197, 1169, 1145, 922, 729

^1H NMR (400 MHz, $CDCl_3$) δ_H ppm: 3.93 – 3.69 (m, 2H), 3.61 – 3.52 (m, 1H), 3.51-3.41 (m, 4H), 3.05 (s, 1H), 2.06 (h, J = 6.3 Hz, 2H), 1.97 (p, J = 6.8 Hz, 2H), 1.86 (p, J = 6.9 Hz, 2H), 1.24 (s, 9H).

$^{13}C\{^1H\}$ NMR (101 MHz, $CDCl_3$) δ_C ppm: 176.6, 170.9, 50.7, 47.6, 46.7, 46.1, 39.0, 27.6, 26.3, 24.4

HRMS: calc for $C_{14}H_{25}N_2O_2$ $[M+H]^+$: 253.1916, found: 253.1924.

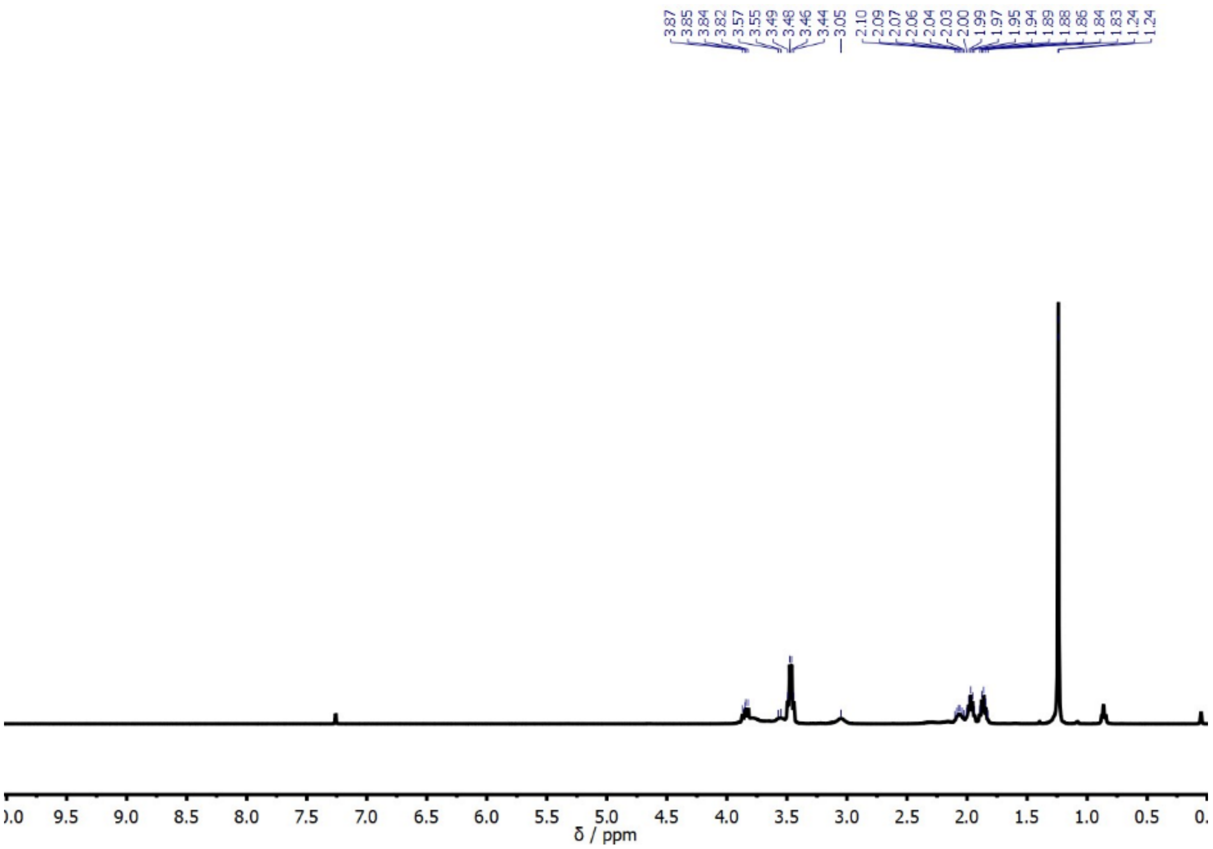

Figure S.16 – ^{1}H NMR spectrum of **6** in $CDCl_3$ (δ 0 – 10 ppm).

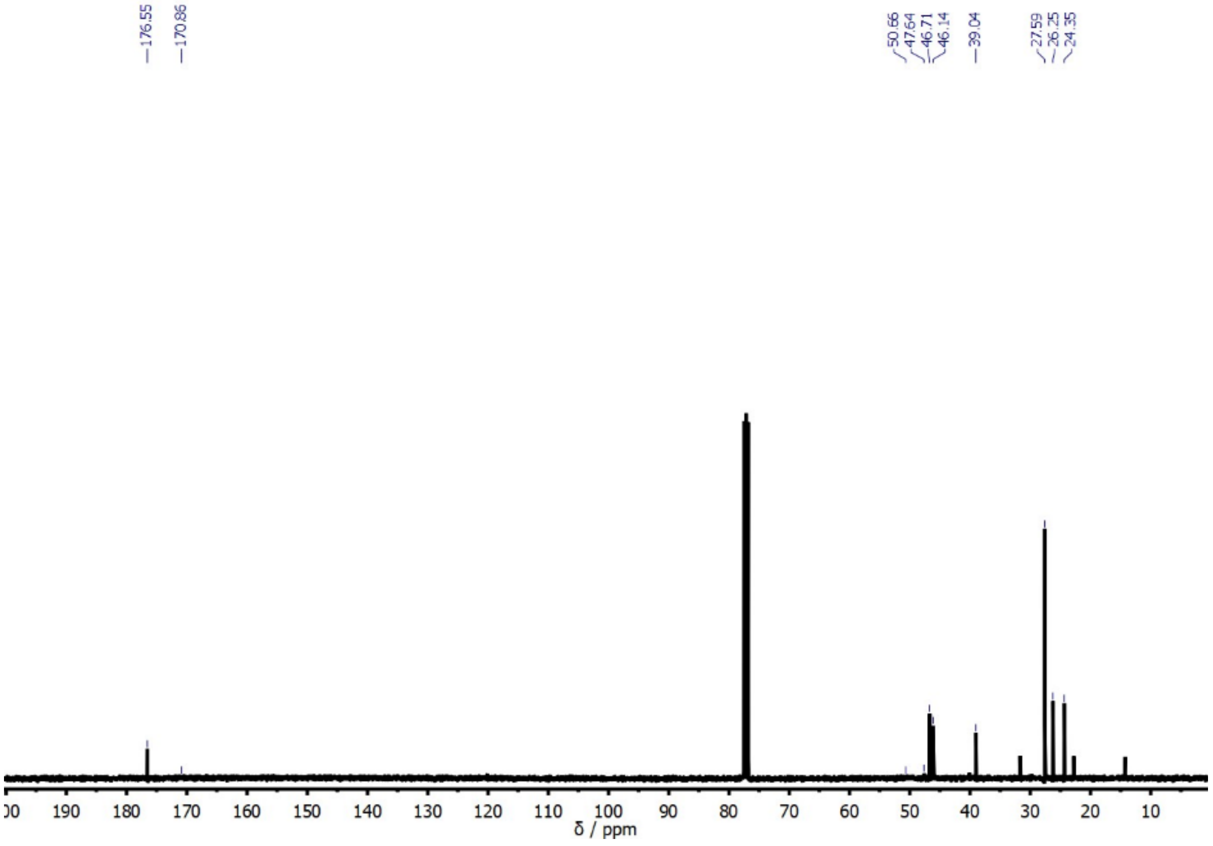

Figure S.17 – ^{13}C NMR spectrum of **6** in $CDCl_3$ (δ 0 – 200 ppm).

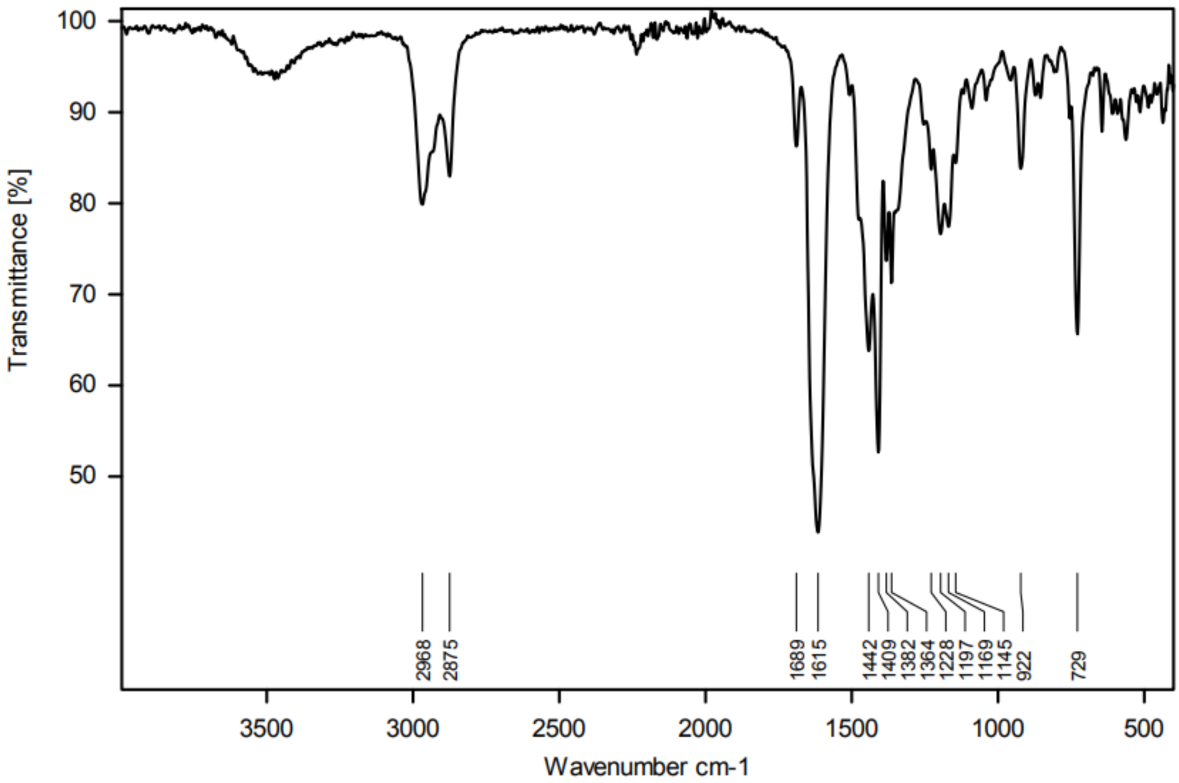

Figure S.18 – FT-IR spectrum of **6**

UV-vis Absorption and NMR Titrations

UV-vis titrations were carried out on an Agilent Cary 60 UV-Vis spectrophotometer, using standard titration protocols. A 5 mL sample of the host (2-methyl-4-nitroaniline) was prepared at a known concentration (0.015 mM) in *n*-octane. The UV-vis spectrum of the free host (2 mL) was recorded with 2 mL in the cuvette. The guest was dissolved (**1**-**4**) in 2 mL of the host solution at a known concentration. Aliquots of the guest solution were successively added to the cuvette, and the UV-vis absorption spectrum was recorded after each addition. The UV-vis absorption spectra were analysed using Musketeer to fit the changes in the absorption at fixed wavelengths to either a 1:1 binding isotherm by optimizing the association constant and absorption of the free and bound host and accounting for guest adsorption.[2]

^{1}H NMR titrations were carried out on a Bruker 500 MHz spectrometer, using standard titration protocols. A 2 mL sample of the host (**1**-**4**) was prepared at a concentration of 9-90 mM in cyclohexane-d_{12}. The ^{1}H NMR spectrum of the host solution (0.6 mL) was recorded. The guest (PFTB) was dissolved in 1 mL of the host solution at a known concentration. Aliquots of the guest solution were successively added to the NMR sample tube containing the host solution, and the ^{1}H NMR spectrum was recorded after each addition.

For the ^{1}H dilution experiments, 0.6 mL of the host in *n*-octane at a known concentration was placed in an NMR tube and aliquots of a solution pure *n*-octane was added with the spectrum being recorded after every addition in a Bruker 500 MHz spectrometer.

^{13}C NMR titrations were carried out on a Bruker 700 MHz spectrometer fitted with a cryoprobe, using standard titration protocols. A 2 mL sample of the host (**1**-**4**) was prepared at a concentration of 50-90 mM in *n*-octane. The ^{13}C NMR spectrum of the host solution (0.6 mL) was recorded. The guest (PFTB) was dissolved in 1 mL of the host solution at a known concentration. Aliquots of the guest solution were successively added to the NMR sample tube containing the host solution, and the ^{13}C NMR spectrum was recorded after each addition.

The NMR spectra were analysed using Musketeer to fit the changes in the chemical shifts for different protons to either a 1:2 or a 1:3 binding isotherm by optimizing the association constant and shifts of the free and bound host.[2]

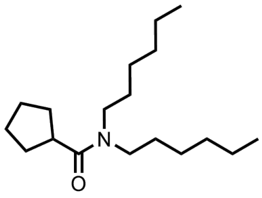

1

Figure S.19 – Structure of the compound **1**

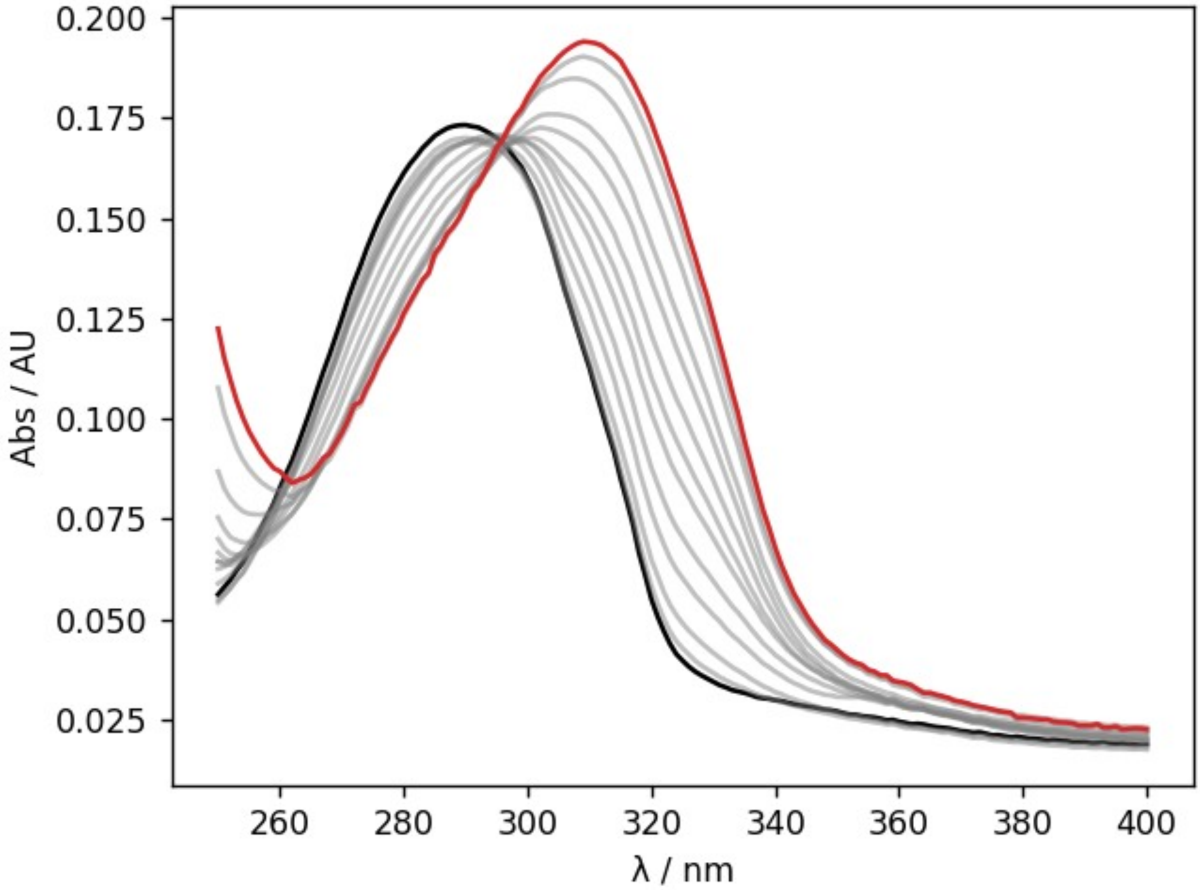

Figure S.20 - UV/Vis absorption spectra for the titration of **1** into 2-methyl-4-nitrophenol (0.015 mM in *n*-octane, at 298K). The UV/Vis spectrum of 2-methyl-4-nitrophenol and the final point of the titration are reported in black and in red, respectively.

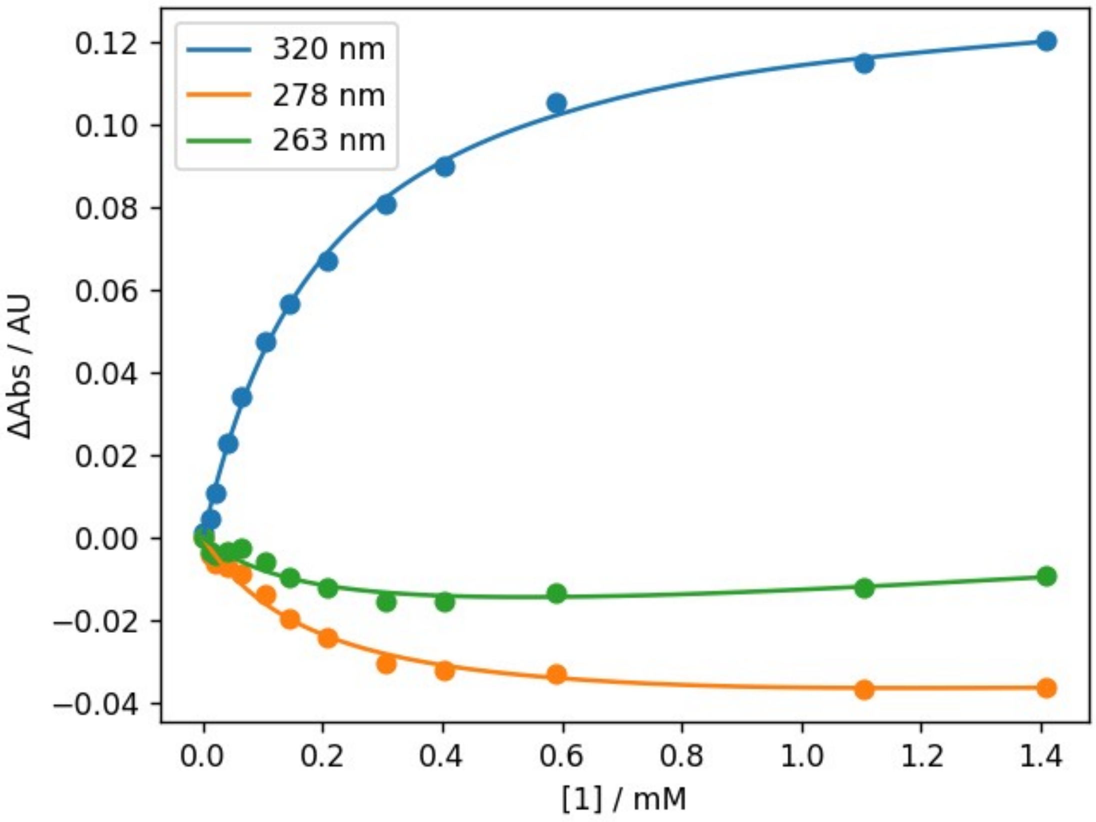

Figure S.21 - The fit of the absorbance at selected wavelengths to a 1:1 binding isotherm accounting for guest absorption for the titration of **1** into 2-methyl-4-nitrophenol (0.015 mM in *n*-octane, at 298 K).

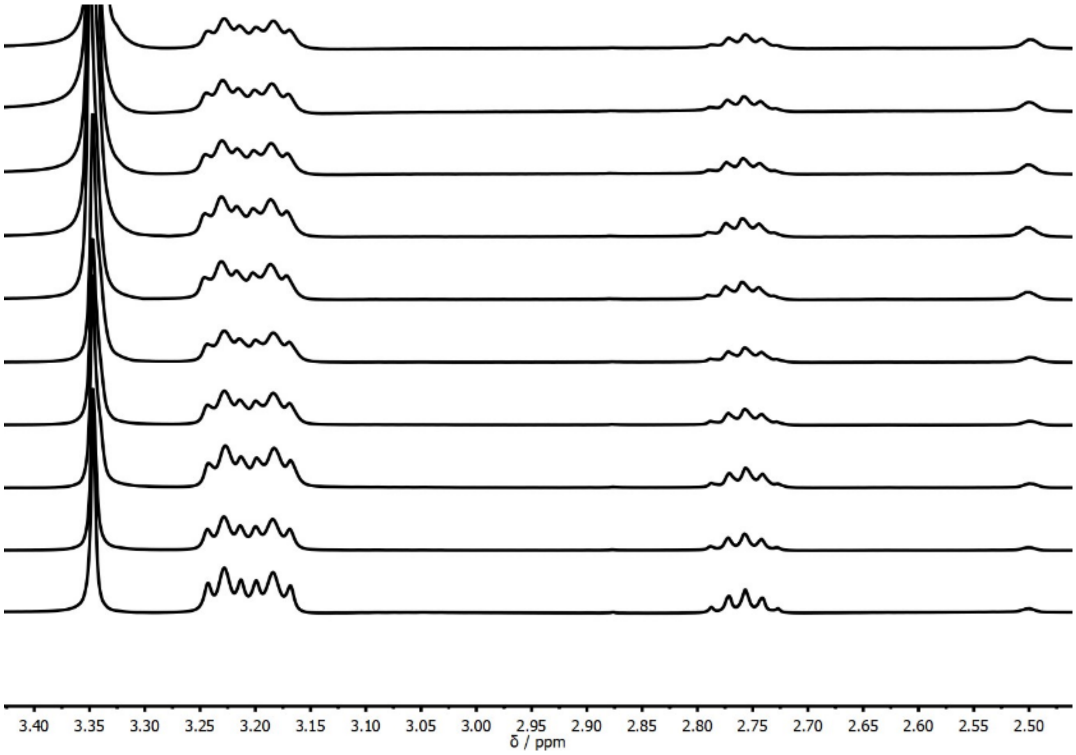

Figure S.22 – Stack for the 500 MHz ^{1}H NMR dilution of **1** in *n*-octane with concentration ranging from 20 mM (bottom) to 2.7 mM (top)

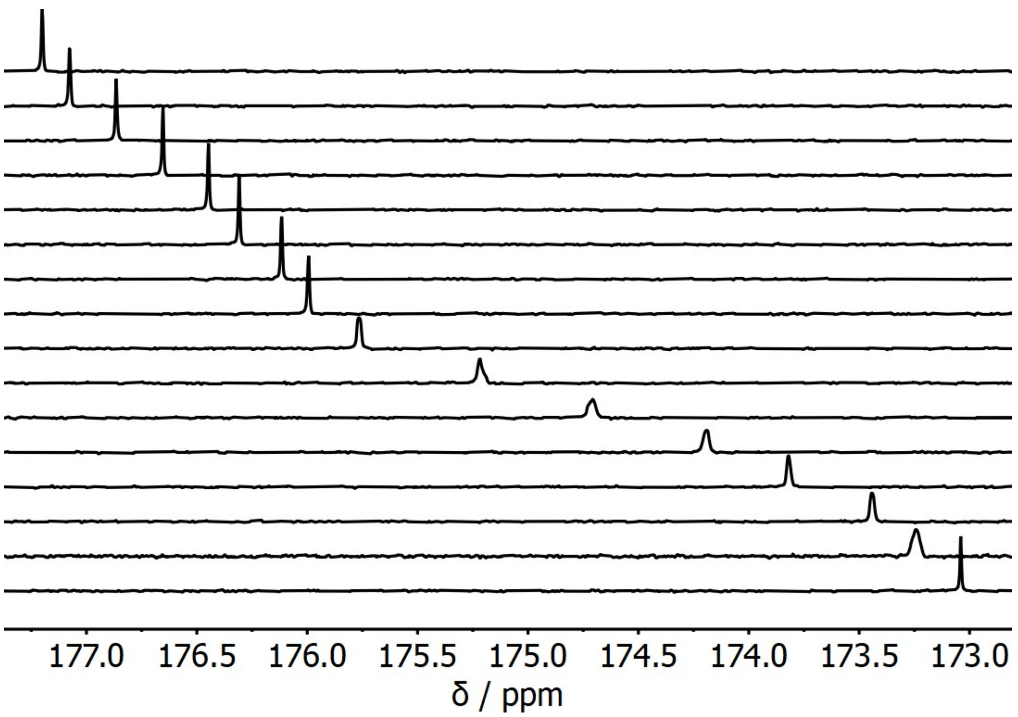

Figure S.23 – Stack for the 176 MHz ^{13}C NMR titration of PFTB into **1** (69 mM) in *n*-octane at 298 K

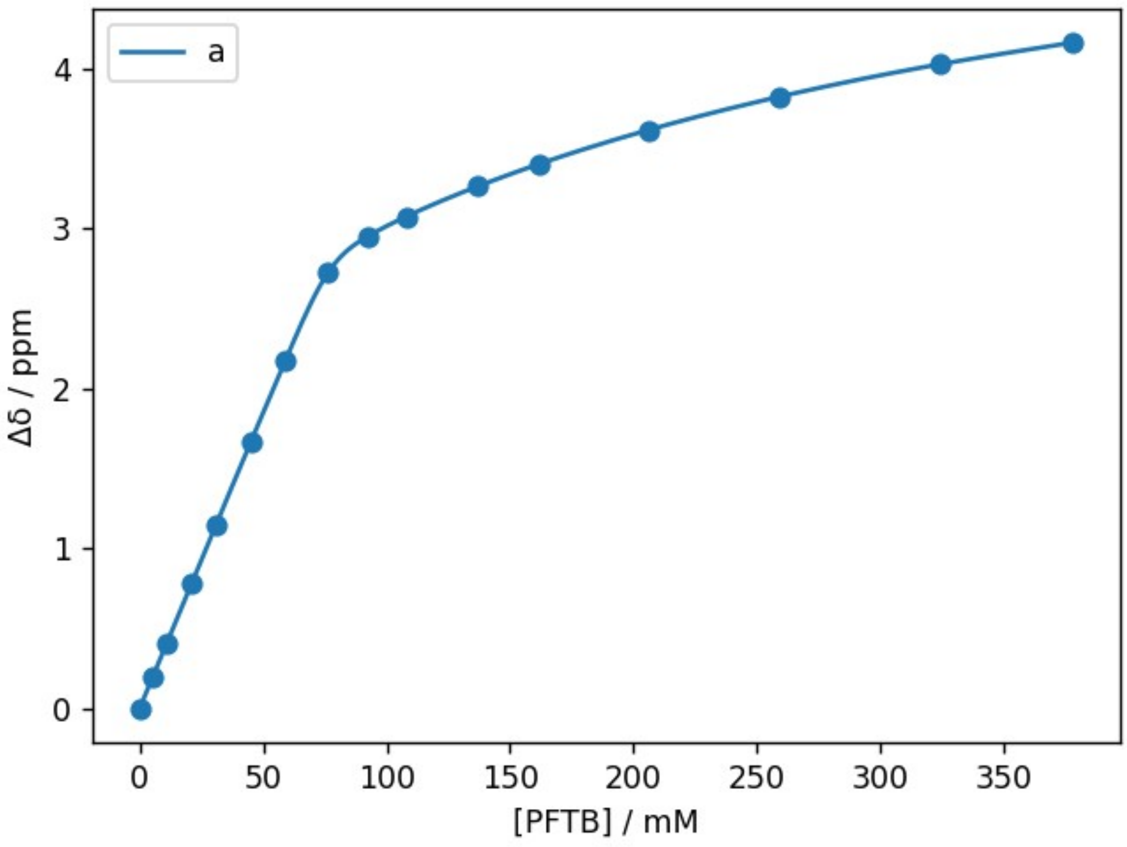

Figure S.24 – Fit of the chemical shifts for the ^{13}C NMR titration of PFTB into **1** (69 mM) in *n*-octane at 298K to a 1:2 binding isotherm.

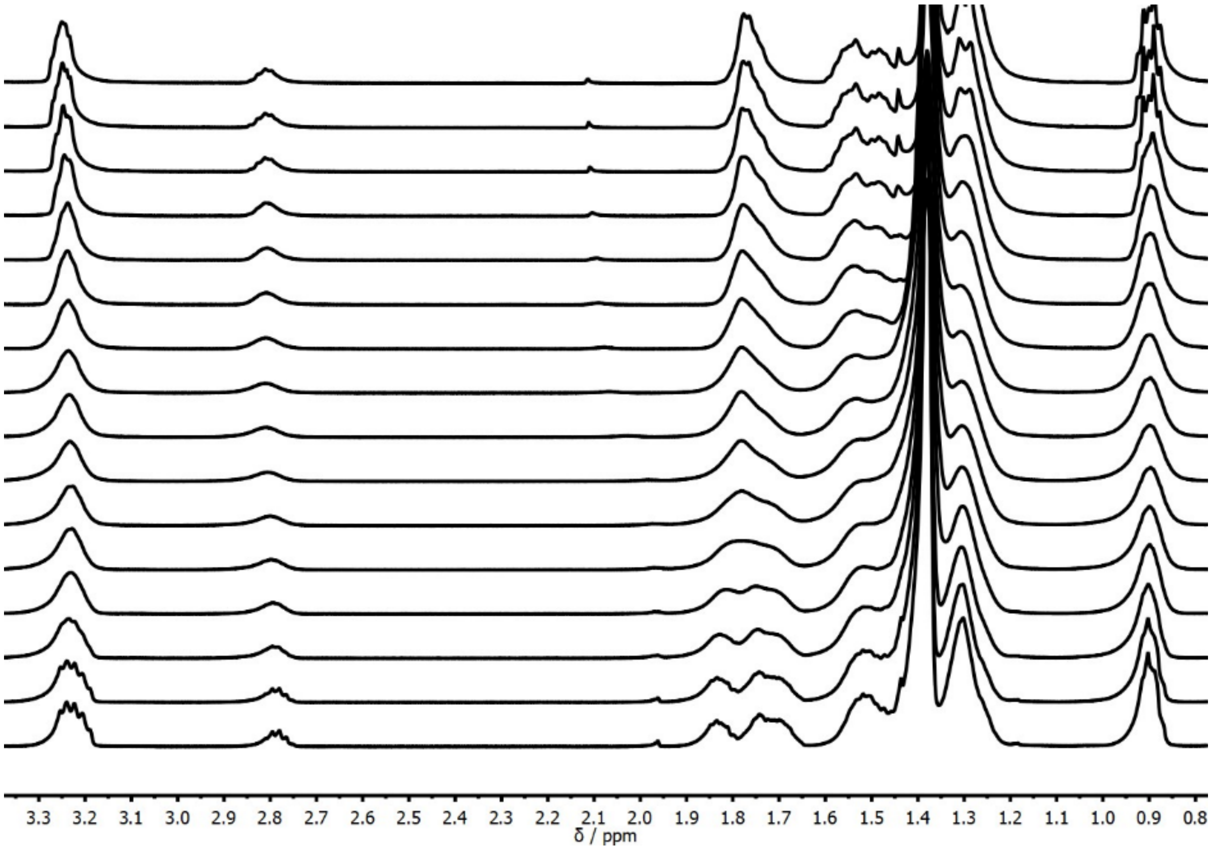

Figure S.25 - 500 MHz ^{1}H NMR spectra for titration of PFTB oxide into **1** (12 mM in cyclohexane-d_{12}, at 298 K)

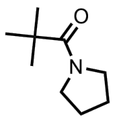

2

Figure S.26 – Structure of the compound **2**

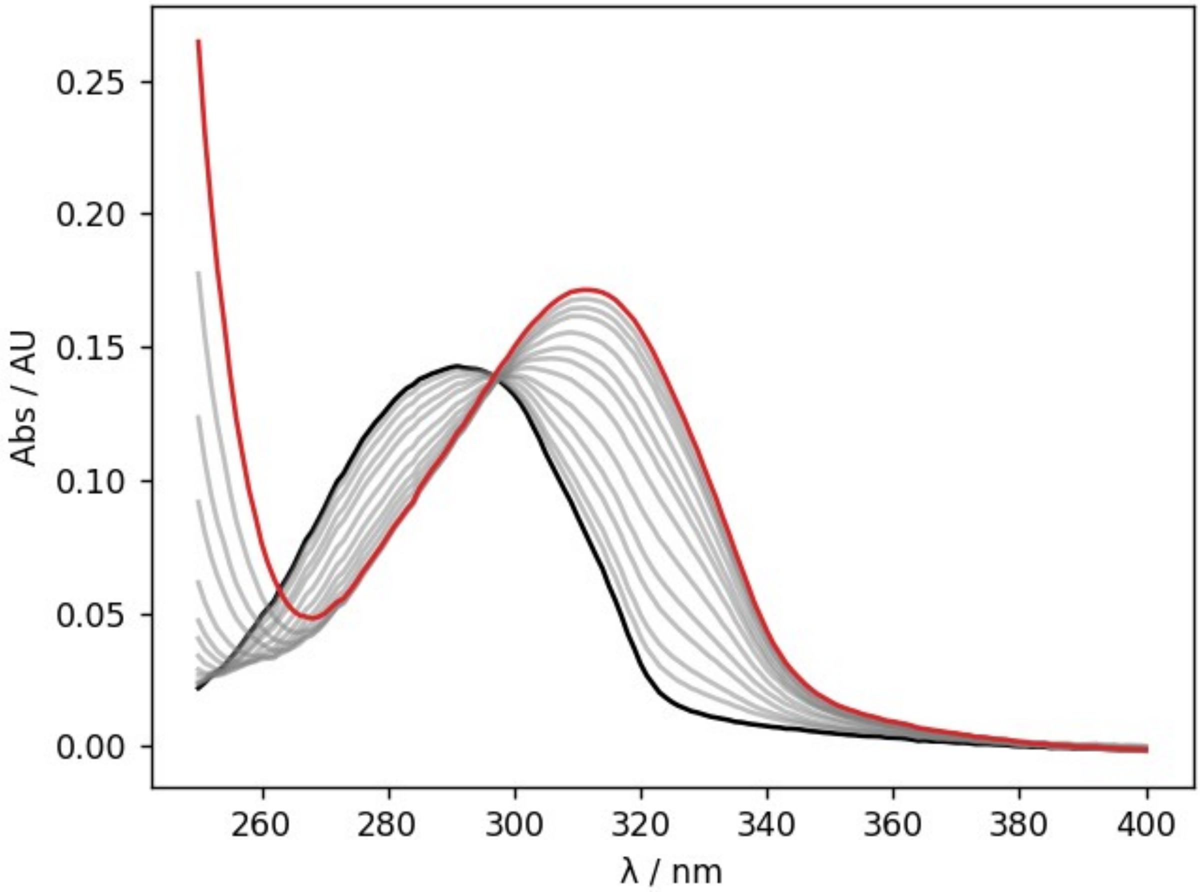

Figure S.27 - UV/Vis absorption spectra for the titration of **2** into 2-methyl-4-nitrophenol (0.015 mM in *n*-octane, at 298K). The UV/Vis spectrum of 2-methyl-4-nitrophenol and the final point of the titration are reported in black and in red, respectively.

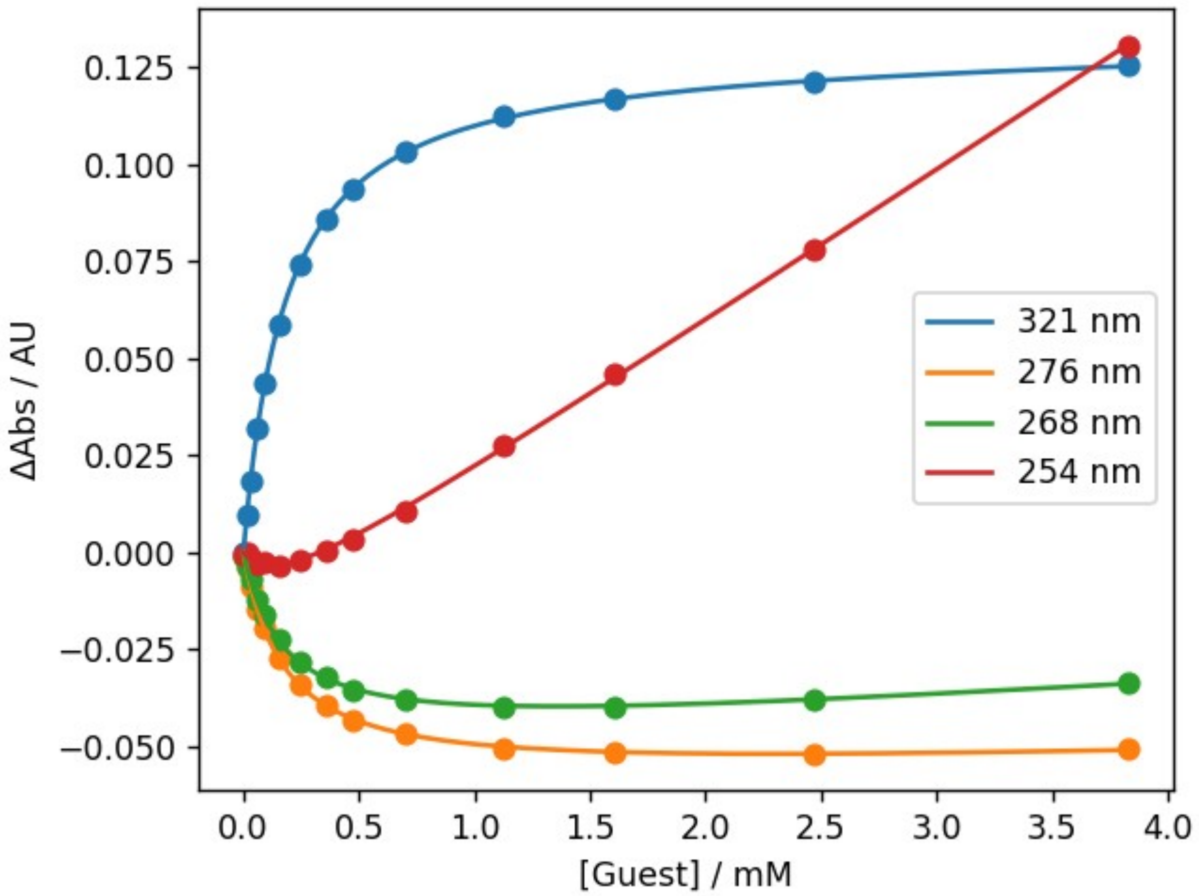

Figure S.28 - The fit of the absorbance at selected wavelengths to a 1:1 binding isotherm accounting for guest absorption for the titration of **2** into 2-methyl-4-nitrophenol (0.015 mM in *n*-octane, at 298 K).

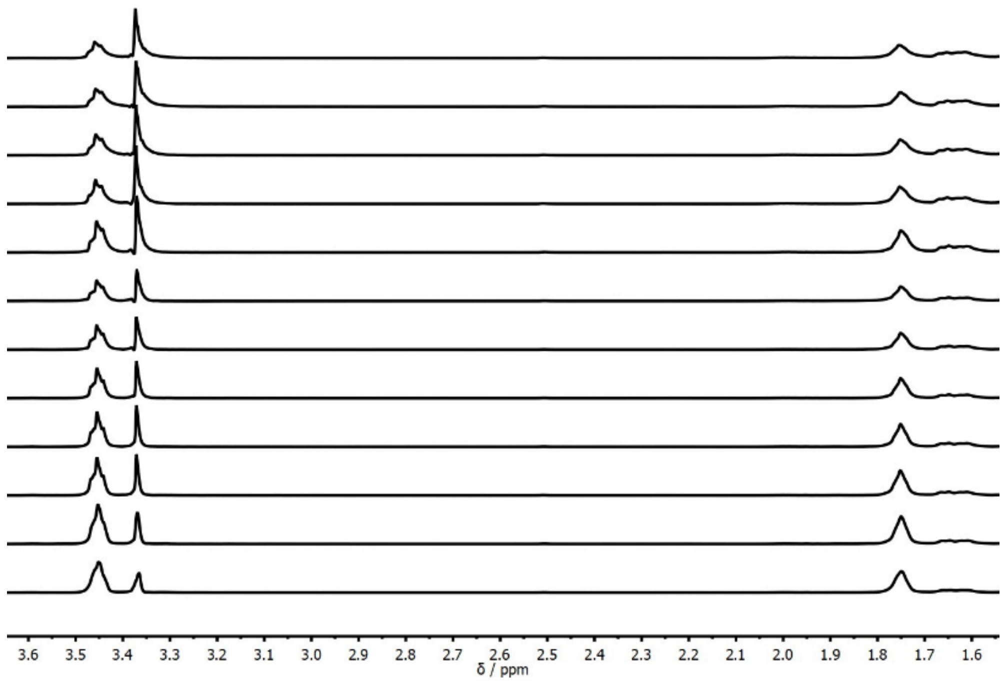

Figure S.29 – Stack plot for the 500 MHz ^{1}H NMR dilution of **2** in *n*-octane with concentration ranging from 62 mM (bottom) to 8.1 mM (top)

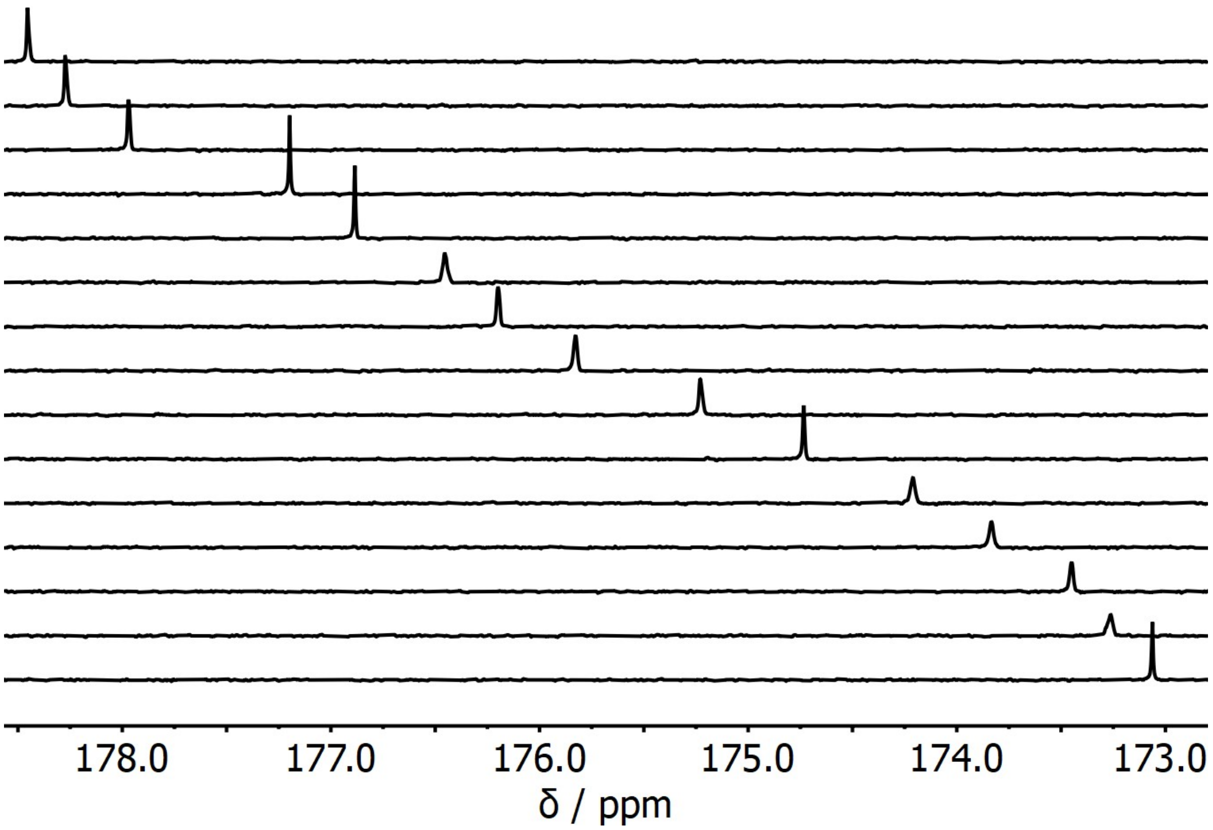

Figure S.30 – Stack for the 176 MHz ^{13}C NMR titration of PFTB into **2** (92 mM) in *n*-octane at 298 K

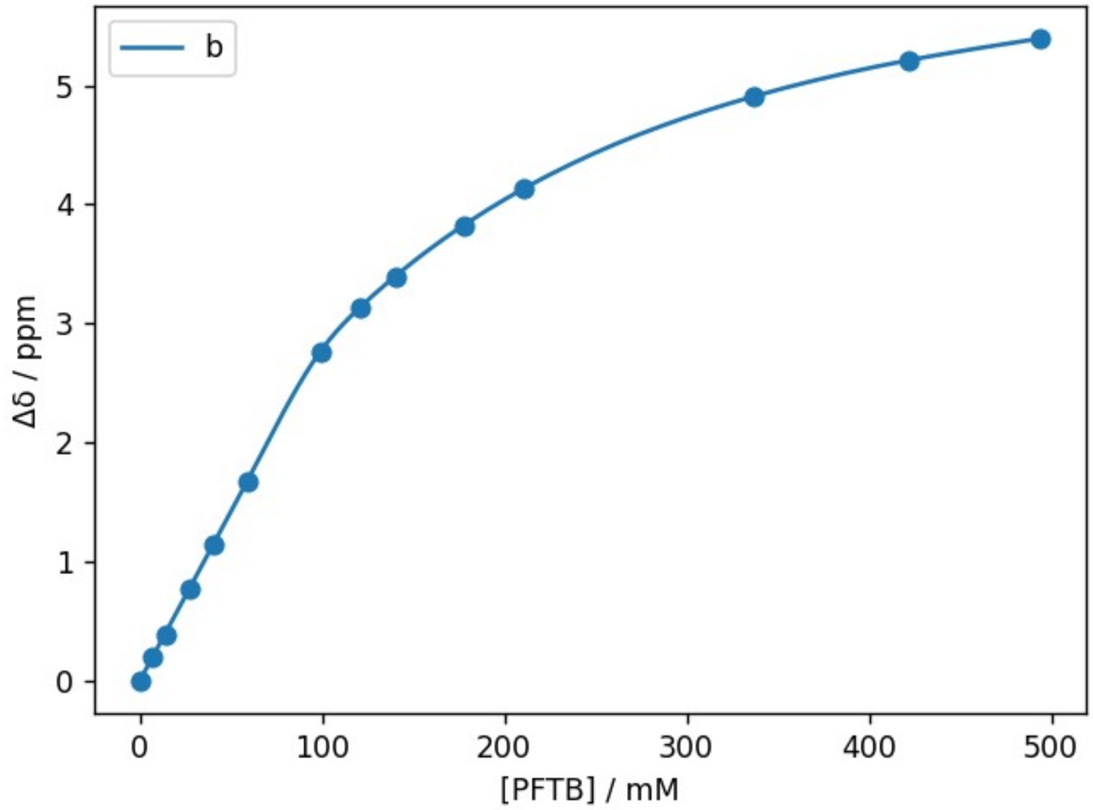

Figure S.31 – Fit of the chemical shifts for the ^{13}C NMR titration of PFTB into **2** (92 mM) in *n*-octane at 298K to a 1:2 binding isotherm.

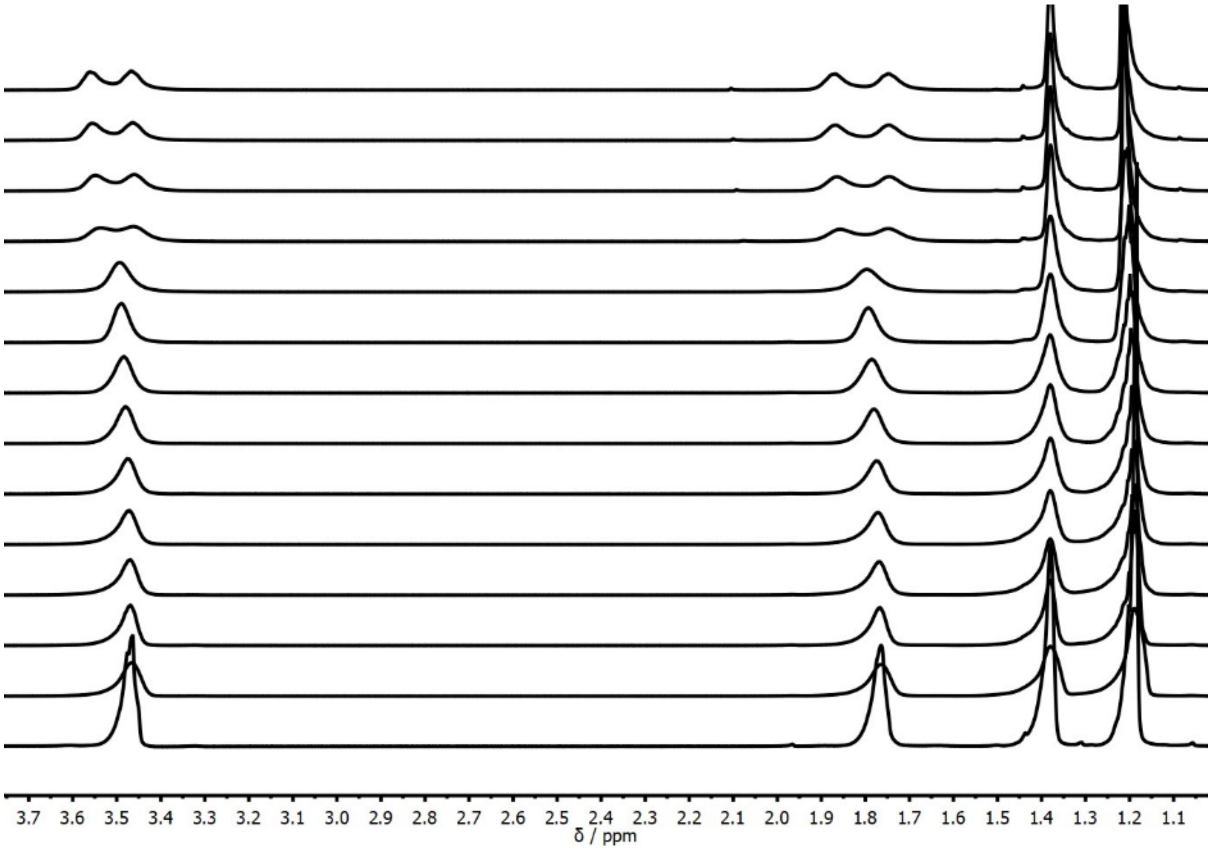

Figure S.32 - 500 MHz ^{1}H NMR spectra for titration of PFTB oxide into **2** (60 mM in cyclohexane-d_{12}, at 298 K)

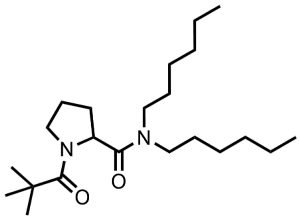

3

Figure S.33 – Structure of the compound **3**

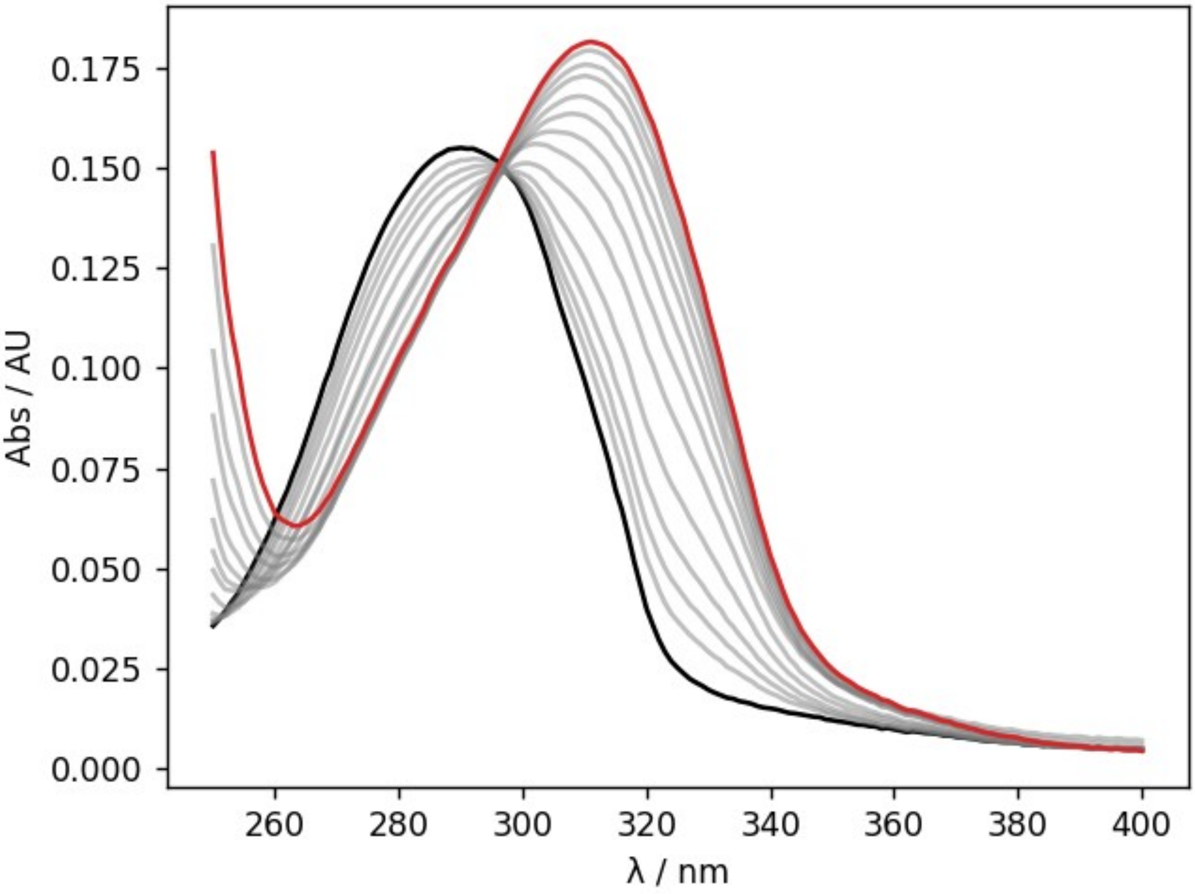

Figure S.34 - UV/Vis absorption spectra for the titration of **3** into 2-methyl-4-nitrophenol (0.015 mM in *n*-octane, at 298K). The UV/Vis spectrum of 2-methyl-4-nitrophenol and the final point of the titration are reported in black and in red, respectively.

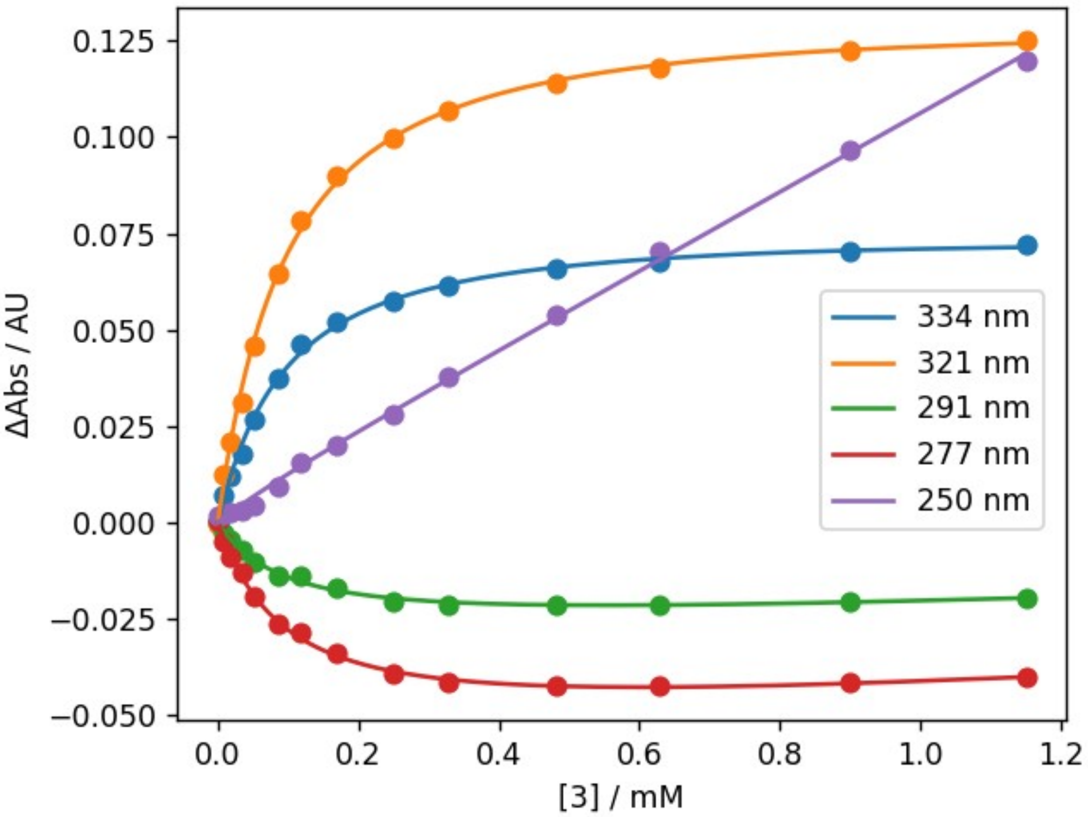

Figure S.35 - The fit of the absorbance at selected wavelengths to a 1:1 binding isotherm accounting for guest absorption for the titration of **3** into 2-methyl-4-nitrophenol (0.015 mM in *n*-octane, at 298 K).

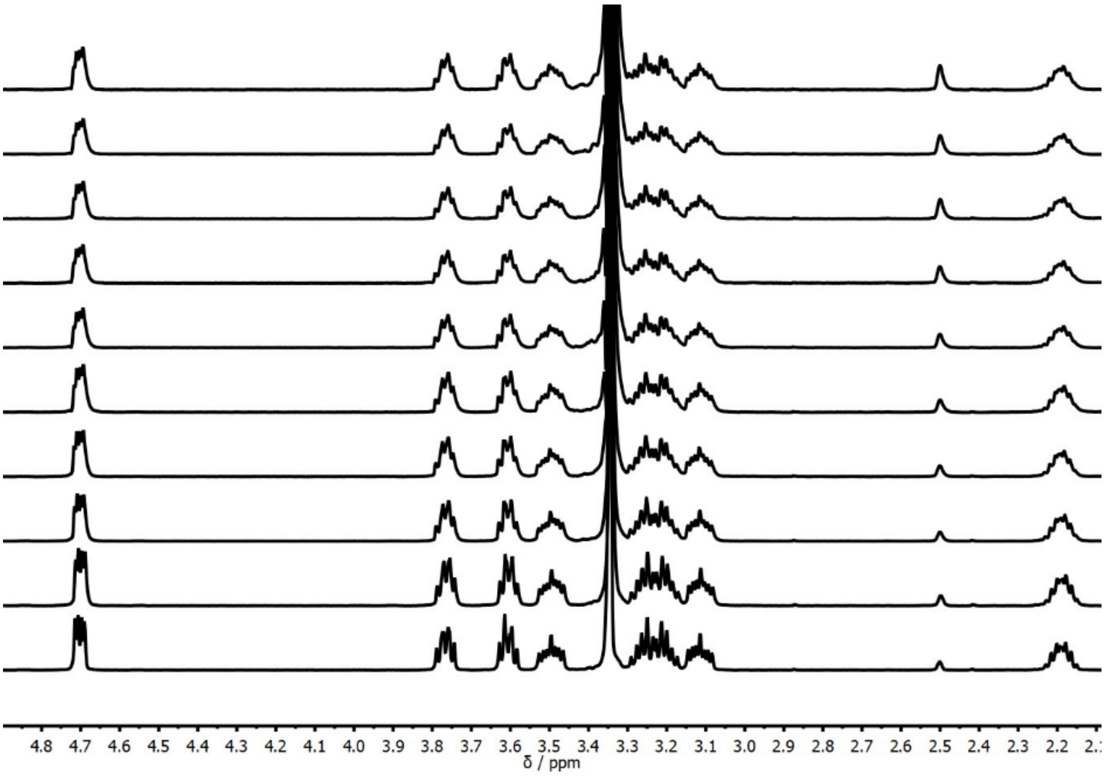

Figure S.36– Stack plot for the 500 MHz ^{1}H NMR dilution of **3** in *n*-octane with concentration ranging from 11.5 mM (bottom) to 1.5 mM (top)

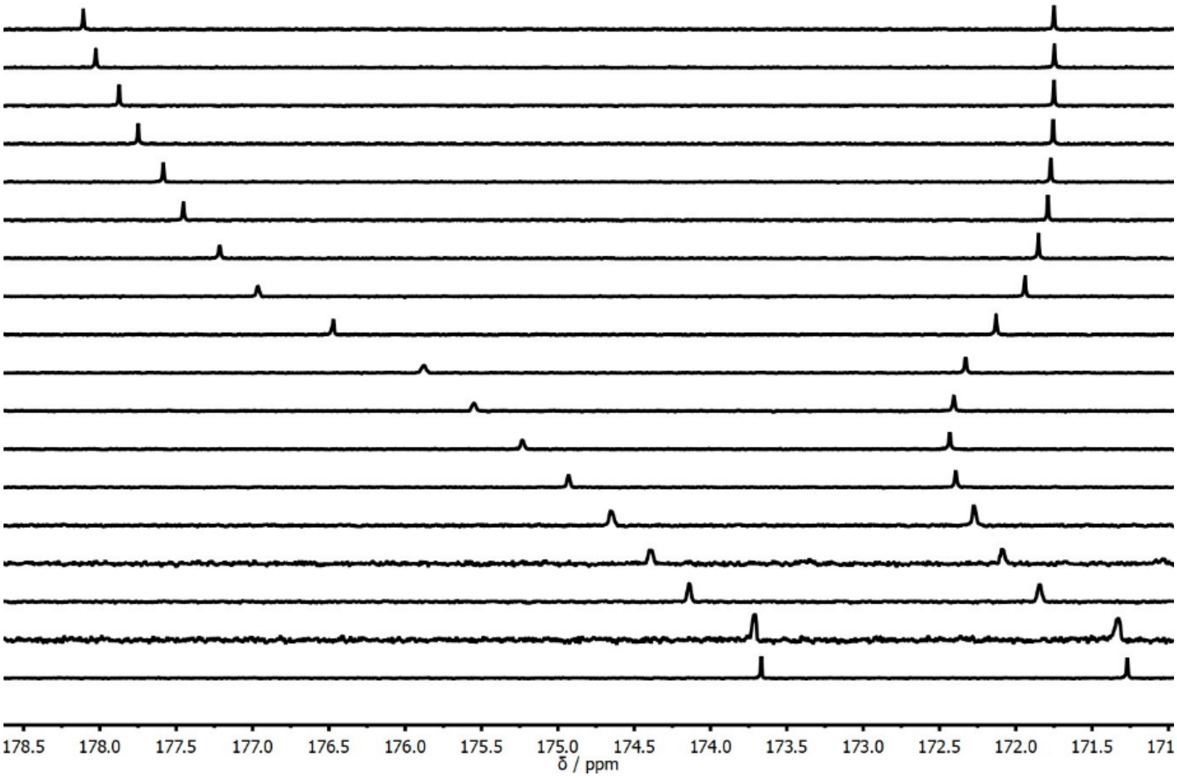

Figure S.37– Stack for the 176 MHz ^{13}C NMR titration of PFTB into **3** (52 mM) in *n*-octane at 298 K

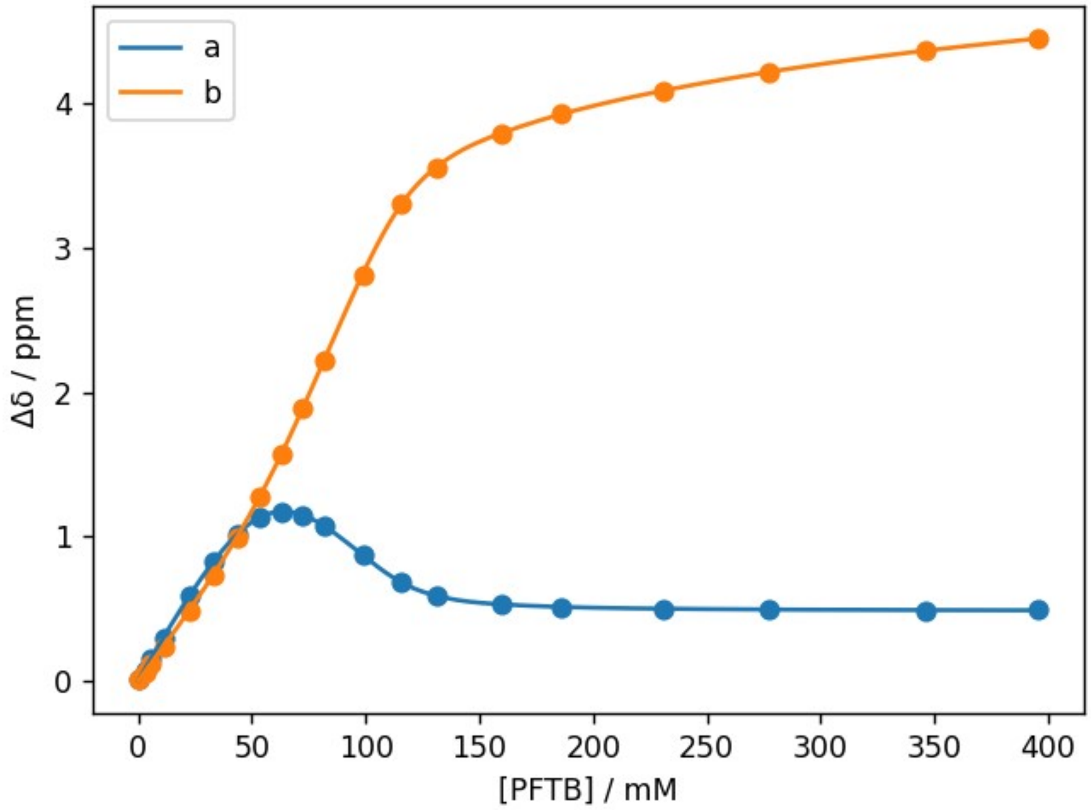

Figure S.38 – Fit of the chemical shifts for the ^{13}C NMR titration of PFTB into **3** (52 mM) in *n*-octane at 298K to a 1:3 binding isotherm.

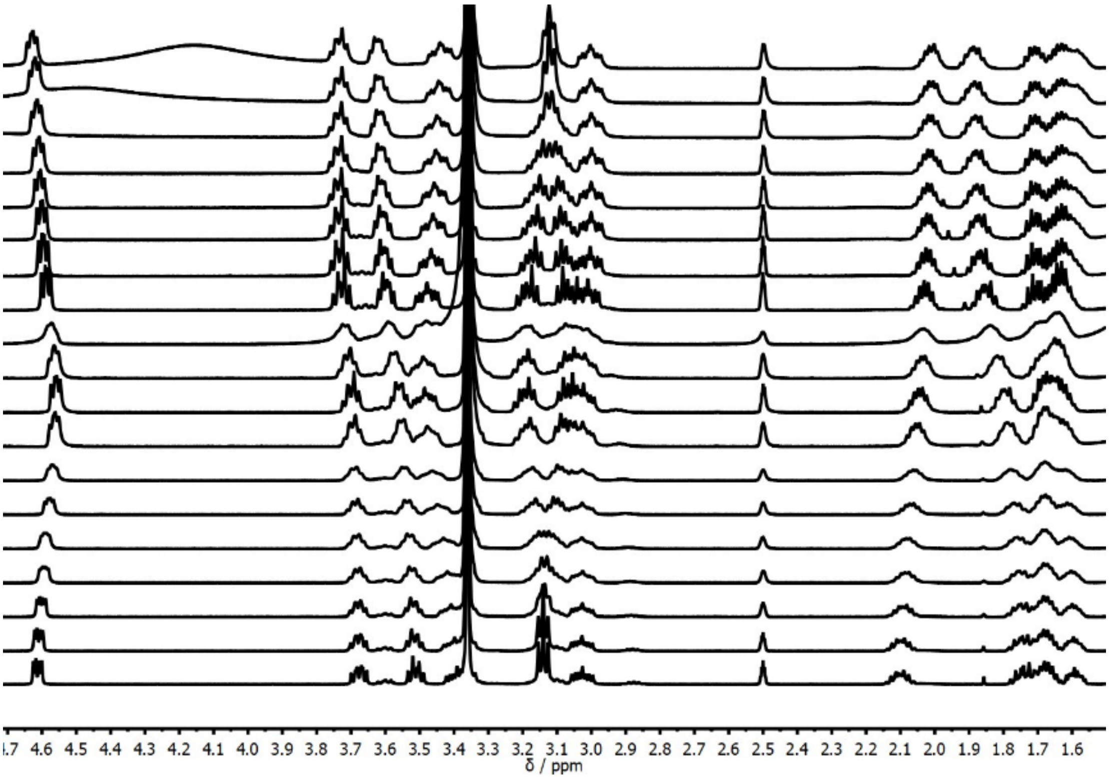

Figure S.39 - 500 MHz ^{1}H NMR spectra for titration of PFTB oxide into **3** (9.0 mM in *n*-octane, at 298 K)

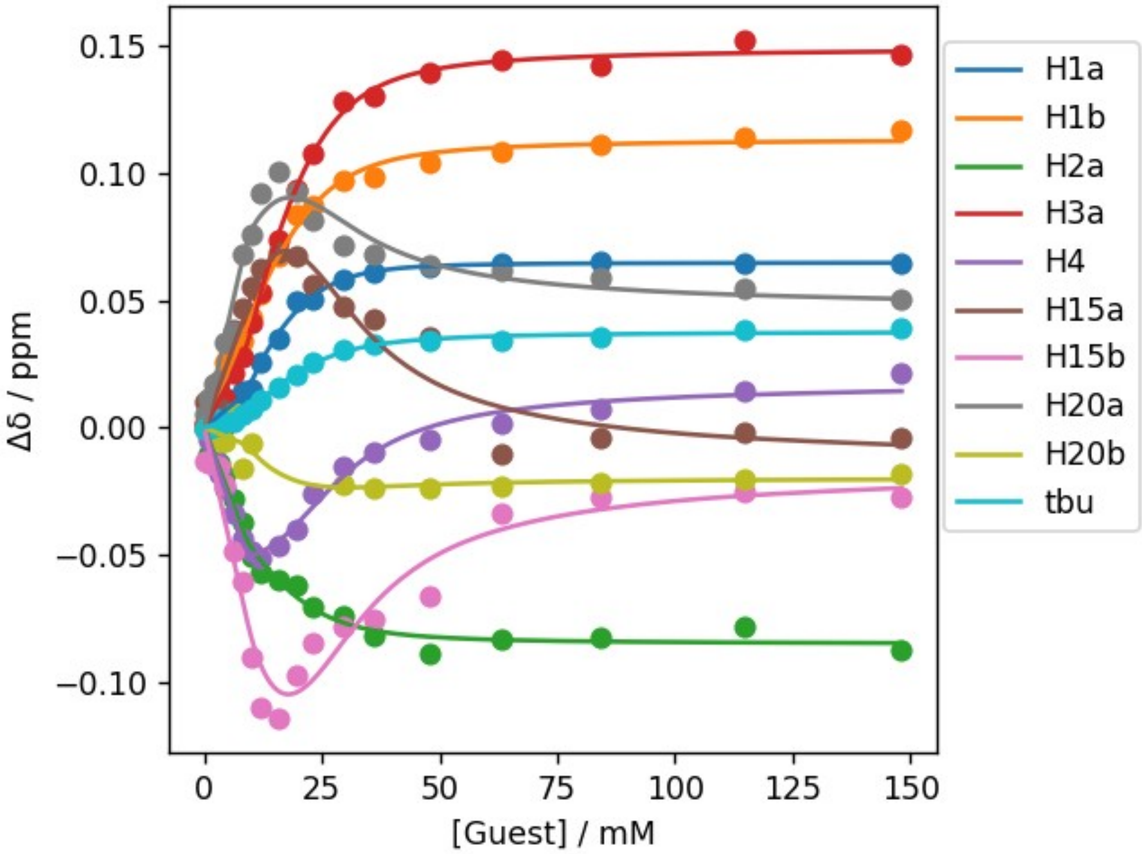

Figure S.40 - Fit of the chemical shifts for the ^{1}H NMR titration of PFTB into **3** (9.0 mM in cyclohexane-d_{12}, at 298 K) to a 1:3 binding isotherm

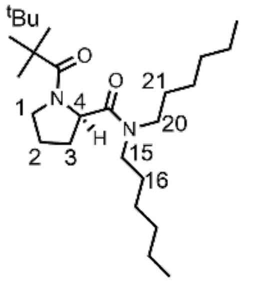

Figure S.41 – Proton labelling scheme for compound **3**.

Proton	$\Delta\delta_1$	$\Delta\delta_2$	$\Delta\delta_3$
1a	+0.02	+0.05	0.00
1b	+0.04	+0.05	+0.02
2a	-0.05	-0.03	-0.01
3a	+0.03	+0.09	+0.03
4	-0.05	0.00	+0.07
15a	+0.04	+0.08	-0.14
15b	-0.07	-0.10	+0.16
20a	+0.08	+0.05	-0.08
20b	-0.01	-0.03	+0.02
^{t}Bu	0.00	+0.02	+0.01

Table S.1 – Limiting shift changes of the protons of compound **3** in ^{1}H NMR titration with PFTB on going from free host to the 1:1 complex, $\Delta\delta_1$, from the 1:1 complex to the 1:2 complex, $\Delta\delta_2$, and from the 1:2 complex to the 1:3 complex, $\Delta\delta_3$. Labels correspond to Figure 7 with a and b being pairs of diastereotopic protons.

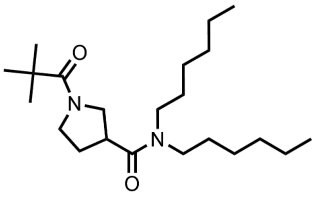

4

Figure S.42 – Structure of the compound **4**

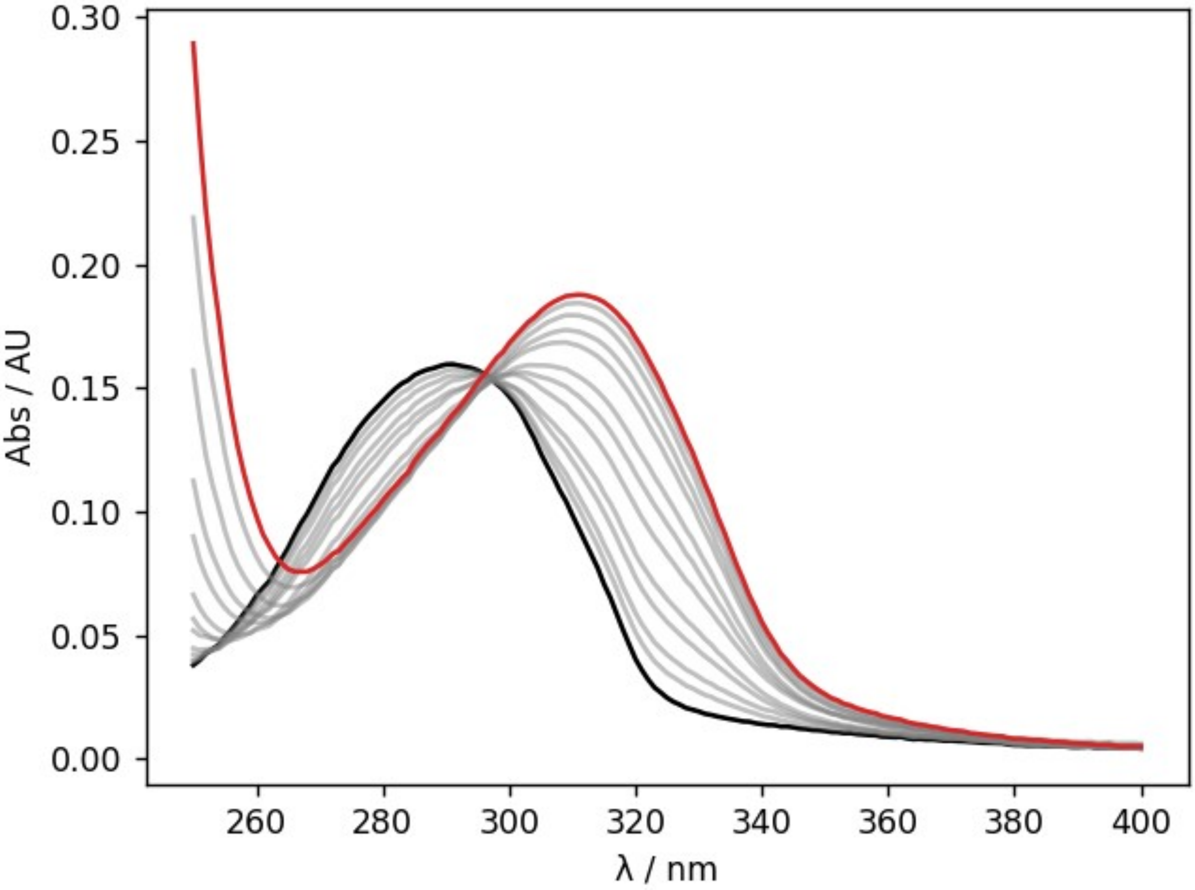

Figure S.43 - UV/Vis absorption spectra for the titration of **4** into 2-methyl-4-nitrophenol (0.015 mM in *n*-octane, at 298K). The UV/Vis spectrum of 2-methyl-4-nitrophenol and the final point of the titration are reported in black and in red, respectively.

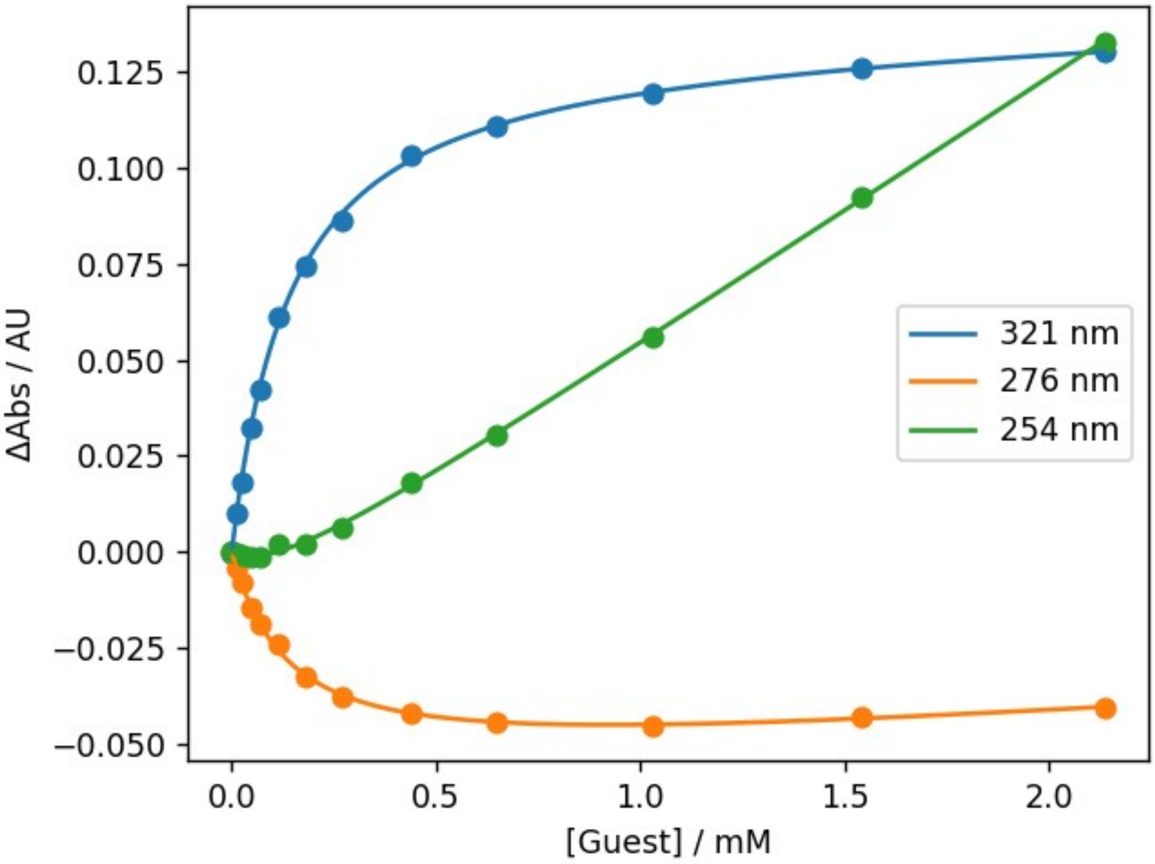

Figure S.44 - The fit of the absorbance at selected wavelengths to a 1:1 binding isotherm accounting for guest absorption for the titration of **4** into 2-methyl-4-nitrophenol (0.015 mM in *n*-octane, at 298 K).

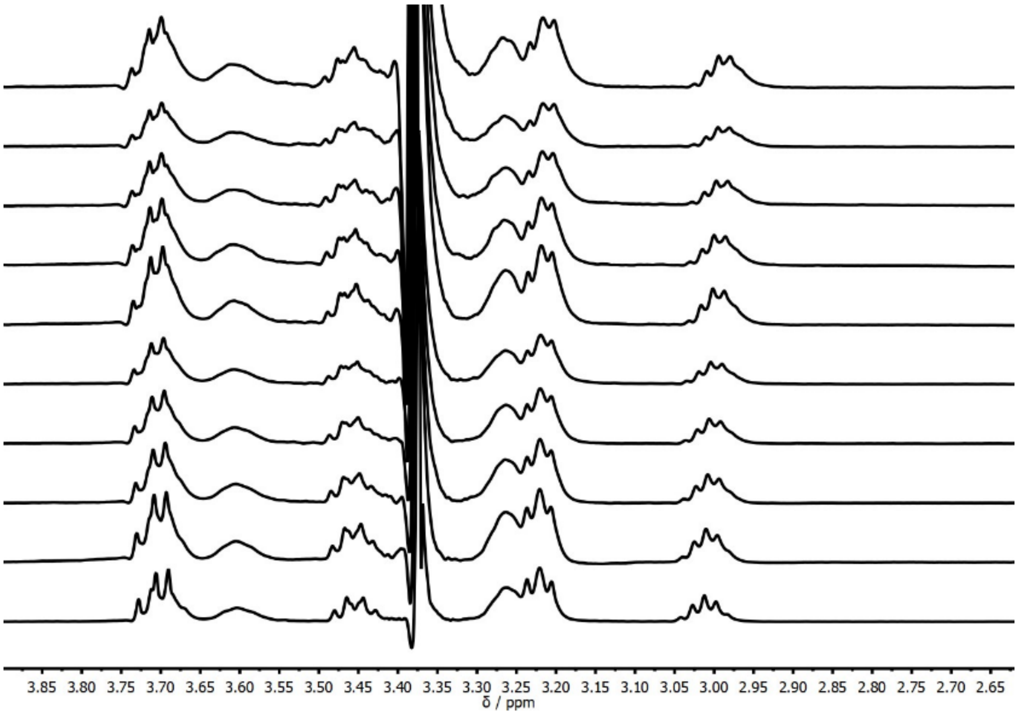

Figure S.45 – Stack plot for the 500 MHz ^{1}H NMR dilution of **4** in *n*-octane with concentration ranging from 22 mM (bottom) to 3.3 mM (top). Artefact at 3.37 ppm is from water in the DMSO-d_6 capillary

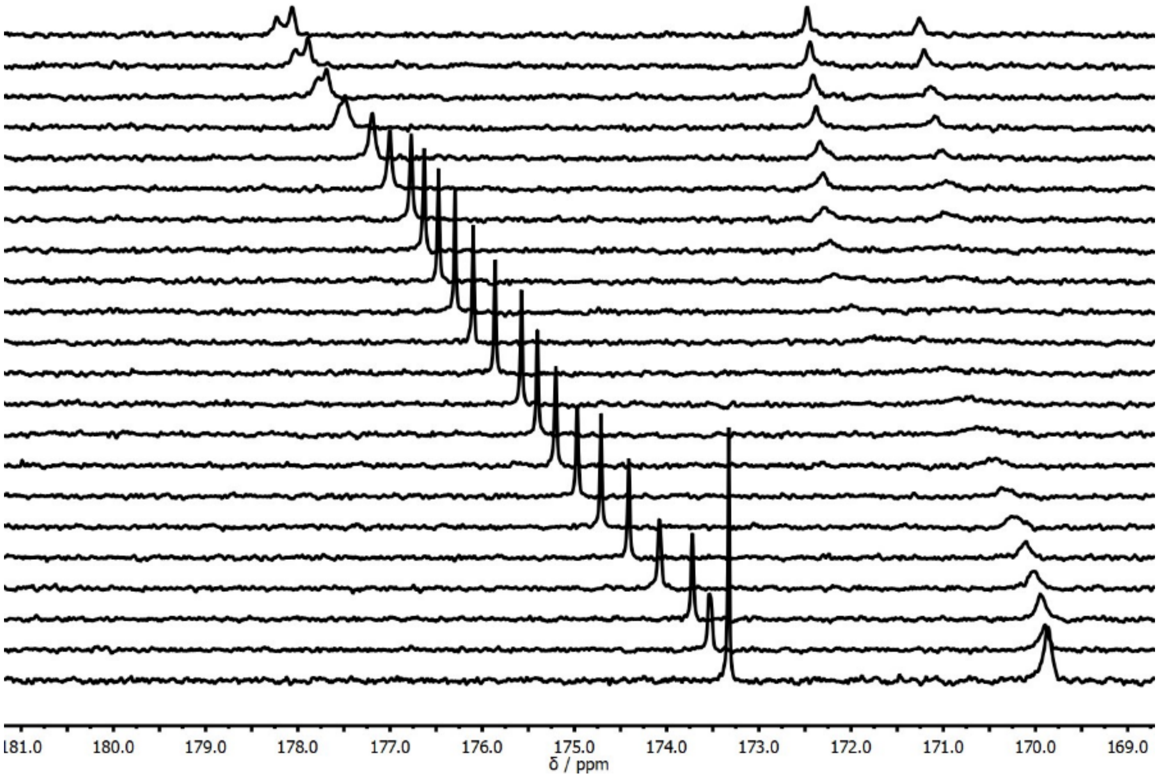

Figure S.46 – Stack for the 176 MHz ^{13}C NMR titration of PFTB into **4** (90 mM) in *n*-octane at 298 K

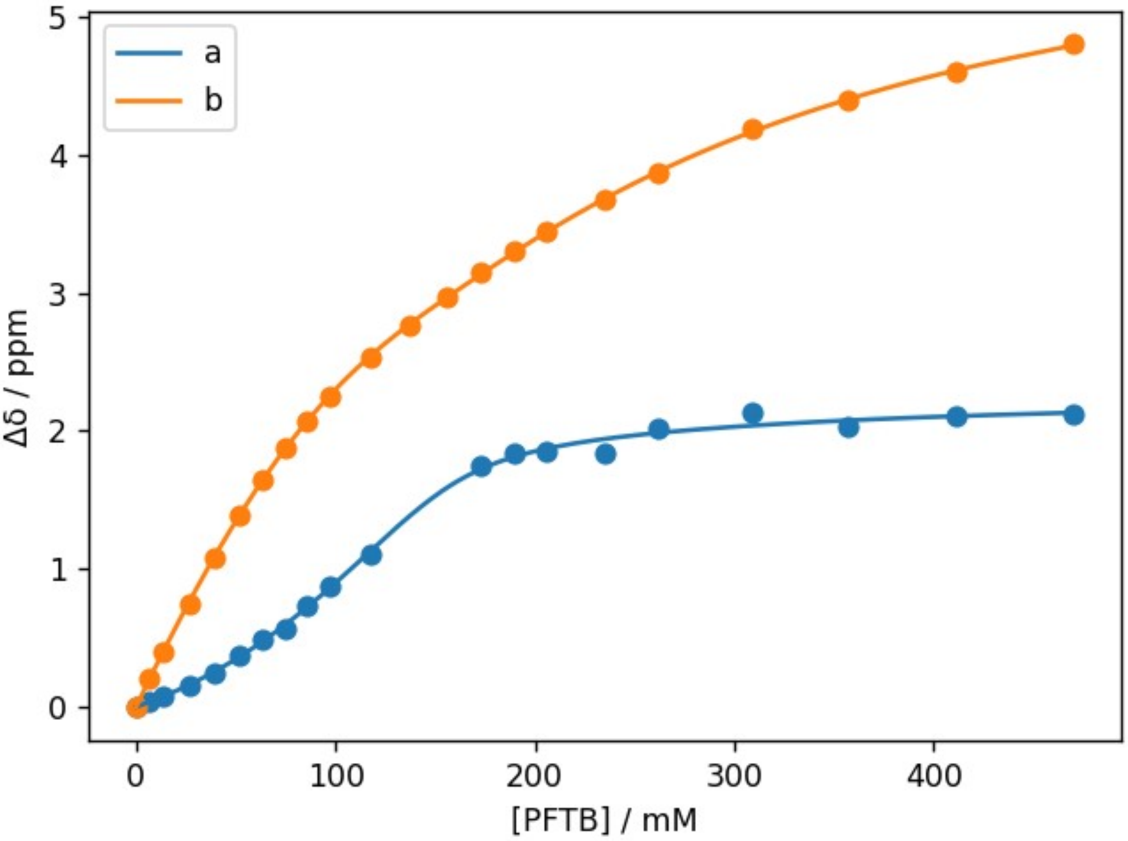

Figure S.47 – Fit of the chemical shifts for the ^{13}C NMR titration of PFTB into **4** (90 mM) in *n*-octane at 298K to a 1:3 binding isotherm.

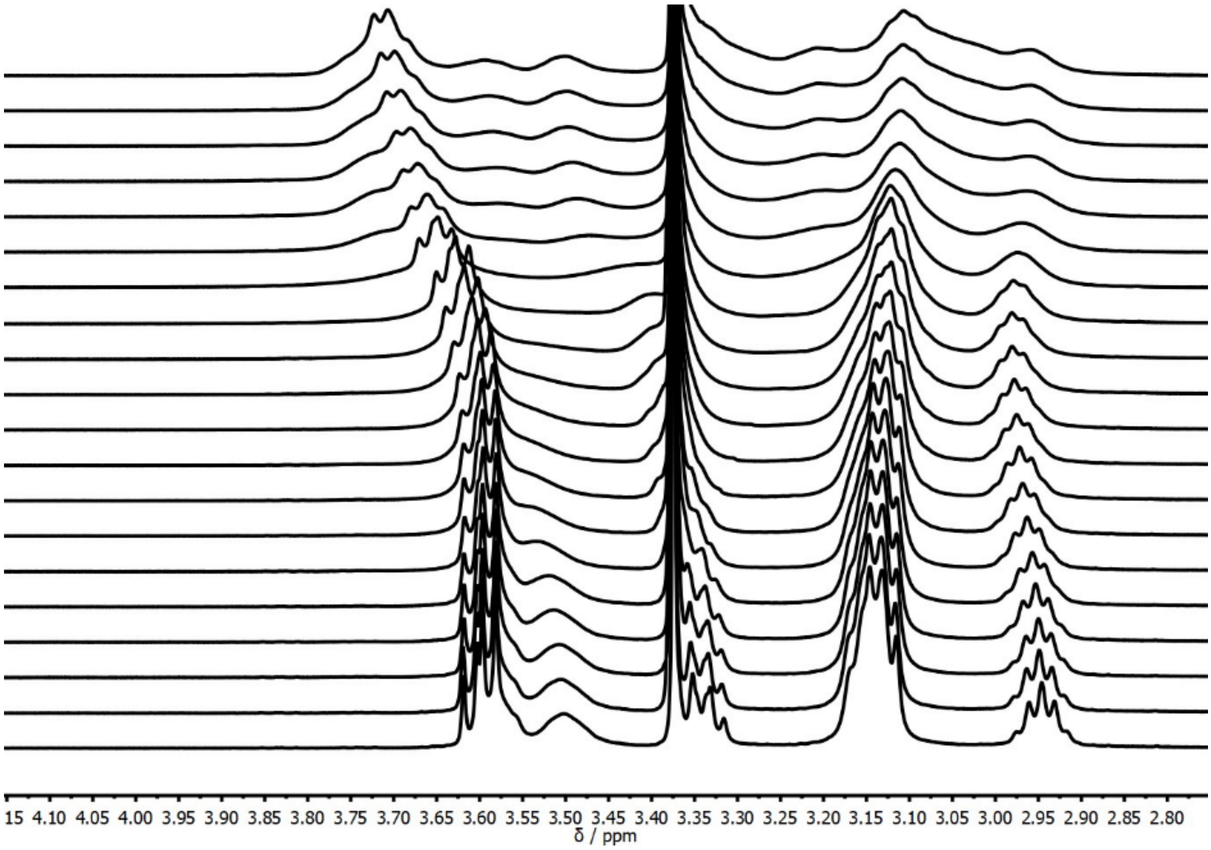

Figure S.48 - 500 MHz ^{1}H NMR spectra for titration of PFTB oxide into **4** (73 mM in cyclohexane-d_{12}, at 298 K) (b)

^{13}C NMR Titration Shift Changes

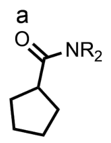

1

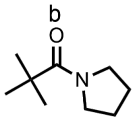

2

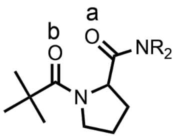

3

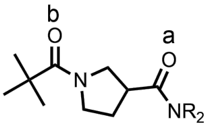

4

Figure S.49 – Compounds **1**-**4**. Two different types of carbonyl group are labelled a and b. R = *n*-hexyl.

			complexes					
Host	Carbonyl	free	1:1	1:2	1:3	$\Delta\delta_1$	$\Delta\delta_2$	$\Delta\delta_3$
**1**	a	173.0	175.9	178.5		+2.88	+2.58	
**3**	a	171.3	172.9	171.7	171.7	+1.61	-1.13	0.00
**4**	a	169.9	170.3	171.8	172.2	+0.41	+1.52	+0.60
**2**	b	173.1	175.9	179.5		+2.84	+3.62	
**3**	b	173.7	174.9	177.4	179.0	+1.22	+2.49	+1.59
**4**	b	173.3	175.6	176.2	179.8	+2.40	+0.57	+3.14

Table S.2 - ^{13}C NMR chemical shifts for complexes formed with PFTB in *n*-octane at 298 K, and stepwise complexation-induced changes in chemical shift.

Single-Crystal X-Ray Diffraction

Single-crystal X-ray diffraction data were collected on a Bruker D8-QUEST diffractometer, equipped with an Incoatec IμS Cu microsource (l = 1.5418 Å) and a PHOTON-III detector operating in shutterless mode. The temperature was controlled at 180(2) K using an Oxford Cryosystems open-flow N_2 Cryostream. The control and processing software was Bruker *APEX5*. Diffraction images were integrated using *SAINT* in *APEX5* and a multi-scan correction was applied using *SADABS*. The final unit-cell parameters were refined against all reflections over the full data range. Structures were solved using *SHELXT* and refined using *SHELXL*. The crystal structures were visualized using *Mercury* and additional geometrical analysis was made using *SHELXL* and *PLATON*.[3-6]

Table S.3 - Crystal structure and refinement details

	**2**	**5**	**6**
CCDC number			
Cambridge data no.	CH_B1_0080	CH_B1_0081	CH_B1_0083
Chemical formula	$C_9H_{17}NO$	$C_{14}H_{24}N_2O_2$	$C_{14}H_{24}N_2O_2$
Formula weight	155.23	252.35	252.35
Temperature / K	180(2)	180(2)	180(2)
Crystal system	monoclinic	orthorhombic	triclinic
Space group	P 2_1/n	P 2_1 2_1 2_1	P –1
a / Å	6.0753(2)	5.7109(2)	5.9843(2)
b / Å	19.1206(8)	14.3218(5)	9.7264(4)
c / Å	8.3541(3)	17.0215(6)	12.6732(5)
alpha / °	90	90	73.160(2)
beta / °	109.392(2)	90	78.918(2)
gamma / °	90	90	86.645(2)
Unit-cell volume / $Å^3$	915.39(6)	1392.19(8)	692.85(5)
Z, Z′	4, 1	4,1	2, 1
Calc. density / g cm^{-3}	1.126	1.204	1.210
F(000)	344	552	276
Radiation type	Cu K[illegible]	Cu K[illegible]	Cu K[illegible]
Absorption coefficient / mm^{-1}	0.567	0.640	0.643

Crystal size / mm^3	0.25 x 0.05 x 0.02	0.25 x 0.10 x 0.04	0.20 x 0.06 x 0.04
2-Theta range / °	9.25-136.75	12.10-136.30	7.41-136.72
Completeness to max 2-theta	0.997	0.994	0.991
No. of refl. measured	13599	9121	10181
No. of independent refl.	1680	2523	2512
R(int)	0.0559	0.0399	0.0350
No. parameters / restraints	103 / 0	166 / 0	176 / 0
Final R1 values (I > 2[illegible](I))	0.0419	0.0342	0.0445
Final wR(F^2) values (all data)	0.1152	0.0843	0.1260
Goodness-of-fit on F2	1.067	1.080	1.075
Largest difference peak & hole / e $Å^{-3}$	0.242, -0.169	0.139, -0.190	0.337, -0.330
Flack parameter		-0.08(13)	

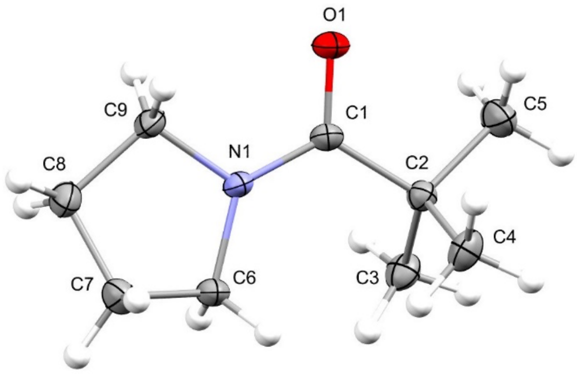

Fig S.50 - Molecular structure of **2** with displacement ellipsoids at 50% probability for non-H atoms.

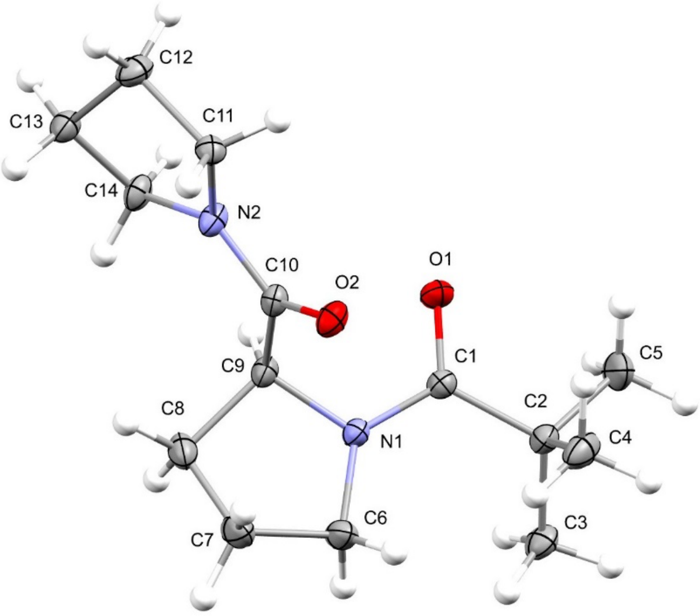

Fig S.51 - Molecular structure of **5** with displacement ellipsoids at 50% probability for non-H atoms.

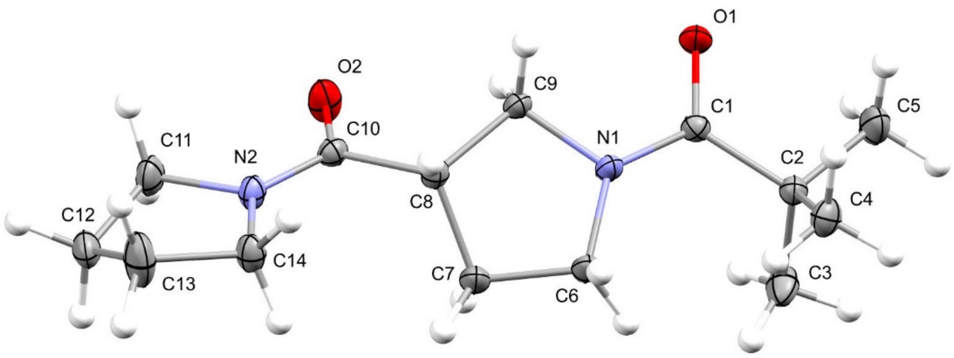

Fig S.52 - Molecular structure of **6** with displacement ellipsoids at 50% probability for non-H atoms. A minor disorder component for the terminal pyrrolidine ring is not shown.

Ab inito calculations

All molecular mechanics calculations were done using Schrödinger's Maestro software (2016 Edition), with $CHCl_3$ as the implicit solvent and MMFFs as the forcefield of choice.[7] First a minimisation calculation was carried out (10000 iterations, convergence on gradient of 0.01). The minimised structure was then used as the basis for a conformational search (mixed torsional/low mode sampling, 10000 steps). The lowest energy conformer was then used as a basis for a Jaguar DFT optimisation using a 6-31G* basis set,[8-10] a B3LYP functional[11-14] and a nonrelativistic Hamiltonian in the gas phase.

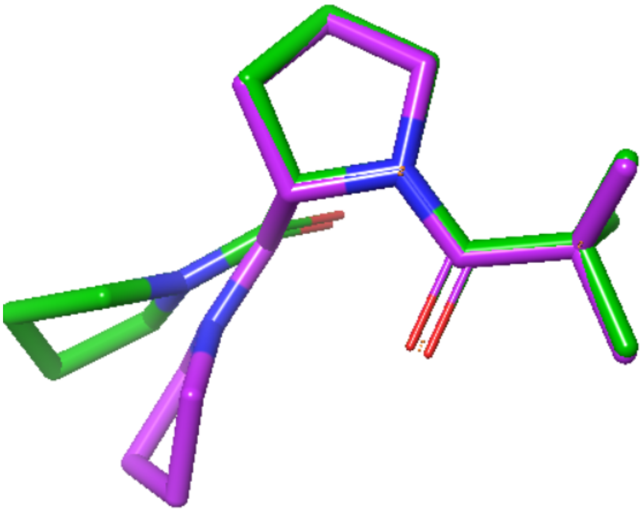

Figure S.53 - Overlay of the DFT optimised structure of **5** (purple) and the crystal structure of **5** (green).

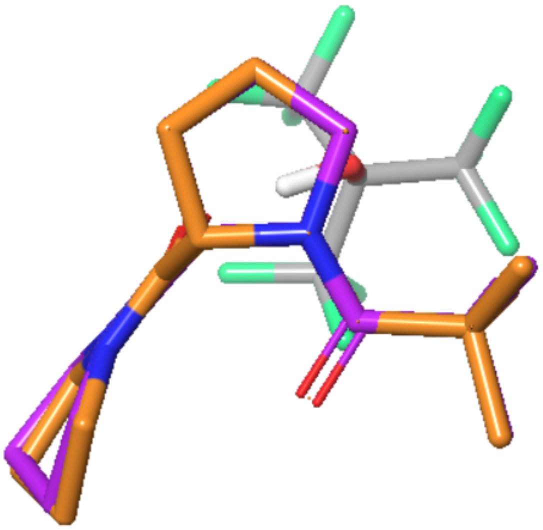

Figure S.54 - Overlay of the DFT optimised structure of free **5** (purple) and the DFT optimised structure of the 1:1 complex of **5** (orange) and PFTB (grey).

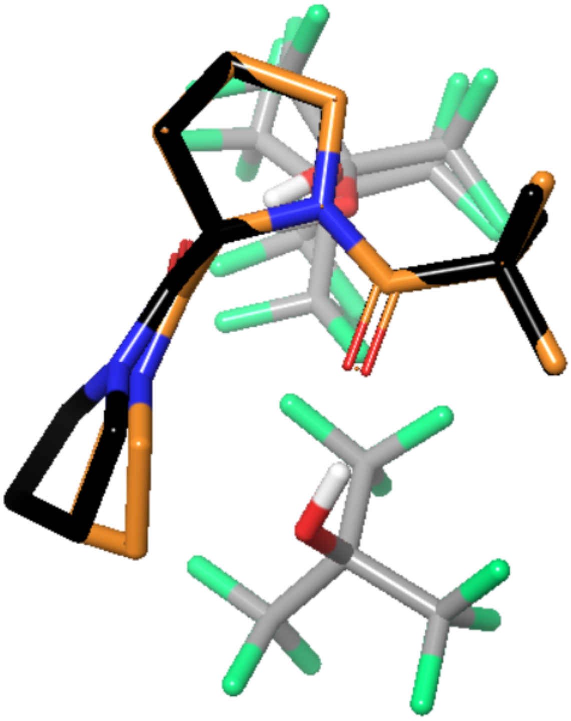

Figure S.55 - Overlay of the DFT optimised structure of the 1:1 complex of **5** (orange) and PFTB (grey) and the DFT optimised structure of the 1:2 complex of **5** (black)and PFTB (grey).

References

1. Fulmer, G.R., Miller, A.J.M., Sherden, N.H., Gottlieb, H.E., Nudelman, A., Stoltz, B.M., Bercaw, J.E., and Goldberg, K.I., *Organometallics,* **2010**, 29, 2176-2179
2. D. O. Soloviev and C. A. Hunter, *Chem. Sci.*, 2024, **15**, 15299–15310
3. Macrae, C. F., Sovago, I., Cottrell, S. J., Galek, P. T. A., McCabe, P., Pidcock, E., Platings, M., Shields, G. P., Stevens, J. S., Towler, M., Wood, P.A., *J. Appl. Cryst.*, **2020***,* 53*,* 226-235
4. Sheldrick, G.M., *Acta Cryst. Sect. A*, **2015**, 71, 3-8
5. Sheldrick, G.M., *Acta Cryst. Sect. C*, **2015**, 71, 3-8
6. Spek A. L., *Acta Cryst. Sect. D*, **2009**, 65, 148-155
7. Halgren, T.A., *J. Comput. Chem.*, **1999**, 20, 720-729
8. Ditchfield, R., Hehre, W. J., Pople, J. A., *J. Chem. Phys.*, **1971**, 54, 724-728
9. Hehre, W. J., Ditchfield, R., Pople, J. A., *J. Chem. Phys.*, **1972**, 56, 2257-2261
10. Hariharan, P.C., Pople, J.A., *Theor. Chim. Acta.*, **1973**, 28, 213-222
11. Becke, A.D., *J. Chem. Phys.*, **1993**, 98, 5648-5652
12. Lee, C., Yang, W., Parr, R.G., *Phys. Rev. B*, **1988**, 37, 785-789
13. Vosko, S.H., Wilk, L., Nusair, M., *Can. J. Phys.*, **1980**, 58, 1200-1211
14. Stephens, P.J., Devlin, F.J., Chabalowski, C.F., Frisch, M.J., *J. Phys. Chem*., **1994**, 98, 11623-11627
